# Amplicon-based prediction of secondary metabolic potential of microbiomes facilitates natural product discovery

**DOI:** 10.1016/j.synbio.2025.10.012

**Published:** 2025-11-04

**Authors:** Zhen-Yi Zhou, Qun-Jian Yin, Buddha Bahadur Basnet, Zi-Yang Li, Gen Li, Mahmoud Emam, Qi-Hao Wu, Hong Wang, Bin Wei

**Affiliations:** a College of Pharmaceutical Science & Collaborative Innovation Center of Yangtze River Delta Region Green Pharmaceuticals, Zhejiang University of Technology, Hangzhou, 310014, China; b Fourth Institute of Oceanography, Ministry of Natural Resources, Beihai, 536003, China; c Central Department of Biotechnology, Tribhuvan University, Kathmandu, 44613, Nepal; d Phytochemistry and Plant Systematics Department, National Research Centre, Dokki, Giza, 12622, Egypt; e Department of Pharmaceutical Sciences, University of Pittsburgh, Pittsburgh, PA, 15261, USA

**Keywords:** PSMPA, Natural products, Biosynthesis gene clusters, 16S rRNA, Maripanthiones

## Abstract

The rapid exploration of natural products not only expands chemical diversity but also plays a vital role in drug discovery by identifying bioactive agents (e.g., antibiotics), revealing novel biological mechanisms, and providing new therapeutic opportunities. However, traditional techniques require extensive resources to screen and prioritize bacteria capable of producing valuable compounds from complex environmental samples. Here, we introduce the PSMPA (**P**redicting the **S**econdary **M**etabolism **P**otential using **A**mplicons) pipeline, designed to assess the secondary metabolic potential of microbiomes by estimating the abundance of biosynthetic gene cluster (BGC) classes based on 16S rRNA gene amplicons. This approach facilitates early-stage selection of samples and strains with high potential for novel compound production. We applied PSMPA to microbiome samples from marine environments (seawater, mangrove sediment, and intertidal flat sediment), leading to the identification of a promising *Marinobacterium* strain YM272 and the discovery of maripanthiones, a new class of sulfur-containing natural products. By enabling early identification of promising strains or samples, PSMPA accelerates novel natural product discovery and enhances the utility of amplicon sequencing for functional analysis. The web version of PSMPA is available at https://www.psmpa.net/.

## 1 Introduction

Microorganisms have long served as invaluable sources for bioactive natural products, many of which have successfully advanced into clinically approved drugs [1]. Historically, discoveries peaked during the mid-20th century, but subsequently declined as pharmaceutical interests shifted away from traditional methods [1,2]. A transformative shift occurred with the realization that microbial natural products are biosynthesized by genes organized into distinct clusters, setting the stage for genome-based exploration [1,3]. With the advent of Next-Generation Sequencing (NGS) in the early 2000s and powerful bioinformatic genome mining tools such as antiSMASH (antibiotics & Secondary Metabolite Analysis Shell) [4], PRISM (PRediction Informatics for Secondary Metabolomes) [5], ClusterFinder [6], and DeepBGC [7], researchers can now rapidly and comprehensively identify novel biosynthetic gene clusters (BGCs) directly from microbial genomic data. Recent progress in decoding microbial secondary metabolite BGCs significantly accelerates the discovery of novel structure from natural ecosystems [[8], [9], [10], [11]]. Meanwhile, systematic analyses of bacterial natural product potential suggest that there is still a vast chemical space yet to be explored [[12], [13], [14]]. A key challenge is identifying microbiome-derived microorganisms with high potential for novel natural product discovery. Although analyzing cultured genomes or metagenome-assembled genomes (MAGs) is effective, their high cost hinders large-scale early-stage screening.

Marker-gene sequencing, such as 16S rRNA amplicon sequencing, is a cost-effective and widely used method for microbial taxonomic identification and profiling, commonly employed to assess microbiota composition in complex environmental samples [[15], [16], [17], [18], [19]]. With the rapid surged in microbial sequenced genome datasets, several well-known tools such as PICRUSt (Phylogenetic Investigation of Communities by Reconstruction of Unobserved States) [20,21], Tax4Fun [22], PanFP (Pangenome-based Functional Profiles) [23], and Piphillin [24], have been developed and widely used to predict the functional potential of the microbe using marker genes. In the meanwhile, the accuracy of predicting metagenomes functions has been improved [25]. For example, the performance of PICRISt2 has been significantly improved as its default genome dataset has 41,926 bacterial and archaeal genomes, which is about 20 times larger than that of PICRUSt1 [21]. These findings highlight the reliability of predicting genomic features from the marker genes in the context of the rapid accumulation of microbial genomic data.

In recent years, an increasing number of BGC databases have emerged, facilitating the mining and analysis of gene clusters and revealing many genera with significant secondary metabolic potential [26,27]. To enable early-stage evaluation of the secondary metabolism potential of microbiome samples and their constituent microorganisms, we developed PSMPA (**P**redicting the **S**econdary **M**etabolism **P**otential using **A**mplicons). PSMPA leverages a comprehensive bacterial BGC atlas as its core reference. Based on this atlas and 16S rRNA amplicon data, PSMPA estimates BGC abundance in microbial communities using PICRUSt2 and BLASTn-based methods. It can serve as a downstream tool in QIIME2 (Quantitative Insights Into Microbial Ecology 2) [25], a widely used platform for 16S rRNA amplicon data analysis. PSMPA directly accepts input files (e.g., feature sequences or tables) generated by QIIME2, streamlining the transition from taxonomic analysis to biosynthetic potential assessment. The accuracy of PSMPA in predicting BGC composition from marker genes under various conditions was evaluated using a paired dataset comprising 16S rRNA gene sequences and corresponding genome sequences.

PSMPA was applied to investigate the BGC profiles of diverse microbiome samples from diverse environmental sources. This comprehensive profiling enabled comparative analyses to identify promising microbiome samples or constituent taxa with high potential for producing novel natural products. As a case study, PSMPA was used to predict the BGC composition of samples collected from mangrove sediment—an ecosystem known for its microbial diversity. Based on these predictions, we isolated a strain of *Marinobacterium*, designated YM272, which showed 97.71 % similarity to *Marinobacterium mangrovicola*. This strain was selected based on its relatively rich predicted BGC content, indicating a high potential to produce novel secondary metabolites. Subsequent fermentation and isolation led to the identification of a new class of sulfur-containing natural products, maripanthiones A–D (**1**–**4**). These findings highlight the utility of PSMPA as an informative tool for accelerating the discovery of novel bioactive compounds from complex microbial communities.

## 2 Materials and methods

### 2.1 Collection and processing of bacterial BGC atlas data

A total of 220,524 bacterial genomes were obtained from the NCBI RefSeq database using the NCBI Datasets command-line tools (v15.3.0), along with associated metadata, including taxonomic information, assembly level, and CheckM quality estimates. Genomes were filtered based on CheckM (v1.2.3) [28] results to retain only those with an estimated completeness of ≥90 % and contamination of ≤5 %. 16S rRNA sequences were extracted from downloaded GBFF files using a custom Python script by identifying features annotated as “16S ribosomal RNA”. For genomes with multiple hits, the longest sequence was retained. If no 16S rRNA annotations were present, genomes were re-annotated using Prokka (v1.14.6) [29] to retrieve 16S rRNA sequences. Genomes lacking identifiable 16S rRNA were excluded. 16S rRNA sequences containing non-ATCG characters or with lengths outside 250 to 2000 base pairs were removed, along with their corresponding genomes. For the collection of taxonomic information, we used TaxonKit (v0.8.0) [30] to obtain specific taxonomic lineage based on the taxonomy id of the genome. Ultimately, a total of 175,321 high-quality bacterial genomes with corresponding 16S rRNA sequences were retained. These genomes were analyzed using antiSMASH (v5.2.0) [4] with default parameters to identify and annotate BGCs. A custom Python script was used to extract the number of BGCs per class for each genome, generating a BGC abundance table. Finally, the BGC abundance table was linked to the corresponding 16S rRNA sequences to construct a comprehensive phylogeny-informed BGC atlas.

### 2.2 Validation analysis of the 16S rRNA gene as a marker for predicting BGC abundance

To evaluate the validity of the 16S rRNA gene as a marker for predicting BGC abundance, we performed a series of clustering and similarity analyses based on the collected 16S sequence dataset. Identical 16S rRNA sequences were first removed using SeqKit (v0.16.1) [31], and duplicate sequences were separately recorded for further evaluation. Clustering of the 16S rRNA sequence dataset was performed using CD-HIT (v4.8.1) [32] at multiple similarity thresholds ranging from 80 % to 99 %. We first assessed the consistency of BGC abundance among genomes sharing identical 16S rRNA sequences. Subsequently, for each set of clusters generated at a given CD-HIT threshold, we selected non-singleton clusters and evaluated the intra-cluster similarity of BGC abundance. Due to the diversity and hybrid nature of antiSMASH BGC classifications, for simplification, these classifications were grouped into eight major BGC classes according to the BiG-SCAPE (v1) standard (More details are shown in Supplementary Information) [33]. The abundance of eight major BGC classes was used to construct a feature vector for each genome. To quantify the intra-cluster similarity of BGC abundance profiles, we designed a position-wise similarity scoring function defined as follows:

$$Similarity=\frac{1}{n}\sum_{i=1}^{n}\frac{1}{\left|a_{i}-b_{i}\right|+1}$$

Where, *n* is the number of BGC classes considered (here is 8, the length of the feature vector); *a*_*i*_ and *b*_*i*_ are the abundance values of the *i*-th BGC class in genome A and genome B, respectively. Pairwise similarity was calculated for all genome pairs within each 16S cluster. Five statistical descriptors—mean, median, standard deviation, minimum, and maximum—were computed to quantify intra-cluster similarity in BGC composition.

### 2.3 Establishment of PSMPA

PSMPA provides two methods to predict the BGC profiles of microbiomes using 16S rRNA gene sequences. The first method (psmpa1) is based on the PICRUSt2 framework [21]. In this approach, input 16S rRNA gene sequences are placed into a reference phylogenetic tree, and the traits of microorganisms without genome sequences are inferred using Hidden State Prediction (HSP) methods, including “mp”, “emp_prob”, “pic”, “scp”, or “subtree_average”. The reference tree comprises 20,000 unique full-length 16S rRNA genes, and the predicted traits correspond to the counts of each BGC class present in the reference bacterial genomes. The second method (psmpa2) employs BLAST (v2.12.0) [34] to align input sequences against a curated 16S rRNA gene dataset, assigning BGC profiles from the top-matched reference sequences within a user-defined similarity threshold. This reference dataset consists of 53,812 unique 16S rRNA sequences, de-replicated from 175,321 bacterial genomes. Because many 16S rRNA genes are shared across genomes and BGC counts can be treated as either discrete or continuous traits, four data processing strategies were implemented to assign BGC values to each 16S rRNA sequence: “mean_float”, “mean_int”, “median_float”, and “median_int”. The primary output is a CSV file containing the predicted counts of each BGC class for every query 16S rRNA sequence. For datasets generated by high-throughput amplicon sequencing, PSMPA calculates the total abundance of each BGC class per sample using the provided feature table and outputs the results in a separate CSV file. To simplify the output, eight major BGC classes defined by BiG-SCAPE standard are used [33].

### 2.4 Accuracy assessment of PSMPA

To evaluate the accuracy of PSMPA under various conditions, a test dataset comprising 5000 bacterial genomes—distinct from those in the PSMPA reference dataset—was randomly selected from the NCBI GenBank database. The predicted BGC counts for each genome were obtained using PSMPA based on their 16S rRNA sequences and compared against the corresponding BGC annotations generated from antiSMASH. Prediction accuracy was quantitatively assessed by calculating the proportion of genomes whose BGC count deviations fell within three bias intervals: [0, 1], (1, 5], and (5, ∞). To investigate the effect of 16S rRNA gene length on predictive accuracy, both full-length sequences and the V3–V4 regions were used as input. Additionally, the influence of sequence novelty on prediction performance was examined by grouping the 16S rRNA genes based on their sequence identity to the reference dataset into four bins: 0–80 %, 80–90 %, 90–95 %, and 95–100 %. Comparative analyses were conducted across these groups to evaluate PSMPA performance and determine the optimal parameter settings for different levels of sequence similarity.

### 2.5 PSMPA-based BGC profiling, strain isolation, and genomic characterization

PSMPA was used to predict BGC abundance in the microbiota from five marine environmental samples: 8MC01 and 8MC02 (seawater), B3A (mangrove sediment), and X2 and S2 (intertidal flat sediment). Raw 16S rRNA amplicon sequencing reads were processed using QIIME2 [25] to generate representative sequences and corresponding feature tables, which served as input for PSMPA. The relative abundance of BGC classes across samples was visualized using a clustering heatmap.

Based on these predictions, potential strains were selected for isolation. A plate dilution method was employed using 2216E medium. Fresh sediment was cultured in 1/10 2216E liquid medium at room temperature (approximately 25 °C) for 24 h. The culture was then serially diluted, and aliquots of the dilutions were streaked onto 2216E agar plates. This streaking process was repeated multiple times to obtain isolated single colonies. Using this approach, a strain belonging to the genus *Marinobacterium*, designated *Marinobacterium* sp. YM272, was successfully isolated from the B3A sample. This strain was further characterized by 16S rRNA gene sequencing, whole-genome sequencing, and BGC annotation using antiSMASH. Genome sequencing was performed using a combination of second-generation (Illumina) and third-generation (Oxford Nanopore) technologies (Kaitai Biotechnology Co., Ltd., Hangzhou, China). Illumina sequencing provided high-accuracy short reads, while Nanopore sequencing generated long reads.

Genome assembly was performed using Flye (v2.9.2-b1786) [35] with multiple parameter sets to optimize assembly quality. The resulting assembly was generated from high-quality Nanopore reads (Q > 10, basecalled using Guppy5 SUP) with the parameters "--nano-hq --read-error 0.2 -g 5 m --asm-coverage 50″, yielded a single contig of 4,462,512 bp. The assembly was polished using Medaka (v1.7.3) with the model “r941_prom_hac_g507″ to correct Nanopore-associated errors. Illumina reads were then aligned to the polished assembly using Bowtie2 (v2.5.1) [36], and Pilon (v1.24) [37] was used for final polishing. The completed genome assembly resulted in a single scaffold with a total length of 4,465,342 bp. Finally, BGCs were annotated using antiSMASH, providing insight into the secondary metabolic potential of *Marinobacterium* sp. YM272.

### 2.6 Prioritization and optimization of culture condition

To explore the metabolic potential of *Marinobacterium* sp. YM272, we tested combinations of four media (M1–M4) and six elicitors (A–F), alongside controls without elicitors (G) and medium-only blanks (H). All media were prepared in artificial seawater with distinct compositions. M1 contained glucose (0.01 %), peptone (0.5 %), and yeast extract (0.1 %). M2 consisted of glucose (0.05 %), sodium pyruvate (0.03 %), soluble starch (0.05 %), peptone (0.05 %), yeast extract (0.05 %), tryptone (0.05 %), MgSO_4_·7H_2_O (0.005 %), and K_2_HPO_4_ (0.03 %). M3 included soluble starch (1 %), peptone (0.03 %), MgSO_4_·7H_2_O (0.005 %), NaNO_3_ (0.2 %), KCl (0.2 %), K_2_HPO_4_ (0.2 %), CaCO3 (0.002 %), and FeSO_4_ (0.001 %). M4 was composed of glucose (0.2 %), soluble starch (2.5 %), peptone (0.5 %), casein (0.5 %), yeast extract (0.5 %), and CaCO_3_ (0.3 %). Six elicitors previously reported to activate silent or weakly expressed BGCs were used: 2 mM LaCl3·7H2O (A), 100 μM NiCl_2_·6H_2_O (B), 10 mM EDTA (C), 3 % DMSO (D), 100 mg/L streptomycin (E), 10 mg/L kanamycin (F) [38,39]. Cultures were incubated at 30 °C, 180 rpm for 2 days, then transferred to 50 mL media in 250 mL flasks under the same conditions. Elicitors were added after 12 h, followed by 5 days of fermentation.

Fermentation broths were extracted with ethyl acetate (2 × volume), and crude extracts were analyzed by LC-MS/MS. Raw data were processed in MZmine 3 [40], and resulting features were uploaded to GNPS for Feature-Based Molecular Networking (FBMN) [41]. Promising compounds were identified by manual comparison of HPLC profiles between fermentation and control groups, UV spectra, and distinct features in TICs or FBMN cluster nodes.

### 2.7 Large-scale fermentation and purification of maripanthiones

Large-scale fermentation (40 L in 40 × 2 L flasks, 1 L per flask) of *Marinobacterium* sp. YM272 was performed using M1 medium supplemented with NiCl_2_ as an elicitor at 30 °C and 180 rpm for 7 days. Post-fermentation, cultures were extracted with ethyl acetate (2× volume), yielding approximately 10 g of crude extract. The crude extract was dissolved in 395 mL H_2_O and 5 mL MeOH, followed by sequential liquid-liquid extraction with 400 mL each of hexane (Hex), dichloromethane (DCM), and ethyl acetate (EtOAc), resulting in four fractions: Hex (Fr. A, 0.6 g), DCM (Fr. B, 2.0 g), EtOAc (Fr. C, 1.5 g), and aqueous (Fr. D, 5.1 g). The target compounds detected in Fr. B by LC-MS (*m/z* 347, 431, 385, 383) were subjected to MCI gel column chromatography using a gradually decreasing polarity solvent system (MeOH/H_2_O), yielding sub-fractions (Fr. B.1–B.6). Taking compound **1** as an example, the target compound (*m/z* 347) detected in sub-fraction Fr. B.3 was subsequently further purified using Sephadex LH-20 size-exclusion chromatography, resulting in sub-fractions (Fr. B.3.A–B.3.E). Finally, compound **1** was isolated using reverse-phase semi-preparative HPLC with a solvent mixture of 15 % MeCN/MeOH (1:1) in water containing 0.001 % TFA. Other compounds (**2**–**4**) were isolated using a similar strategy, with appropriate adjustments in the solvent system and fraction collection, leading to the isolation of maripanthiones A (**1**, 7.3 mg), B (**2**, 1.0 mg), C (**3**, 3.2 mg), and D (**4**, 1.5 mg). More detailed experimental instrumentation and conditions are listed in Supplementary Information.

## 3 Results

### 3.1 Phylogeny-informed bacterial BGC atlas

**Fig. 1:**
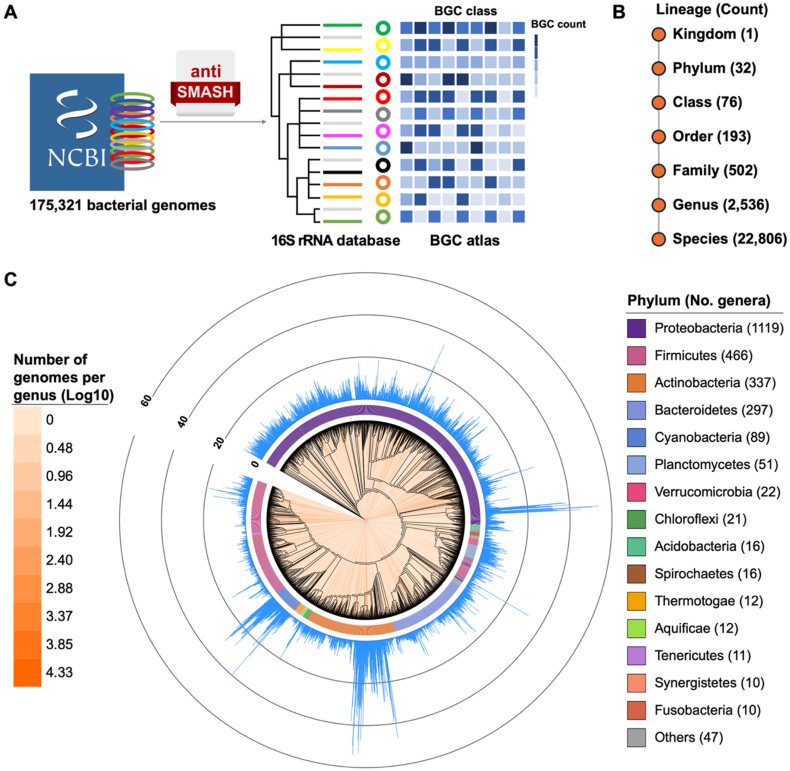
**The phylogeny-informed bacterial BGC atlas.** (**A**) Dataset and workflow for constructing the phylogeny-informed bacterial BGC atlas. (**B**) The number of taxa at each taxonomic level. (**C**) A phylogenetic tree of 2536 bacterial genera, annotated with the average BGC count per genome for each genus. Orange lines along the tree branches indicate the number of genomes in each genus. The 15 genera with the highest genome counts are individually labeled, while remaining genera are grouped as “Others”.

To analyze the relationship between bacterial phylogeny and BGC profiles, we curated a comprehensive phylogeny-informed bacterial BGC atlas by associating 16S rRNA sequences with BGC abundances, based on 175,321 high-quality genomes from NCBI RefSeq (Fig. 1A). The atlas comprises 1119 genera of Proteobacteria, 466 of Firmicutes, 337 of Actinobacteria, 297 of Bacteroidetes, 89 of Cyanobacteria, and 228 genera from 27 other phyla (Fig. 1B, **Data S1**). A genus-level phylogenetic tree was constructed using one representative 16S rRNA sequence per genus (Fig. 1C). The average number of BGCs per genome in each genus, ranging from 0 to 54.

Consistent with previous studies [5,42], Actinobacteria and Cyanobacteria exhibited the highest BGC richness. Notably, 85 genera averaged more than 20 BGCs per genome, and 11 exceeded an average of 40, for instance, *Actinocrispum* currently has only one high-quality genome sequenced, but it contains a remarkable 54 BGCs—the highest average BGC count per genome in bacteria. Although Proteobacteria are generally considered to have lower biosynthetic potential [43], four genera from this phylum—*Enhygromyxa*, *Cystobacter*, *Pyxidicoccus*, and *Corallococcus*—displayed average BGC counts above 40. The genus-level atlas revealed substantial variation in BGC abundance within many genera. Specifically, over 70 % of genera exhibited limited intra-genus variability, with BGC count ranges less than 5. In contrast, a smaller subset showed moderate variation (range 5–20), and only 4 % of genera displayed high variability with ranges exceeding 20. Notably, the genus *Streptomyces* exhibited the largest BGC count range (133), highlighting pronounced biosynthetic diversity among its members (**Data S2**). These findings underscore the importance of systematic exploration of bacterial secondary metabolic potential. Our previously published BGC atlas included 18,043 BGCs from 10,121 genomes [42]. The updated version now incorporates 1,083,283 BGCs from 175,321 genomes, surpassing earlier efforts and highlighting the extraordinary diversity of bacterial BGCs. This expanded atlas provides a valuable resource for the discovery of novel secondary metabolites.

**Fig. 2:**
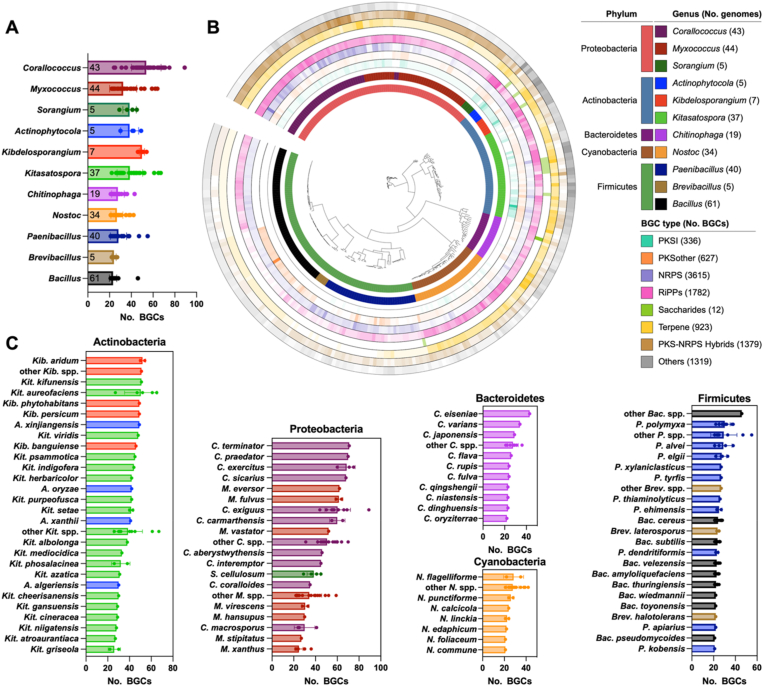
**Catalog of promising strains with high secondary metabolite biosynthetic potential.** (**A**) Bar chart showing the average number of BGCs per genus across 11 BGC-rich genera (Dots represent BGC counts per genome; numbers at the bottom of the bars indicate genome counts per genus). (**B**) A phylogenetic tree of 300 bacterial genomes from these genera, annotated with phylum- and genus-level taxonomy and the distribution of eight major BGC classes. (**C**) Bar charts showing BGC counts for species or species groups across five bacterial phyla.

To illustrate its utility, we identified 11 previously underexplored genera encompassing 300 genomes (each with ≥20 BGCs), spanning five major bacterial phyla: Proteobacteria, Actinobacteria, Bacteroidetes, Cyanobacteria, and Firmicutes. These genera contained between 5 and 61 genomes each, with BGC counts per genome ranging from 20 to 89 (Fig. 2A). The overall BGC composition is summarized in a phylogenetic tree, annotated according to the eight BiG-SCAPE classes: PKSI (Type I polyketide synthases), PKSother (other PKSs), NRPS (nonribosomal peptide synthetases), PKS-NRPS hybrids, RiPPs (ribosomally synthesized and post-translationally modified peptides), saccharides, terpenes, and others (Fig. 2B) [33]. A total of 9993 BGCs were identified across these genomes, with BGC counts per genome ranging from 20 to 89. The five most abundant BGC classes were NRPS (36.2 %), RiPPs (17.8 %), PKS-NRPS hybrids (13.8 %), terpene (9.2 %), and PKSother (6.3 %). Some BGC classes exhibited strong genus specificity. For example, PKS-NRPS hybrids were predominantly found in *Corallococcus* and *Myxococcus* (Proteobacteria), while PKSI clusters were enriched in *Actinophytocola*, *Kibdelosporangium*, and *Kitasatospora* (Actinobacteria). Although these genera ranked among the top three in BGC content within their respective phyla, substantial variation in BGC abundance was observed among species within the same genus. To reflect this intra-genus diversity, we further presented species-level BGC counts across the five phyla (Fig. 2C), thereby providing a catalog of candidate strains with high secondary metabolic potential for future natural product discovery efforts. These insights lay a solid foundation for targeted natural product discovery and highlight the functional diversity of bacterial secondary metabolism. Full BGC composition data for all 175,321 genomes is provided in **Data S1**.

### 3.2 16S rRNA gene as a marker for predicting BGC distribution

**Fig. 3:**
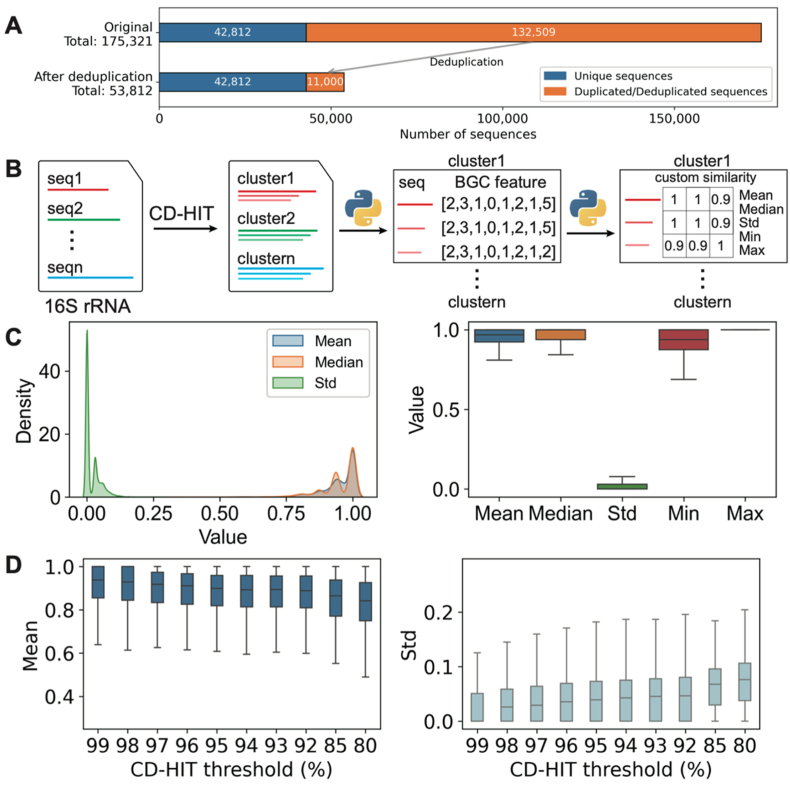
**Statistical analysis of the 16S rRNA sequence dataset and its validation as predictive markers for BGC distribution.** (**A**) Sequence counts before and after dereplication. (**B**) Workflow for correlating 16S rRNA sequences with BGC profiles. (**C**) BGC distribution consistency across genomes sharing identical 16S rRNA sequences. Left: Density plots of similarity matrix statistics (mean, median, standard deviation); Right: Boxplots summarizing these metrics (mean, median, SD, min, max). (**D**) Intra-cluster BGC similarity at varying CD-HIT identity thresholds. Left: Boxplots of mean similarity per cluster (non-singletons); Right: Corresponding standard deviations.

Exploratory analysis of the 16S rRNA dataset in the atlas revealed that 132,509 sequences (75.6 %) were redundant, while 42,812 (24.4 %) were unique. Following deduplication, 53,812 unique representative sequences were retained for downstream analysis (Fig. 3A, Fig. S1). To evaluate the feasibility of using 16S rRNA sequences as predictive markers for BGC abundances, we examined the correlation between 16S rRNA sequence similarity and BGC profile similarity (Fig. 3B). We clustered 16S rRNA sequences at varying identity thresholds via CD-HIT and computed pairwise similarity of BGC profile vectors within each cluster. The resulting similarity scores within each cluster were summarized using key statistical measures—mean, median, standard deviation (Std), minimum, and maximum values—to evaluate intra-cluster consistency in BGC profiles.

We first examined genomes sharing identical 16S rRNA sequences, a scenario commonly encountered in public databases (e.g., NCBI) due to multiple assemblies of well-studied strains. This analysis served as a critical validation of the marker gene approach. The similarity matrices demonstrated near-perfect agreement in BGC distributions: mean and median values approached 1, with minimal deviation (Std ≈ 0). The similarity range was tightly constrained (minimum ≈ 1, maximum = 1), confirming that genomes with identical 16S rRNA sequences harbor virtually indistinguishable BGC repertoires (Fig. 3C).

To further evaluate the robustness of this correlation, we analyzed variations in BGC counts across genomes with different assembly qualities by calculating standard deviations for each assembly level separately (Fig. S2). The average standard deviation for all BGC classes remained close to zero. Among high-quality assemblies (complete and chromosome-level), the proportion of outliers in total BGC counts was less than 3 %. In contrast, when lower-quality assemblies (scaffold and contig-level) were included, the outlier ratio exceeded 7 %, indicating that assembly fragmentation may introduce artifacts in BGC annotation.

Next, we evaluated BGC similarity across 16S rRNA clusters generated at varying identity thresholds using CD-HIT. The intra-cluster BGC similarity exhibited a gradual decline with decreasing clustering thresholds (Fig. 3D). At thresholds ≥97 %, the mean intra-cluster similarity consistently exceeded 0.9, suggesting highly conserved BGC profiles among phylogenetically closely related taxa. Notably, the dispersion of similarity values increased progressively at lower identity thresholds, as reflected by rising standard deviations. These results establish a robust positive correlation between 16S rRNA sequence similarity and BGC profile conservation, providing a biological foundation for the PSMPA prediction framework.

### 3.3 PSMPA: a pipeline for predicting microbial secondary metabolite potential

#### 3.3.1 Description of PSMPA

**Fig. 4:**
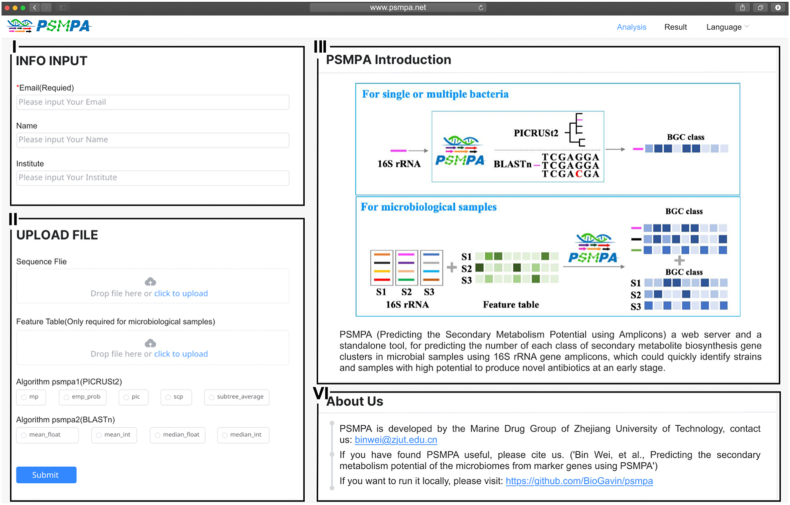
**The pipeline of PSMPA.** Input options: Full-length or representative 16S rRNA sequences are processed through either (i) PICRUSt2-based phylogenetic prediction or (ii) BLASTn alignment against a curated reference dataset to infer BGC distributions. Integration with microbiome analysis: PSMPA accepts QIIME2 outputs (representative sequences and feature tables) to estimate sample-specific BGC class abundances in downstream analyses.

To enable the practical application of the bacterial BGC atlas, we developed PSMPA, a user-friendly pipeline for analyzing microbiome-derived 16S rRNA sequences to estimate secondary metabolic potential (Fig. 4). PSMPA accepts two input formats: (1) FASTA files containing individual or multiple 16S rRNA sequences for single-sample analysis, and (2) paired representative sequences (FASTA) and feature abundance tables (BIOM format) for multi-sample amplicon sequencing data, which can be directly exported from QIIME2 [25]. incorporates two complementary prediction approaches: (1) “psmpa1″, leverages the PICRUSt2 framework to place query 16S rRNA sequences onto a reference phylogenetic tree and infer BGC traits using Hidden State Prediction (HSP) algorithms. (2) “psmpa2″, uses BLASTn to align input sequences against a curated 16S rRNA gene dataset, assigning BGC profiles based on the top-matching reference sequences within a defined identity threshold. BGC count data undergo preprocessing through four optional strategies (mean_float, mean_int, median_float, median_int) to generate either continuous or discrete outputs. For each input, PSMPA generates a CSV file listing the predicted counts for each BGC class. In amplicon-based profiling, it also summarizes the total abundance of each BGC class across samples. For application, the choice between using the V3–V4 region versus full-length 16S rRNA sequencing in PSMPA depends on the research objective and sample type. V3–V4 region sequencing, commonly obtained from high-throughput amplicon studies of environmental microbiomes, is well-suited for estimating BGC class abundance across multiple samples and enables large-scale community-level comparisons with lower cost. In contrast, full-length 16S rRNA sequencing, typically performed on single isolates, is more appropriate for evaluating the biosynthetic potential of isolated individual strains. This can help natural product researchers prioritize cultured isolates for downstream genome sequencing and metabolite discovery. PSMPA supports both modes of input, supporting BGC profiling workflows ranging from broad microbiome analyses to focused evaluations of isolated strains. A web-based version of PSMPA is freely available at https://www.psmpa.net, enabling users to run analyses without writing code.

#### 3.3.2 Accuracy assessment of PSMPA

**Fig. 5:**
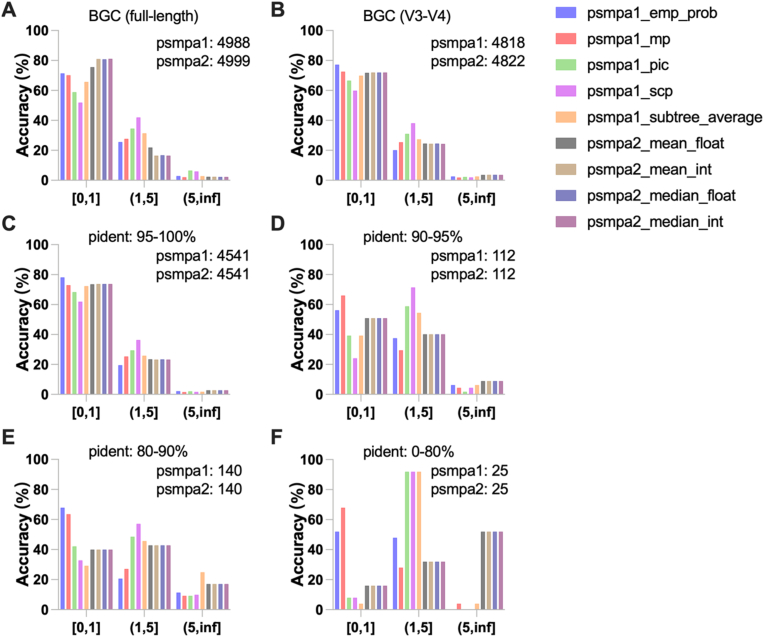
**Performance evaluation of PSMPA in predicting total BGC counts.** Prediction accuracy using (**A**) full-length and (**B**) V3–V4 region 16S rRNA sequences, shown as the proportion of genomes within different prediction error ranges. Accuracy stratified by sequence identity to reference dataset: (**C**) 95–100 %, (**D**) 90–95 %, (**E**) 80 %–90 %, and (**F**) 0–80 %.The number of successful predictions for each method is indicated in the top-right corner of each panel.

To evaluate the accuracy of PSMPA in predicting the abundance of BGCs, we evaluated the pipeline on an independent test dataset comprising 5000 matched 16S rRNA-genome pairs, of which 4822 sequences containing the V3–V4 hypervariable region. To ensure the validity of the evaluation, we confirmed that the taxonomic distribution of the test dataset mirrors that of the reference dataset. Despite the inherent unevenness in microbial diversity, the absence of systematic bias ensures that the evaluation results are meaningful and representative (Fig. S3). Both full-length and partial (V3–V4) 16S rRNA sequences were analyzed. The psmpa2 approach consistently surpassed psmpa1 across all input types, with >80 % of psmpa2 predictions showing ≤1 BGC count deviation from antiSMASH benchmarks (Fig. 5A and B). The psmpa2 maintained stable performance regardless of statistical method (mean_float, mean_int, median_float, median_int), while psmpa1 exhibited higher sensitivity to Hidden State Prediction (HSP) algorithm selection, where the mp method outperformed other options (emp_prob, pic, scp, subtree_average) though remained inferior to psmpa2. These results indicate that the V3–V4 region is sufficient for reliable prediction of microbial secondary metabolic potential. Additionally, psmpa2 demonstrated robust prediction success, with 0–1 sequence failing across datasets, compared to 4–12 failures using psmpa1. This may be due to the more comprehensive reference dataset used by psmpa2. Extended evaluation of eight major BGC classes (PKSI, PKSother, NRPS, PKS-NRPS Hybrids, RiPPs, Saccharides, Terpenes, Others) using V3–V4 inputs confirmed high prediction accuracy for both methods (Fig. S4).

To evaluate how sequence novelty affects prediction accuracy, we stratified 16S rRNA sequences by their identity to reference sequences into four tiers: 95–100 %, 90–95 %, 80–90 %, and <80 %. An inverse correlation between sequence novelty and prediction accuracy is evident across the tested datasets (Fig. 5C–F). While psmpa2 achieved optimal performance for high-identity sequences (>95 %), psmpa1 with mp or emp_prob HSP algorithms demonstrated superior accuracy for novel sequences (<90 % identity), suggesting enhanced robustness for characterizing unclassified taxa. This observed trend requires validation through larger-scale studies. Additionally, to evaluate PSMPA's resolution at the inter-subspecies level, we clustered 5000 16S rRNA sequences from the test dataset using CD-HIT (99 % identity), resulting in 279 non-singleton clusters (4109 sequences). We selected the six largest clusters (covering ∼50 % of the test dataset) for further analysis. Within each cluster, we assessed the accuracy of PSMPA-predicted BGC abundance and found high consistency with reference values, with accuracy exceeding 80 % in all cases and reaching 100 % in some. Notably, variation in total BGC counts was observed even within these inter-subspecies clusters, highlighting PSMPA's ability to resolve biosynthetic differences among closely related strains (Fig. S5). Collectively, our results establish PSMPA as a reliable tool for estimating secondary metabolic potential from 16S rRNA data, with accuracy strongly correlated to reference dataset coverage. These findings provide practical guidelines for parameter selection based on expected microbial novelty in target samples.

### 3.4 Identification of biosynthetically rich strains from marine microbiomes using PSMPA

**Fig. 6:**
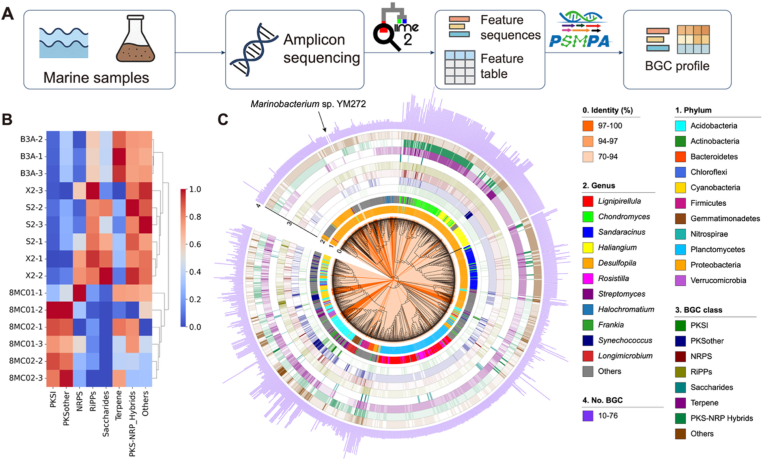
**Comparative analysis of BGC profiles across marine-derived samples and identification of biosynthetically rich strains using PSMPA.** (**A**) Workflow of 16S rRNA amplicon sequencing data analysis with PSMPA to predict BGC abundance. (**B**) Clustering heatmap of BGC profiles predicted by PSMPA across five sampling sites: 8MC01 and 8MC02 (seawater); B3A (mangrove rhizosphere sediment); X2 and S2 (intertidal flat sediment), with three technical replicates per site. (**C**) Phylogenetic tree and BGC profiles of candidate strains with ≥10 predicted BGCs isolated from the B3A mangrove sediment sample.

To guide the selection of biosynthetically rich strains for natural product discovery, we applied PSMPA to five marine environmental samples, including seawater (8MC01 and 8MC02), mangrove sediment (B3A), and intertidal flat sediment (X2 and S2). Based on 16S rRNA gene amplicon data, PSMPA predicted distinct BGC class abundance profiles across these samples. (Fig. 6A). The relative abundance of BGC classes was visualized through a clustering heatmap (Fig. 6B), revealing distinct BGC composition patterns across sampling sites. Notably, 8MC01 and 8MC02 were enriched in PKSI and PKSother BGCs; B3A was dominated by terpene BGCs and also showed relatively high levels of PKS-NRPS hybrids and other classes; while X2 and S2 were rich in RiPPs, PKS-NRPS hybrids, and other classes. These results demonstrate source-specific BGC distribution patterns. We focused on candidates with low 16S rRNA identity to known species but high BGC richness, favoring underexplored bacteria with potential for novel compound discovery. Strains from the B3A sample predicted to harbor ≥10 BGCs were prioritized for isolation (Fig. 6C). Guided by this result, we used tailored culture media, successfully isolating *Marinobacterium* sp. YM272 (97.71 % 16S rRNA identity to *M. mangrovicola*).

Whole-genome sequencing of YM272 yielded a 4.47 Mb single-contig genome (Fig. S6), and CheckM (v1.2.3) [28] analysis indicated 100 % completeness with 0.57 % contamination, confirming high assembly quality. The YM272 genome harbors 10 BGCs identified by antiSMASH, closely matching the 12 BGC classes predicted by PSMPA. A phylogenetic tree of 21 *Marinobacterium* genomes shows BGC distribution across strains (Fig. S7, Table S1). Genome similarity analysis with fastANI (v1.34) [44] revealed YM272 exhibited the highest ANI (83.896 %) with *M. mangrovicola* DSM 27697 and the lowest ANI (77.798 %) with *M. rhizophilum* DSM 18822. These results indicate that YM272 is genetically distinct and may represent a novel species within the *Marinobacterium* genus.

### 3.5 Discovery of new sulfur-containing natural products from *Marinobacterium* sp. YM272

**Fig. 7:**
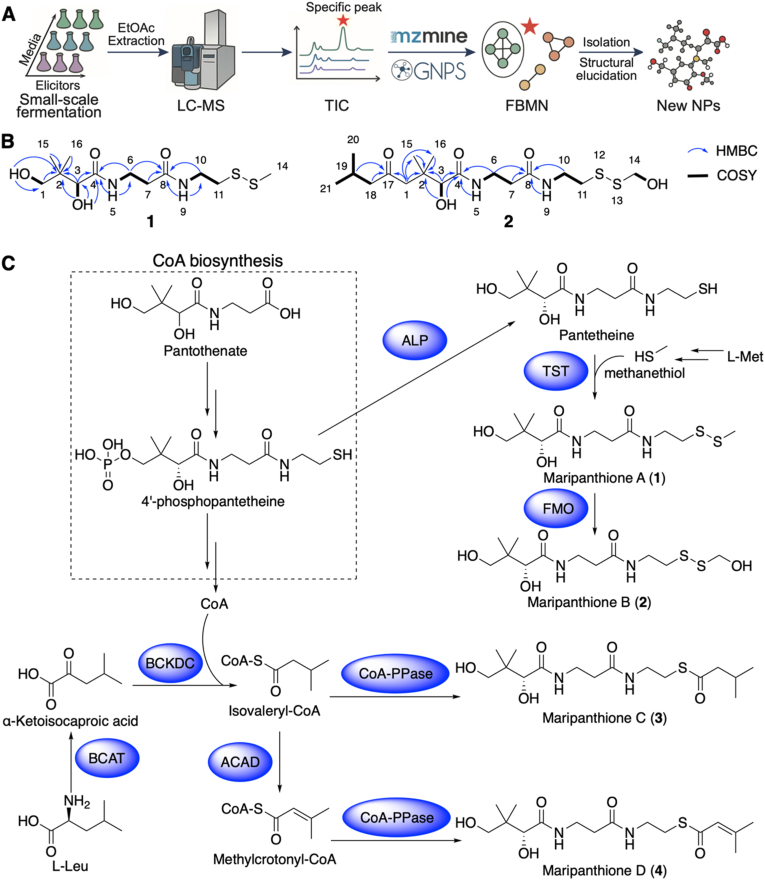
**Discovery workflow, structures, and putative biosynthetic pathway of maripanthiones. (**A**)***Marinobacterium* sp. YM272 was cultured under various combinations of media and elicitors to find specific peaks via LC-MS, which were then served as targets for guided isolation and purification. (**B**) COSY and key HMBC correlations of compounds **1** and **2**. (**C**) Putative biosynthetic pathway of maripanthiones. BCAT: branched-chain amino acid aminotransferase (WP_432697219.1); BCKDC: branched-chain α-ketoacid dehydrogenase complex (WP_432695507.1, WP_432695508.1, WP_432695509.1); ACAD: acyl-CoA dehydrogenase (WP_432697346.1); CoA-PPase: CoA pyrophosphatase (WP_432697441.1); ALP: alkaline phosphatase (WP_432697918.1); TST: sulfurtransferase (WP_432696195.1); FMO: flavin-containing monooxygenase (WP_432696426.1).

To explore the secondary metabolic potential of *Marinobacterium* sp. YM272, we performed a screening of culture media and chemical elicitors (Fig. 7A). Specifically, four different culture media (M1–M4) in combination with six chemical elicitors (A–F), resulting in 24 distinct conditions. Additional conditions included fermentations without inducers (G) and sterile medium blanks (H) for baseline comparison. Non-targeted metabolomics was performed by LC-MS/MS. Raw data were processed using MZmine 3 for feature extraction, followed by clustering and annotation through FBMN (Fig. S8). Given the high dimensionality of the mass feature data, principal component analysis (PCA) was applied to normalized feature intensities, revealing distinct clustering of conditions (Fig. S9). Notably, conditions 1A and 1B showed clear separation, and manual inspection of TIC/UV chromatograms indicated significantly enhanced feature diversity and intensity in 1B compared to controls. Consequently, condition 1B was prioritized for target compound production.

A 40-liter large-scale fermentation under condition 1B was carried out, and LC-MS-guided fractionation enabled the isolation of target compounds with *m/z* values of 347, 431, 385 and 383. This led to the discovery of four new sulfur-containing natural products, maripanthiones A (**1**, 7.3 mg), B (**2**, 1.0 mg) C (**3**, 3.2 mg) and D (**4**, 1.5 mg) (Fig. 7C), with **1**–**2** containing disulfide bonds and **3**–**4** featuring thioester bonds.

### 3.6 Structure elucidation of maripanthiones

Compound **1** was obtained as colorless oil. HR-ESI-MS/MS analysis established its molecular formula as C_12_H_24_N_2_O_4_S_2_, based on the observation of three pseudomolecular ion peaks at *m/z* 307.1135 [M-H_2_O + H]^+^ (calculated *m/z* 307.1150), 325.1246 [M+H]^+^ (calculated *m/z* 325.1256), and 347.1069 [M+Na]^+^ (calculated *m/z* 347.1070) in the HR-ESI-MS spectrum (Fig. S12.1), indicating the presence of two degrees of unsaturation in its structure. The ^13^C NMR spectrum (Table S2, Fig. S12.4) exhibited signals for two carbonyl carbons (*δ_C_* 172.8 and 170.6), an oxygenated methine carbon (*δ_C_* 74.9), and an oxygenated methylene carbon (*δ_C_* 68.0). Additionally, the spectrum displayed a quaternary carbon (*δ*_C_ 39.0), four methylene carbons (*δ*_C_ 34.7, 35.0, 37.8 and 36.3), and three methyl carbons(*δ*_C_ 20.2, 20.8 and 22.6). The ^1^H NMR spectrum (Table S2, Fig. S12.3) of 1 shows the presence of two amide protons (*δ*_H_ 7.69 and 8.01) and two exchangeable protons (*δ*_H_ 4.49 and 5.38), suggesting two amide carbonyl carbons and two hydroxyl groups in the structure. The analysis of the ^1^H–^1^H COSY (Fig. S12.5) revealed two major proton spin systems: H-9/H-10/H-11 and H-5/H-6/H-7 subunits of structure **1** (Fig. 7B). HMBC correlations (Fig. S12.7) from H-6/H-7/H-9/H-10 to C-8, from H-3/H-5/H-6/3-OH to C4, from H-3 to C-1/C-2/C-4/C-15/C-16, from 1-OH to C-1/C-2, along with the analysis of chemical shifts, provided information on the fragment from C-1 to C-11 of proposed structure of **1** (Fig. 7B). It was given the trivial name maripanthione A. Considering the same biosynthetic origin and structural similarities, the absolute configuration of maripanthione A was determined by comparing its optical rotation with that of the known compound maripanthione C [45], and was assigned as 3*R*.

Compound **2** was obtained as colorless oil. The HR-ESI-MS/MS spectrum (Fig. S13.1) showed two positive pseudomolecular ion peaks at *m/z* 409.1812 [M+H]^+^ (calculated *m/z* 409.1831) and 431.1635 [M+Na]^+^ (calculated *m/z* 431.1645), indicating three degrees of unsaturation and a molecular formula of C_17_H_32_N_2_O_5_S_2_. The UV absorption and ^1^H/^13^C NMR data (Table S2, Fig. S13.3-S13.4) of **2** were consistent with those of **1**. Detailed analysis of the 1D/2D NMR data (Fig. S13.3-S13.7) of **2** revealed that it was similar to **1**, except for the additional 3-methylbutanal fragment, established by the COSY spin system H-18/H-19/H-20/H-21 and HMBC correlation from H-19/H1-18 to C-17. Additionally, an exchangeable proton was linked to C-14, instead of C-1, unlike in structure **1**. The linkage between the additional fragment and the rest of the structure was established by an HMBC correlation from H-1 to C-17. Other key correlations observed in the HMBC spectrum of **2** (Fig. 7B, Fig. S13.7) contributed significantly to the confirmation of its structure. The optical rotation of **2** was similar to those of **1**, indicating that **2** shares the same configuration, 3*R*, based on the same biosynthetic origin.

Compounds **3** and **4** were characterized as maripanthione C and D (reference) based on the interpretation of HR-ESI-MS/MS (Fig. S14.1, S15.1), UV (Fig. S14.2, S15.2), and 1D/2D NMR spectroscopic data (Fig. S14.3-S14.7, S15.3-S15.7, Table S3). These compounds represent newly discovered natural products. All detailed structural characterization data (MS, UV, NMR, and IR) of maripanthiones can be found in the Supplementary Information (Fig. S10–S15, Table S2–S3&S5–S8).

### 3.7 Putative biosynthetic pathway of maripanthiones

To explore the biosynthetic origin of maripanthiones, we mined the genome of *Marinobacterium* sp. YM272. Our analysis revealed that the key enzymes are encoded by dispersed genomic loci rather than a conventional, clustered BGC as defined by antiSMASH. Consequently, we proposed a biosynthetic pathway by intergrating structural analysis, genomic annotation, and established metabolic routes (Fig. 7C). Compounds **1** and **2** are likely derived from pantetheine, which can be generated from the CoA biosynthesis intermediate 4′-phosphopantetheine via alkaline phosphatase (ALP)-mediated dephosphorylation. We propose that pantetheine undergoes sulfur incorporation through a sulfurtransferase (TST), forming a disulfide bond using methanethiol as the thiol donor, potentially derived from l-methionine. Subsequent oxidation of the terminal methylthio group by a flavin-containing monooxygenase (FMO) may yield the hydroxylated disulfide in compound **2**. Compounds **3** and **4** likely originate from l-leucine catabolism. Transamination of l-leucine by branched-chain amino acid aminotransferase (BCAT), followed by decarboxylation via the branched-chain α-ketoacid dehydrogenase complex (BCKDC) generates isovaleryl-CoA, which is further converted to methylcrotonyl-CoA by acyl-CoA dehydrogenase (ACAD). These CoA thioesters may then undergo hydrolysis by CoA pyrophosphatase (CoA-PPase) releasing **3** and **4**. These related enzymes are not organized in a contiguous gene cluster, suggesting a non-canonical or dispersed biosynthetic route. The lack of a clearly defined BGC highlights the likelihood that maripanthiones are formed through shunt pathways branching from primary metabolism rather than a dedicated secondary metabolite gene cluster. Nonetheless, additional experimental evidence is needed to verify this biosynthetic proposal.

## 4 Discussion

In this study, we developed PSMPA to predict microbial secondary metabolic potential based on 16S rRNA sequences. It should be clarified that PSMPA predictions are not based on causation; rather, the choice of 16S rRNA as a marker gene relies on the observed correlation between phylogeny and biosynthetic capacity, particularly in high-quality genomes. Therefore, this correlation-based approach has inherent limitations. Firstly, when applied to microbiome samples containing many novel taxa, the absence of corresponding reference genomes in the dataset may reduce the prediction accuracy. Nevertheless, such taxa often carry significant research value and interest, especially when PSMPA predictions suggest relatively high secondary metabolic potential, which at least indicates that their known close relatives possess notable biosynthetic capacity. Secondly, horizontal gene transfer (HGT), convergent evolution, and genome plasticity can result in varying BGC profiles even among closely related strains [[46], [47], [48]]. For example, in our bacterial BGC atlas, we find seven *Salinispora arenicola* genomes sharing identical 16S rRNA sequences, yet their total BGC counts ranged from 26 to 35 (Fig. S16). This observation is consistent with previous studies highlighting the extensive influence of HGT on the *Salinispora* genus [48]. Additionally, variation in genome assembly quality can impact the number of predicted BGCs, leading to potential biases in the reference dataset and, consequently, influencing the accuracy of PSMPA predictions. To mitigate this factor, we carefully curated high-quality genomes (≥90 % completeness, ≤5 % contamination) as the PSMPA reference dataset. Therefore, PSMPA serves as a useful first-pass estimator but should be complemented by genome-resolved analyses for high-resolution predictions.

PSMPA is the first tool to predict BGC profiles from marker genes such as 16S rRNA genes. Compared to tools like BiG-MAP [49], which assess BGC abundance and expression by aligning reference BGC sequences to metagenomic and metatranscriptomic data—an approach that requires high sequencing cost and greater computational resources—PSMPA offers a cost-effective alternative based on amplicon sequence inference. By leveraging this advantage, PSMPA is suitable for early-stage screening of environmental microbiota to identify promising bacterial taxa for downstream natural product discovery, particularly when only amplicon data are available. While BiG-MAP provides more accurate profiling of BGC abundance and expression, it is better suited for mechanistic studies because shotgun metagenomic sequencing often gives a more detailed snapshot than 16S rRNA sequencing [50].

The discovery of *Marinobacterium* sp. YM272 highlights the effectiveness of PSMPA-guided strain prioritization. However, even with genus-targeted strategies and optimized cultivation conditions, traditional plate-based isolation remains a stochastic process. In particular, the selective isolation of uncultivable or poorly studied taxa remains a major obstacle. Targeted single-cell microbial isolation techniques, such as Live-FISH, have been shown to recover viable bacteria by hybridizing taxonomy-specific 16S probes followed by fluorescence-activated cell sorting (FACS) [51]. Additionally, microfluidic droplet systems enable ultrahigh-throughput encapsulation and functional screening of individual cells, offering a powerful platform for phenotype-based selection and isolation of target microbes [52,53]. Future integration of PSMPA with 16S rRNA probe-guided live-FISH coupled to FACS or microfluidic droplet-based single-cell sorting could further improve targeted recovery of biosynthetically rich strains.

The newly discovered sulfur-containing maripanthiones A–D, characterized by thioester and disulfide bonds, underscore the untapped biosynthetic diversity within underexplored marine genera. Further studies on their bioactivities and ecological functions could uncover novel modes of microbial interaction or lead compounds for therapeutic development.

## 5 Conclusions

In conclusion, this study establishes PSMPA as a scalable, cost-effective tool for secondary metabolic potential prediction of microbiome. By integrating PSMPA-driven strain selection with metabolomics-guided prioritization, the approach enables rapid early-stage identification of promising strains and novel compounds. When combined with single-cell sorting and advanced cultivation technologies, PSMPA holds great promise for accelerating the discovery of novel antibiotics and rare biosynthetic strains, with broad potential for exploring microbial chemodiversity across both environmental and biomedical research domains.

## CRediT authorship contribution statement

**Zhen-Yi Zhou:** Writing – original draft, Visualization, Software, Methodology, Investigation, Data curation. **Qun-Jian Yin:** Resources, Formal analysis, Data curation. **Buddha Bahadur Basnet:** Writing – review & editing, Methodology. **Zi-Yang Li:** Validation, Formal analysis. **Gen Li:** Visualization, Data curation. **Mahmoud Emam:** Resources. **Qi-Hao Wu:** Resources. **Hong Wang:** Supervision, Funding acquisition. **Bin Wei:** Writing – review & editing, Supervision, Project administration, Funding acquisition, Conceptualization.

## Consent for publication

All authors have read and approved the final manuscript and consent to its publication.

## Availability of data and materials

All data supporting the findings of this study are available through public repositories or included in the Supplementary Information. The source code for the PSMPA pipeline, including the core package and scripts used for data analysis, is publicly available at GitHub https://github.com/BioGavin/psmpa. The complete genome sequence of *Marinobacterium* sp. YM272 is publicly available in GenBank (accession number CP194171), and the 16S rRNA amplicon sequencing data from five marine samples have been deposited in the NCBI SRA under BioProject PRJNA1276294. Additional datasets are provided in the Supplementary Information.

## Funding

This work was supported by the National Key Research and Development Programs (nos. 2022YFC2804700 and 2022YFC2804104), the General Program of National Natural Science Foundation of China (no. 42276137), and the international (regional) cooperation and exchange program jointly initiated by the National Natural Science Foundation of China (no. W2412100).

## Declaration of competing interest

The authors declare that they have no known competing financial interests or personal relationships that could have appeared to influence the work reported in this paper.
