# Supplementary material for: Amplicon-based prediction of secondary metabolic potential of microbiomes facilitates natural product discovery
Source: Synth Syst Biotechnol. 2025 Nov 4;11:385–96. doi: 10.1016/j.synbio.2025.10.012 (PMC12637254; doi:10.1016/j.synbio.2025.10.012)
Supplement: Multimedia component 2 [file mmc2.docx]

**Supporting Information**

Supplementary Text

# 16S rRNA gene and BGC database processing for psmpa1

The PSMPA pipeline provides two classes of methods to predict the BGC profiles of microbiomes using 16S rRNA gene sequences. The first one (psmpa1) uses the same algorithm provided by PICRUSt2, which contains a reference tree consisting of 20,000 unique full-length 16S rRNA genes [1]. The corresponding bacterial genomes of these 16S rRNA sequences were collected according to the IMG genome ids provided in<https://github.com/gavinmdouglas/picrust2_manuscript>. Notably, a few genomes were replaced by those with the same lineage because they were not available in the IMG or NCBI database. All acquired genome sequences were analyzed by antiSMASH (v5.2.0) [2], and the numbers of each class of BGCs in each genome were extracted using a custom python script to prepare the traits of BGCs for all the 20,000 full-length 16S rRNA genes in the database.

# 16S rRNA gene and BGC database processing for psmpa2

The second algorithm (psmpa2) uses BLASTn to predict the BGC profiles for each input sequence by comparing the sequence identity against a 16S rRNA gene database. The psmpa2 collected all bacterial genomes (220,524 in total) from NCBI RefSeq Assembly Database (<https://ftp.ncbi.nlm.nih.gov/genomes/refseq>). After the filtering steps described in the main text, a total of 53,812 entries of 16S rRNA gene sequences were curated in the psmpa2 database. Then, genomes lacking 16S rRNA gene or complete taxonomic lineage were removed and the remaining 175,321 genomes were analyzed by antiSMASH (v5.2.0). The numbers of each class of BGCs were extracted as described above. Since some genomes with identical 16S rRNA sequences may contain a different composition of BGCs, we used four different methods (*mean_float, mean_int, median_float, median_int*) to prepare the BGC database for all the 53,812 entries in 16S rRNA gene database. As the names of these methods indicate, the number of each class of BGCs for each entry were obtained by calculating the mean or median number (retaining floats or integers) of BGCs in strains with the identical 16S rRNA genes.

# Classification of BGCs in PSMPA

To streamline the predictive outcomes, we opted to employ the eight BGC classes (type I polyketide synthases (PKSs), other PKSs, nonribosomal peptide synthetases (NRPSs), PKS/NRPS hybrids, ribosomally synthesized and posttranslationally modified peptides (RiPPs), saccharides, terpenes and others) utilized in BiG-SCAPE [3], rather than the extensive array of BGC classes used in antiSMASH. For this, we located the function within the source code of BiG-SCAPE (v1.1.5) responsible for mapping antiSMASH classes to BiG-SCAPE classes. Subsequently, we wrote a custom Python script (<https://github.com/BioGavin/astool/blob/main/script/others/as2bs.py>) based on the conversion rules provided by this function to perform the conversion of BGC classes. It is noteworthy to mention that during the conversion process, we encountered an antiSMASH class, lanthidin, which could not be classified by the BiG-SCAPE classification function. Through comprehensive literature review, we assigned it to the RiPPs class and integrated this classification rule into the aforementioned custom script.

# Culture medium and elicitors

M1: glucose 0.01%, peptone 0.5% and yeast extract 0.1% in artificial seawater.

M2: glucose 0.05%, sodium pyruvate 0.03%, soluble starch 0.05%, peptone 0.05%, yeast extract 0.05%, tryptone 0.05%, MgSO_4_·7H_2_O 0.005%, K_2_HPO_4_ 0.03% in artificial seawater.

M3: soluble starch 1%, peptone 0.03%, MgSO_4_·7H_2_O 0.005%, NaNO_3_ 0.2%, KCl 0.2%, K_2_HPO_4_ 0.2%, CaCO_3_ 0.002%, FeSO4 0.001% in artificial seawater.

M4: glucose 0.2%, soluble starch 2.5%, peptone 0.5%, casein 0.5%, yeast extract 0.5%, CaCO_3_ 0.3% in artificial seawater.

Elicitors reported to activate gene cluster expression were used in this study [4,5]:

A: LaCl_3_·7H_2_O 2 mM

B: NiCl_2_·6H_2_O 100 µM

C: EDTA 10 mM

D: DMSO 3%

E: Streptomycin 100 mg/L

F: Kanamycin 10 mg/L

# General experimental instrumentation and conditions

Analytical HPLC was conducted on a Waters e2695 system with a 2489 UV/Vis detector using a Shimadzu ShimNex CS C18 column (5 μm, 4.6 × 100 mm). A 10–100% MeOH gradient was applied at 1.0 mL/min, with the column maintained at 25 °C. Detection wavelengths were 210 and 254 nm, and 10 μL samples were analyzed in per injection.

Semi-preparative purification was performed on a Shimadzu LC-16 system with an SPD-16 detector and manual injector, using an Agilent Eclipse XDB-C18 column (5 μm, 9.4 × 250 mm). Isocratic or gradient elution with MeOH or ACN in water containing 0.001% TFA was used to purify target compounds.

UPLC-HR-ESI-MS/MS analysis was performed on a SCIEX X500B QTOF system using an Agilent SB-C18 column (1.8 μm, 2.1 × 100 mm) with a binary gradient of H_2_O (A) and MeOH (B) at 0.3 mL/min and 35 °C. The gradient started at 10% B, held for 2 min, ramped to 100% over 18 min, and was maintained at 100% B for 5 min. Data were acquired in positive mode using an Information Dependent Acquisition (IDA) method. Ionization was set at +5500 V and 500 °C. TOF MS ranged from 100–1000 Da (declustering potential: 80 V, collision energy: 10 V), and MS/MS from 50–1000 Da (collision energy: 35 V, CE spread: 15 V).

Nuclear magnetic resonance (NMR) spectra were recorded on a 500 or 600 MHz NMR spectrometer (Bruker BioSpin GmbH, Germany), with all samples dissolved in DMSO-*d_6_*.

LC-MS/MS data were processed in MZmine3 after converting WIFF2 files to mzML using msconvert. The UPLC-QTOF-DDA workflow in the mzwizard module was run in batch mode with default settings, adjusting only MS1 and MS2 noise thresholds, to generate quantification tables and MGF files. These were submitted to GNPS for Feature-Based Molecular Networking (FBMN) using default parameters.

Optical rotation was measured at 25 °C using a Rudolph Research Analytical Autopol I polarimeter (Rudolph Research Analytical, USA) with a 1 dm cell at a wavelength of 589 nm, using MeOH as the solvent.

The infrared absorption spectrum was recorded on a NICOLET iS50 FT-IR spectrometer (Thermo Scientific, USA) equipped with an ATR (attenuated total reflectance) accessory. The sample was dissolved in MeOH and applied directly onto the ATR crystal. The spectrum was acquired with 16 scans under standard conditions.

# FBMN Analysis Details

In this study, MZmine3 was employed to process the mass spectrometry data, yielding 343 distinct mass spectrometric features. These features were subsequently uploaded to the Global Natural Products Social Molecular Networking (GNPS) platform for Feature-Based Molecular Networking (FBMN) analysis. The resulting molecular network comprised 40 clusters and 153 singleton nodes. Among these, 21 features received annotation information. After manual verification, annotations inconsistent with the predicted adduct forms in the Ion Identity Molecular Networking (IIMN) analysis were excluded. Ultimately, the annotation results of 18 nodes are shown in **Figure S8** and details are in **Table S4**.

#

# Information of maripanthiones

Maripanthione A (**1**): colorless oil; [α]^25^_D_ +25 (c 0.18, MeOH); UV (MeOH) λ_max_ 228 nm; ^1^H NMR (500 MHz, DMSO-*d_6_*) (carbon labeling shown in Figure S12.3) *δ* 0.77 (s, H_3_-15), 0.79 (s, H_3_-16), 2.26 (td, *J* = 7.05, 2.67 Hz, H_2_-7), 2.40 (s, H_3_-14), 2.76 (t, *J* = 6.87 Hz, H_2_-11), 3.16 (o, H-1a), 3.23 (o, H-6a), 3.28 (o, H-1b), 3.32 (o, H-6b and H_2_-10), 3.69 (d, *J* = 5.41 Hz, H-3), 4.49 (t, *J* = 5.60 Hz, 1-OH), 5.38 (d, *J* = 5.38 Hz, 3-OH), 7.69 (t, *J* = 5.96 Hz, 5-NH), 8.10 (t, *J* = 5.64 Hz, 9-NH); ^13^C NMR (125 MHz, DMSO-*d_6_*) *δ* 20.2 (C-15), 20.8 (C-16), 22.6 (C-14), 34.7 (C-6), 35.0 (C-7), 36.3 (C-11), 37.8 (C-10), 39.0 (C-2), 68.0 (C-1), 74.9 (C-3), 170.6 (C-8), 172.8 (C-4); HR-ESI-MS for the [M+Na]^+^ ion at *m/z* 347.1069 (calculated [M+Na]^+^ ion for C_12_H_24_N_2_O_4_S_2_ at *m/z* 347.1070, PPM = -0.20)

Maripanthione B (**2**): colorless oil; [α]^25^_D_ +6 (c 0.033, MeOH); UV (MeOH) λ_max_ 227 nm; ^1^H NMR (600 MHz, DMSO-*d_6_*) (carbon labeling shown in Figure S13.3) *δ* 0.86 (s, H_3_-16), 0.87 (s, H_3_-15), 0.90 (s, H_3_-21), 0.91 (s, H_3_-20), 1.99 (dp, *J* = 13.67, 6.85 Hz, H-19), 2.18 (d, *J* = 6.77 Hz, H_2_-18), 2.27 (td, *J* = 7.10, 2.79 Hz, H_2_-7), 2.77 (t, *J* = 6.87 Hz, H_2_-11), 3.17 (d, *J* = 5.25 Hz, H_2_-14), 3.23 (o, H-6a), 3.31 (o, H-6b), 3.32 (o, H_2_-10), 3.68 (d, *J* = 5.72 Hz, H-3), 3.85 (d, *J* = 10.54 Hz, H-1a), 3.91 (d, *J* = 10.54 Hz, H-1b), 4.09 (m, 14-OH), 5.62 (d, *J* = 5.71 Hz, 3-OH), 7.73 (t, *J* = 5.91 Hz, 5-NH), 8.08 (t, *J* = 5.68 Hz, 9-NH); ^13^C NMR (150 MHz, DMSO-*d_6_*) *δ* 20.3 (C-16), 20.8 (C-15), 22.1 (C-20 and C-21), 25.1 (C-19), 34.7 (C-6), 35.0 (C-7), 36.3 (C-11), 37.6 (C-2), 37.8 (C-10), 42.7 (C-18), 48.5 (C-14), 69.4 (C-1), 74.2 (C-3), 170.5 (C-8), 171.8 (C-4), 172.1 (C-17); HR-ESI-MS for the [M+Na]^+^ ion at *m/z* 431.1635 (calculated [M+Na]^+^ ion for C_17_H_32_N_2_O_5_S_2_ at *m/z* 431.1645, PPM = -2.28)

Maripanthione C (**3**): colorless oil; [α]^25^_D_ +12.5 (c 0.088, MeOH); UV (MeOH) λ_max_ 234 nm; ^1^H NMR (500 MHz, DMSO-*d_6_*) (carbon labeling shown in Figure S14.3) *δ* 0.77 (s, H_3_-18), 0.79 (s, H_3_-17), 0.89 (d, *J* = 6.68 Hz, H_3_-15 and H_3_-16), 2.03 (m, H-14), 2.24 (td, *J* = 7.06, 2.61 Hz, H_2_-7), 2.45 (d, *J* = 7.10 Hz, H_2_-13), 2.89 (t, *J* = 6.90 Hz, H_2_-11), 3.17 (o, H-1a and H_2_-10), 3.22 (o, H-6a), 3.30 (o, H-1b and H-6b), 3.69 (d, *J* = 5.47 Hz, H-3), 4.47 (t, *J* = 5.59 Hz, 1-OH), 5.37 (d, *J* = 5.52 Hz, 3-OH), 7.68 (t, *J* = 5.92 Hz, 5-NH), 8.09 (t, *J* = 5.73 Hz, 9-NH); ^13^C NMR (125 MHz, DMSO-*d_6_*) *δ* 20.3 (C-18), 20.9 (C-17), 21.9 (C-15 and C-16), 26.0 (C-14), 28.0 (C-11), 34.7 (C-6), 35.1 (C-7), 38.2 (C-10), 39.0 (C-2), 52.1 (C-13), 68.0 (C-1), 75.0 (C-3), 170.6 (C-8), 172.8 (C-4), 198.0 (C-12); HR-ESI-MS for the [M+Na]^+^ ion at *m/z* 385.1771 (calculated [M+Na]^+^ ion for C_16_H_30_N_2_O_5_S at *m/z* 385.1768, PPM = 0.87)

Maripanthione D (**4**): colorless oil; [α]^25^_D_ +22.9 (c 0.048, MeOH); UV (MeOH) λ_max_ 240, 265 nm; ^1^H NMR (500 MHz, DMSO-*d_6_*) (carbon labeling shown in Figure S15.3) *δ* 0.77 (s, H_3_-18), 0.79 (s, H_3_-17), 1.87 (d, *J* = 1.34 Hz, H_3_-16), 2.10 (d, *J* = 1.30 Hz, H_3_-15), 2.25 (td, *J* = 7.20, 3.14 Hz, H_2_-7), 2.91 (t, *J* = 6.85 Hz, H_2_-11), 3.17 (o, H-1a and H_2_-10), 3.21 (o, H-6a), 3.29 (o, H-1b and H-6b), 3.69 (d, *J* = 4.60 Hz, H-3), 4.49 (t, *J* = 5.41 Hz, 1-OH), 5.40 (d, *J* = 5.51 Hz, 3-OH), 6.06 (p, *J* = 1.25 Hz, H-13), 7.69 (br t, *J* = 5.85 Hz, 5-NH), 8.11 (br t, *J* = 5.65 Hz, 9-NH); ^13^C NMR (125 MHz, DMSO-*d_6_*) *δ* 20.2 (C-18), 20.8 (C-15 and C-17), 26.5 (C-16), 27.8 (C-11), 34.7 (C-6), 35.0 (C-7), 38.2 (C-10), 38.9 (C-2), 68.0 (C-1), 74.9 (C-3), 122.6 (C-13), 154.6 (C-14), 170.5 (C-8), 172.8 (C-4), 187.8 (C-12); HR-ESI-MS for the [M+Na]^+^ ion at *m/z* 383.1601 (calculated [M+Na]^+^ ion for C_16_H_28_N_2_O_5_S at *m/z* 383.1611, PPM = -2.65)

# Reported information about maripanthiones

A SciFinder search revealed no matching structures for maripanthiones A (**1**) and B (**2**), indicating that they are new compounds. In contrast, maripanthione C (**3**) was found in four patents, and maripanthione D (**4**) was reported in both a journal article and a patent; however, in all cases, they were described only as chemically synthesized products. Therefore, the isolation of **3** and **4** from natural sources represents their first identification as natural products.

For maripanthione C, three patents (US10376453, EP3549574, KR1695375) from the Korea Institute of Ocean Science and Technology report identical findings on its activity in promoting hair growth and preventing hair loss. Experiments have shown that this pantetheine derivative significantly promotes the proliferation of dermal papilla cells, with effects comparable to minoxidil. These patents also outline synthesis methods for this pantetheine derivative and provide the ^1^H NMR (500 MHz, MeOH-*d_4_*), ^13^C NMR and HMBC data. Additionally, another patent (CN118086234) from Shanghai Jiao Tong University reports a ligase that catalyzes thioester formation using panthiol as a substrate, with **3** formed via the condensation of panthiol with isovaleric acid under ligase action.

For maripanthione D, it was chemically synthesized as an intermediate for the target compound, with ^1^H NMR (400 MHz, MeOH-*d_4_*) data provided in the research paper [6]. The patent (WO2020113209) from Comet Therapeutics, Inc. (US) reports the synthesis steps, purification methods, and ^1^H NMR (400 MHz, CDCl_3_) data. Compound **4** enhances mitochondrial respiration and spare respiratory capacity by 10-50% in PA and MMA cell lines, reduces inflammatory cytokine secretion (IL-6, IL-10, TNFα) by at least 30% in M1 macrophages, and decreases ROS levels by 10-30% in models such as KSS and VLCFA. For further details, see **Table S9** below.

#

Supplementary Figures

## **
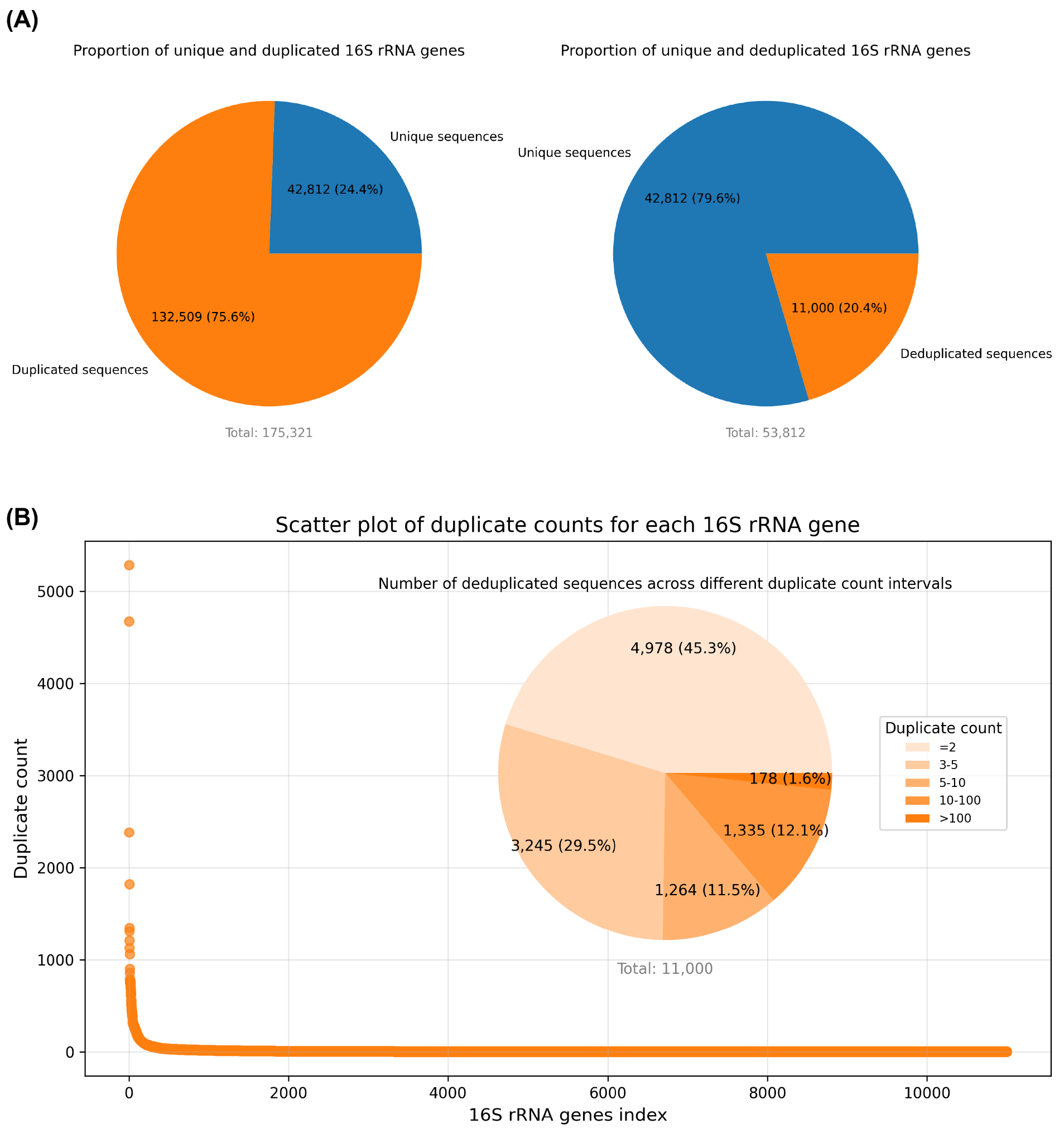
**

## **Figure S1** Analysis of the duplication of representative 16S rRNA genes of 216,408 genomes. (A) The pie charts illustrate the proportions of unique and duplicated sequences before (left) and after (right) deduplication. (B) The scatter plot shows the duplicate counts for each 16S rRNA gene, with the inset (top right) displaying the proportion of 16S rRNA genes across different duplicate count intervals.

##


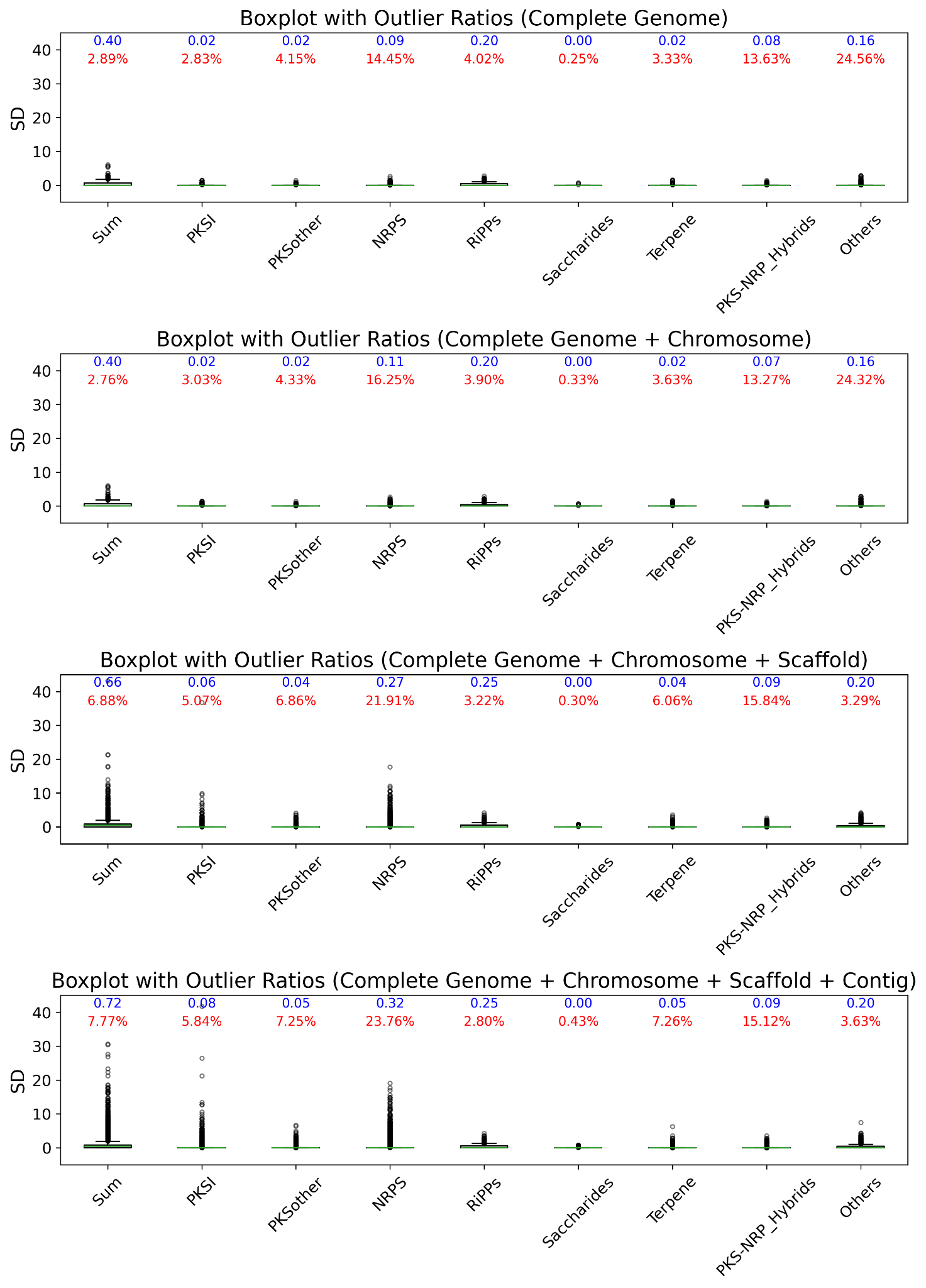


## **Figure S2** Boxplot of standard deviations (SD) for various classes of BGCs across genomes with the same 16S rRNA gene, categorized by different genome assembly levels. Mean values (blue) and outlier ratios (red) are labeled above the boxplot.


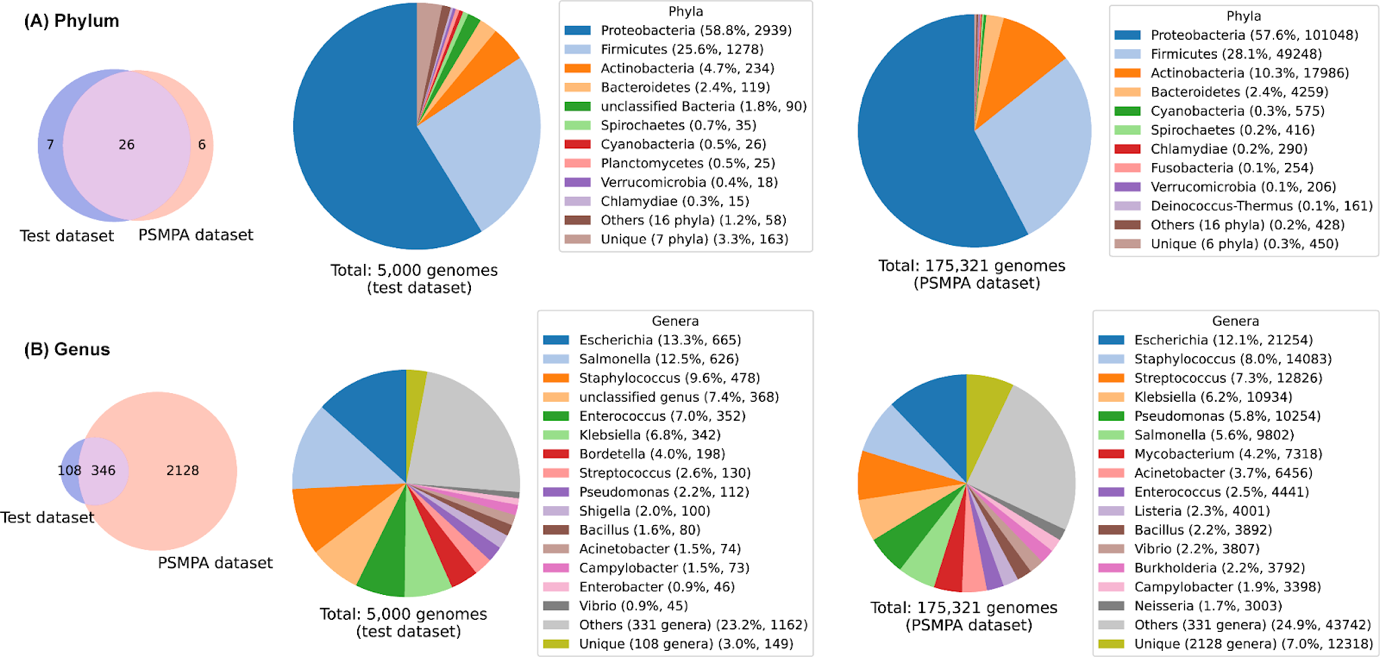


## **Figure S3** Taxonomic composition and overlap between the test dataset and the PSMPA dataset. (A) Phylum-level comparison. Left: Venn diagram showing common and unique phyla. Middle and right: Pie charts summarizing phylum composition of the two datasets. Top 10 common phyla are labeled; remaining common phyla are grouped as “Others” and dataset-specific phyla as “Unique”. (B) Genus-level comparison. Left: Venn diagram showing shared and unique genera. Middle and right: Pie charts showing genus composition of the two datasets. Top 15 genera are labeled; other common genera are grouped as “Others” and dataset-specific genera as “Unique”.

**
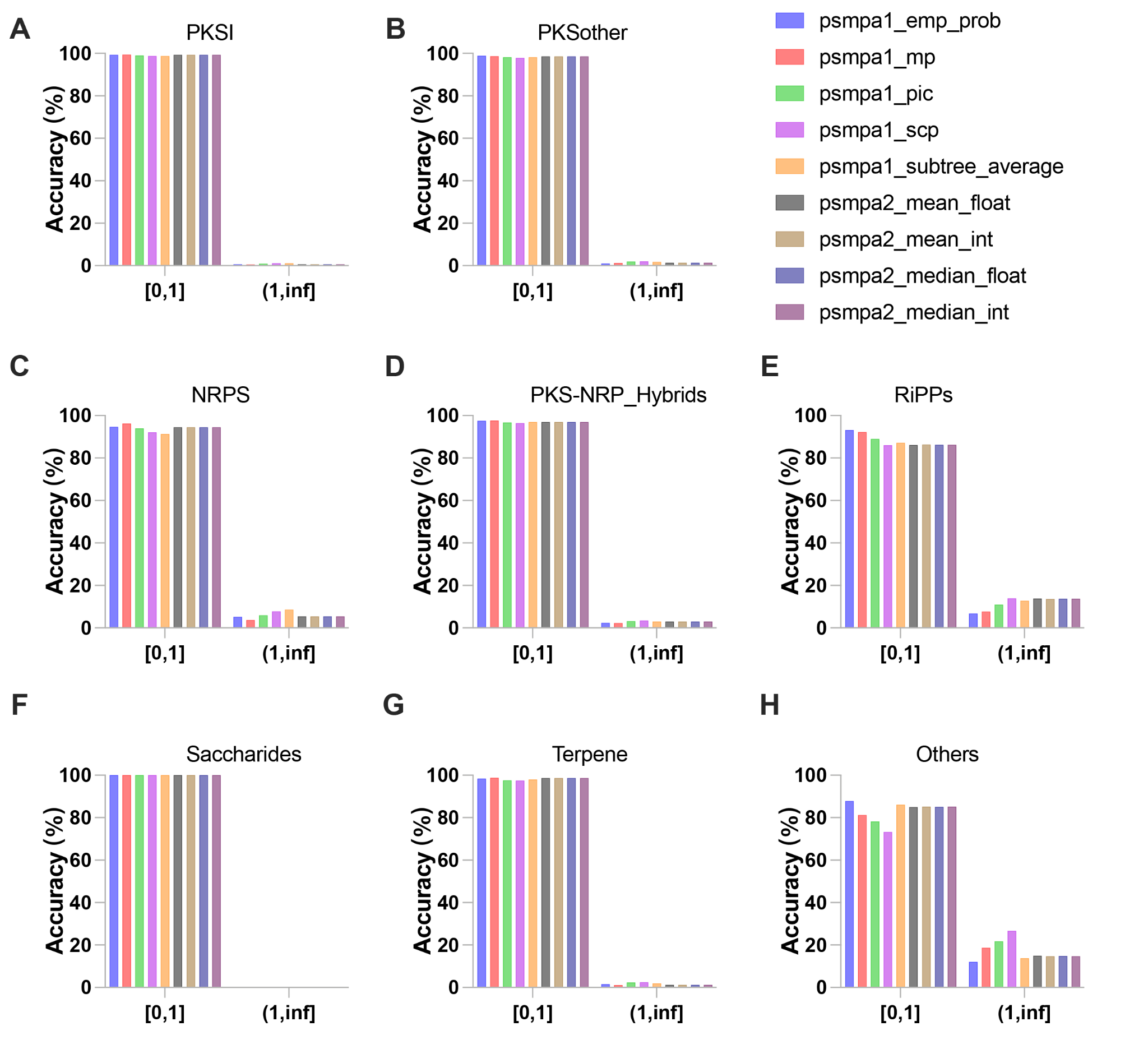
**

## **Figure S4** Accuracy of PSMPA under different parameters in the prediction of BGC counts in 5,000 bacterial genomes. Bias of the number of (**A**) NRPS, (**B**) T1PKS, (**C**) Terpene, (**D**) Bacteriocin, (**E**) Lanthipeptide, (**F**) T3PKS, (**G**) Siderophore, (**H**) Lanthidin, and (**I**) Lassopeptide BGCs using the V3-V4 region of 16S rRNA genes.


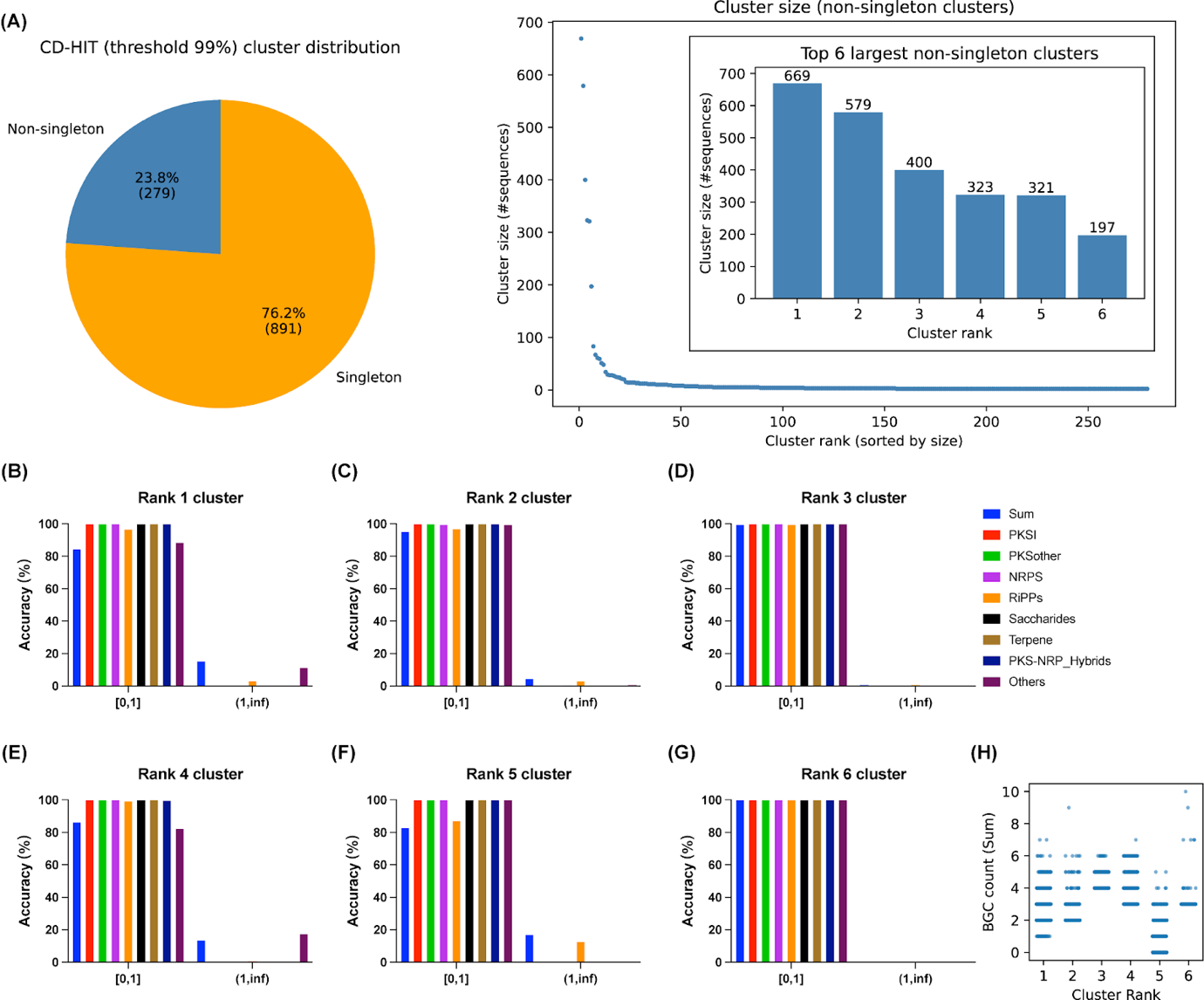


## **Figure S5** Accuracy of PSMPA-predicted BGC profile among subspecies (A) CD-HIT (99% identity threshold) clustering of sequences yielded 1170 clusters, of which 279 (23.8%) were non-singleton clusters. The cluster size distribution (right panel) shows a heavy-tailed pattern, and the top six largest non-singleton clusters were highlighted in the inset. (B–G) For each of the top 6 clusters, the prediction accuracy of BGC profiles was evaluated within two distance bins: [0, 1] and (1, ∞), based on the custom similarity metric. Accuracy was calculated for each BGC class separately, including the overall BGC sum. Higher accuracy in the [0, 1] bin indicates strong intra-cluster


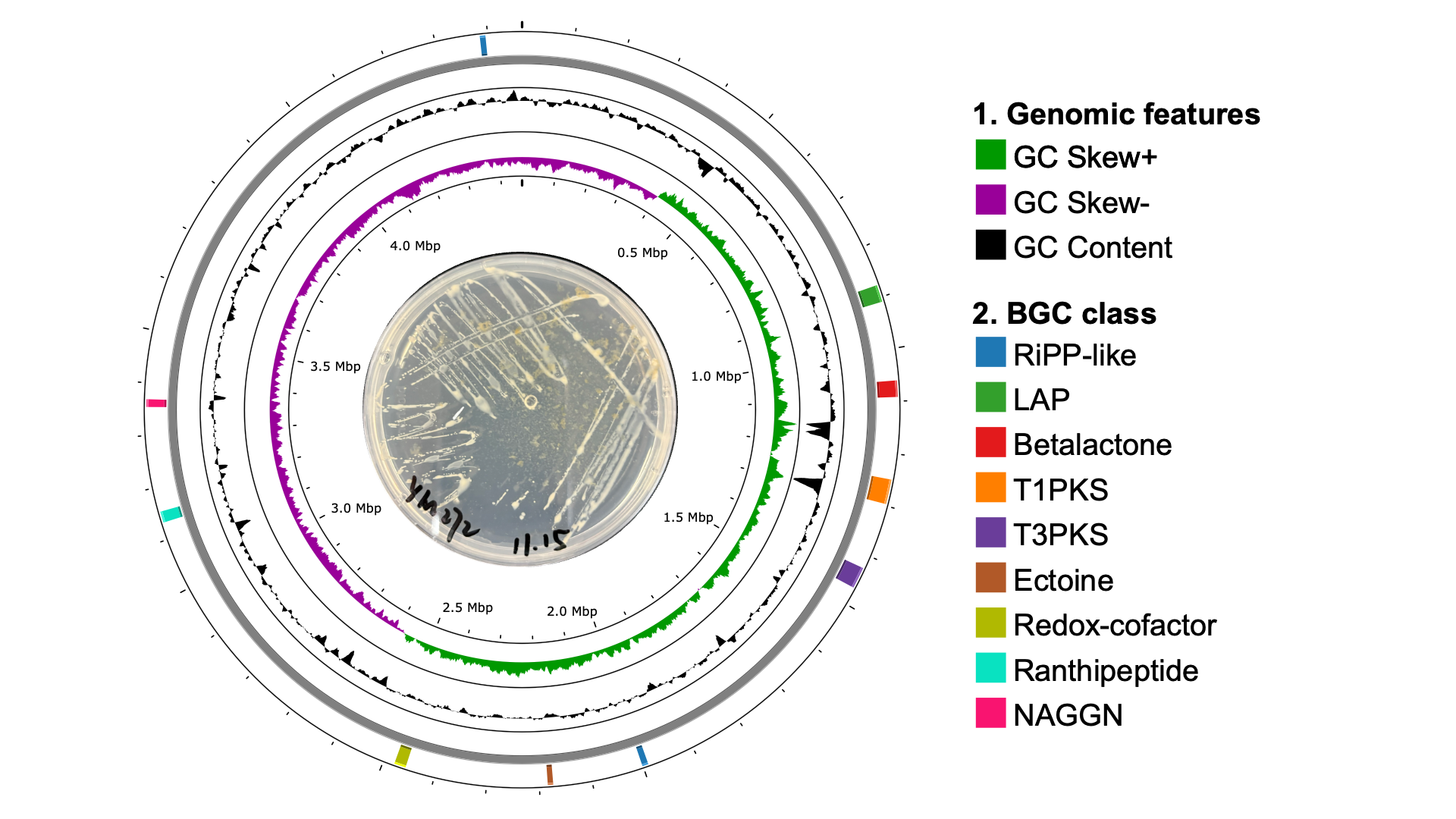


## **Figure S6** Genomic features and BGCs distribution of *Marinobacterium* sp. YM272

##

## **Figure S7** Phylogenetic tree of *Marinobacterium* genus constructed by UBCG (v3.0) and decorated by the number of BGCs.


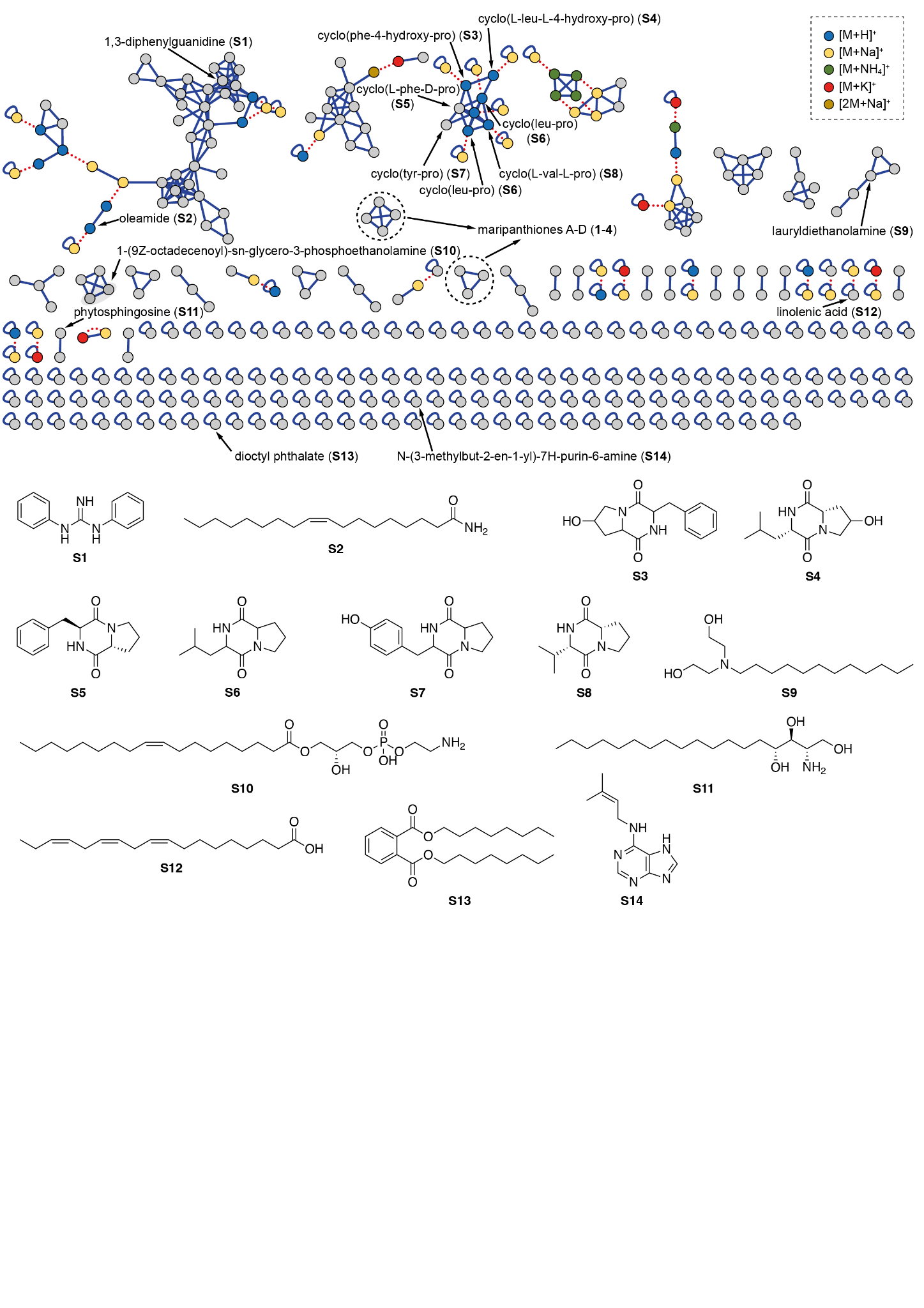


## **Figure S8** Molecular network (FBMN and IIMN) of crude extracts from *Marinobacterium* sp. YM272 in different culture conditions. More annotation details are shown in **Table S4**.


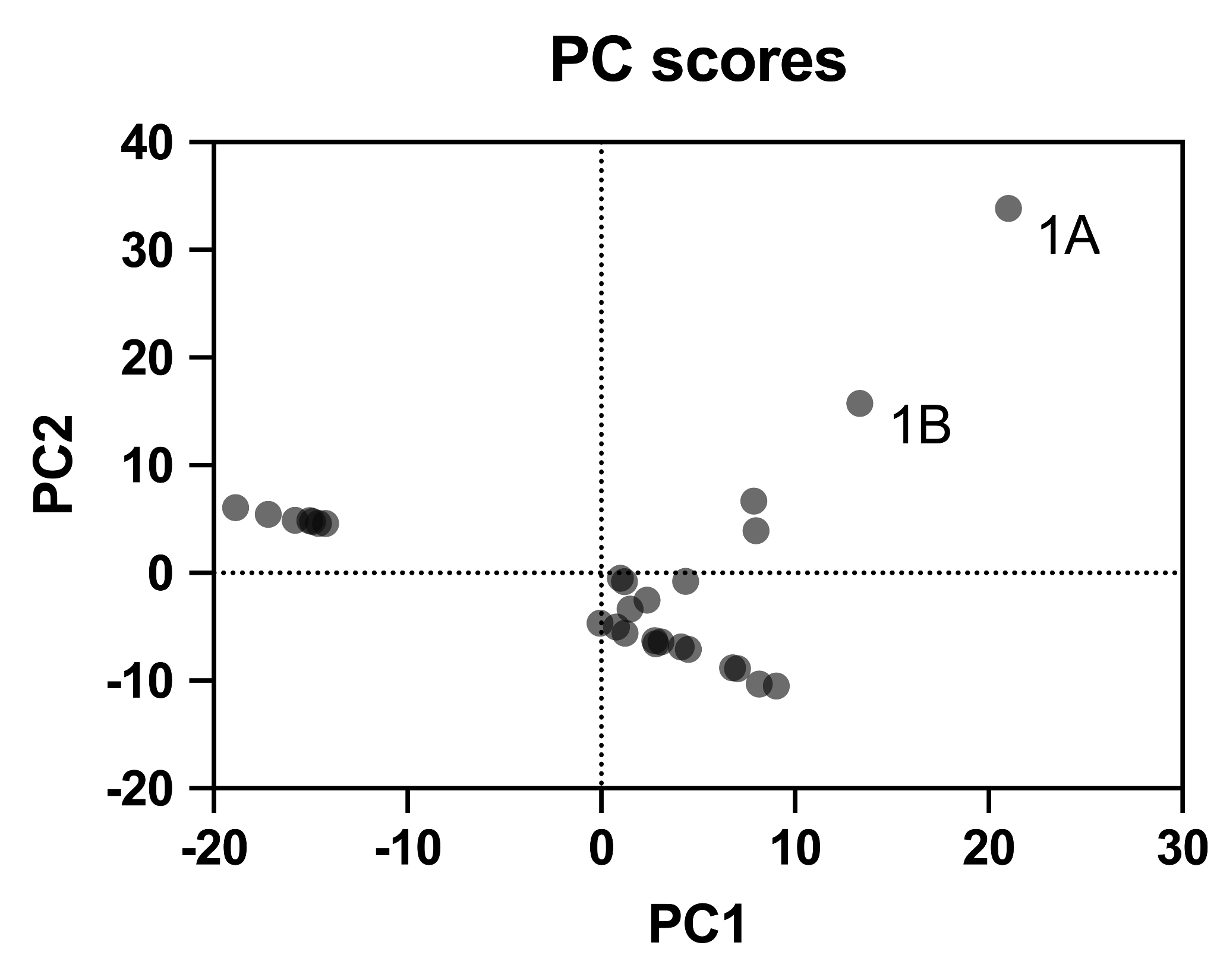


## **Figure S9** Principal Component Analysis (PCA) of crude extracts from *Marinobacterium* sp. YM272 under different culture conditions.


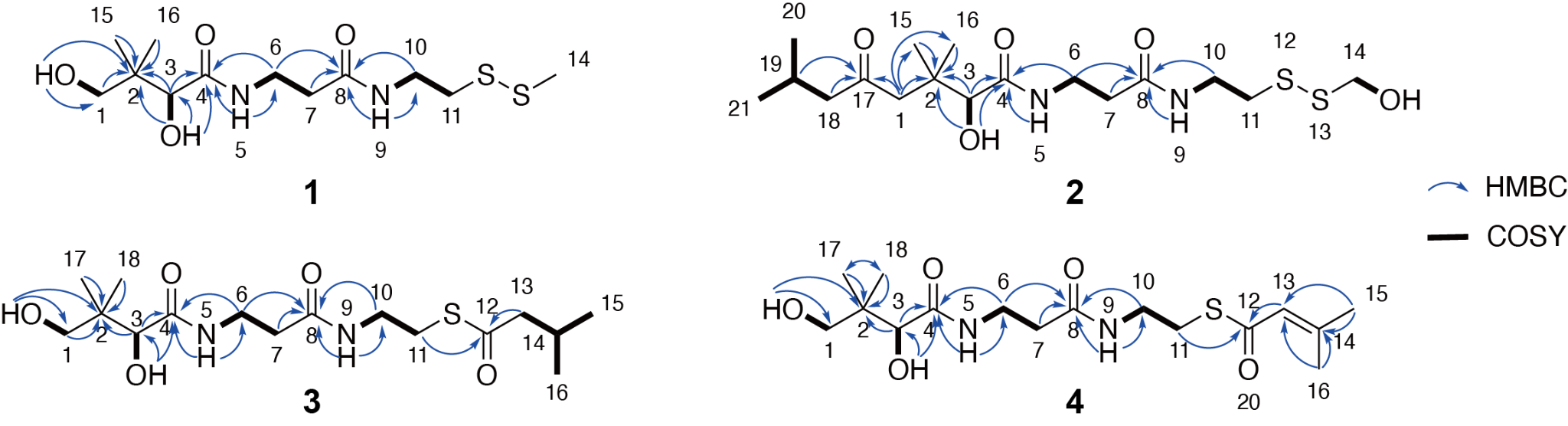


## **Figure S10** COSY and key HMBC correlations for **1**−**4**.


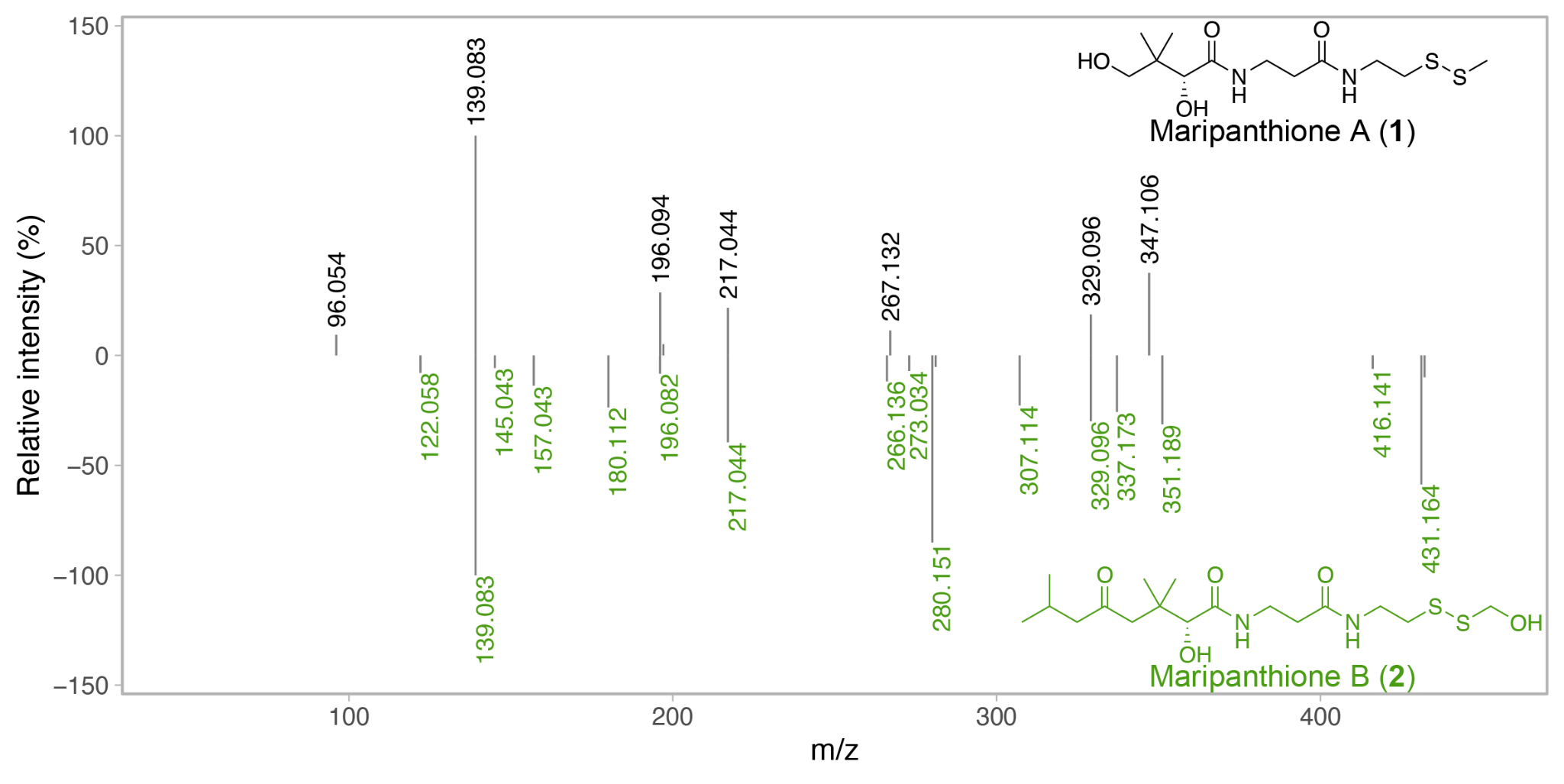


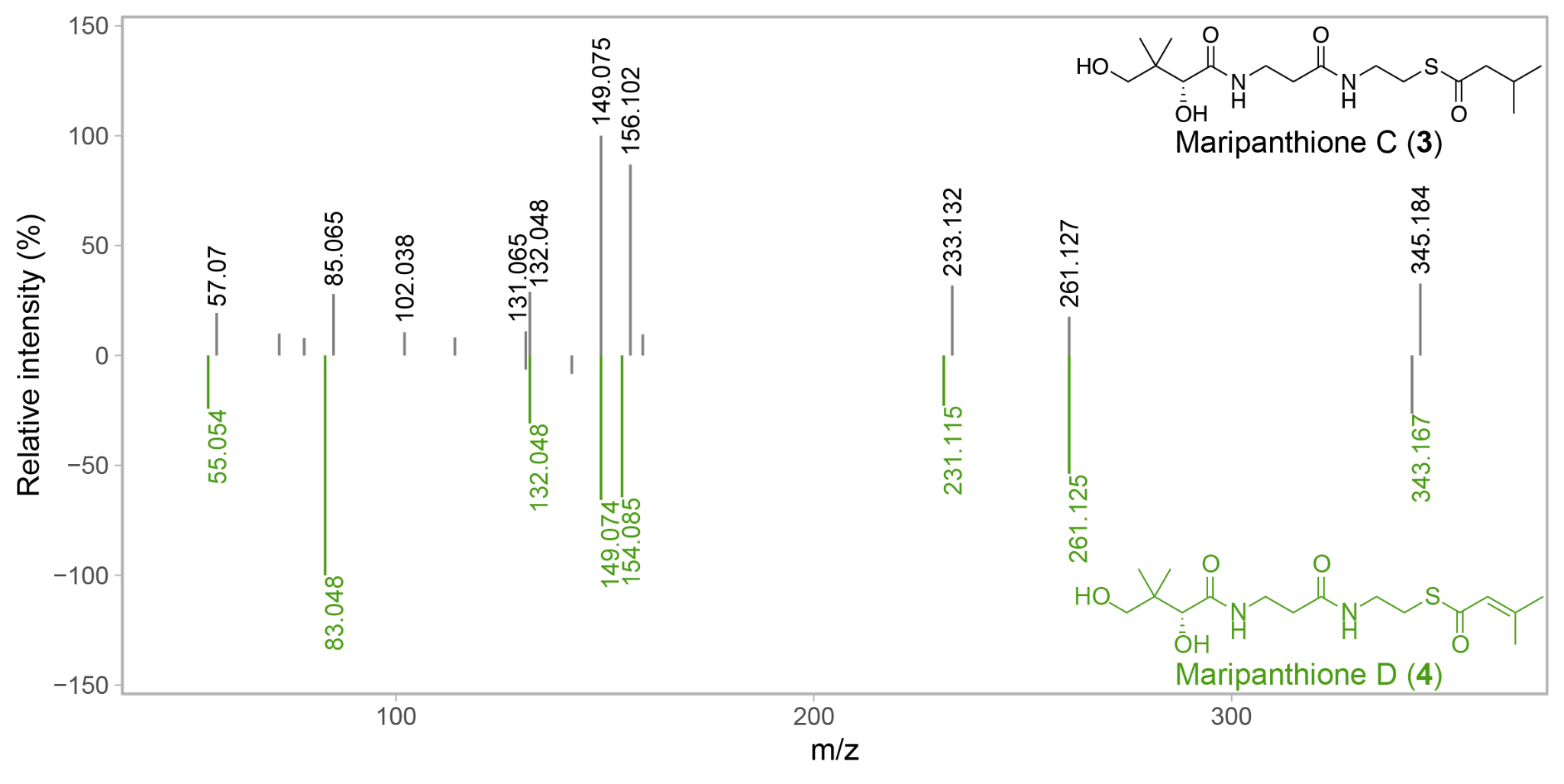


## **Figure S11** MS/MS mirror plot of maripanthione A (**1**) *vs.* B (**2**) and C (**3**) *vs.* D (**4**)


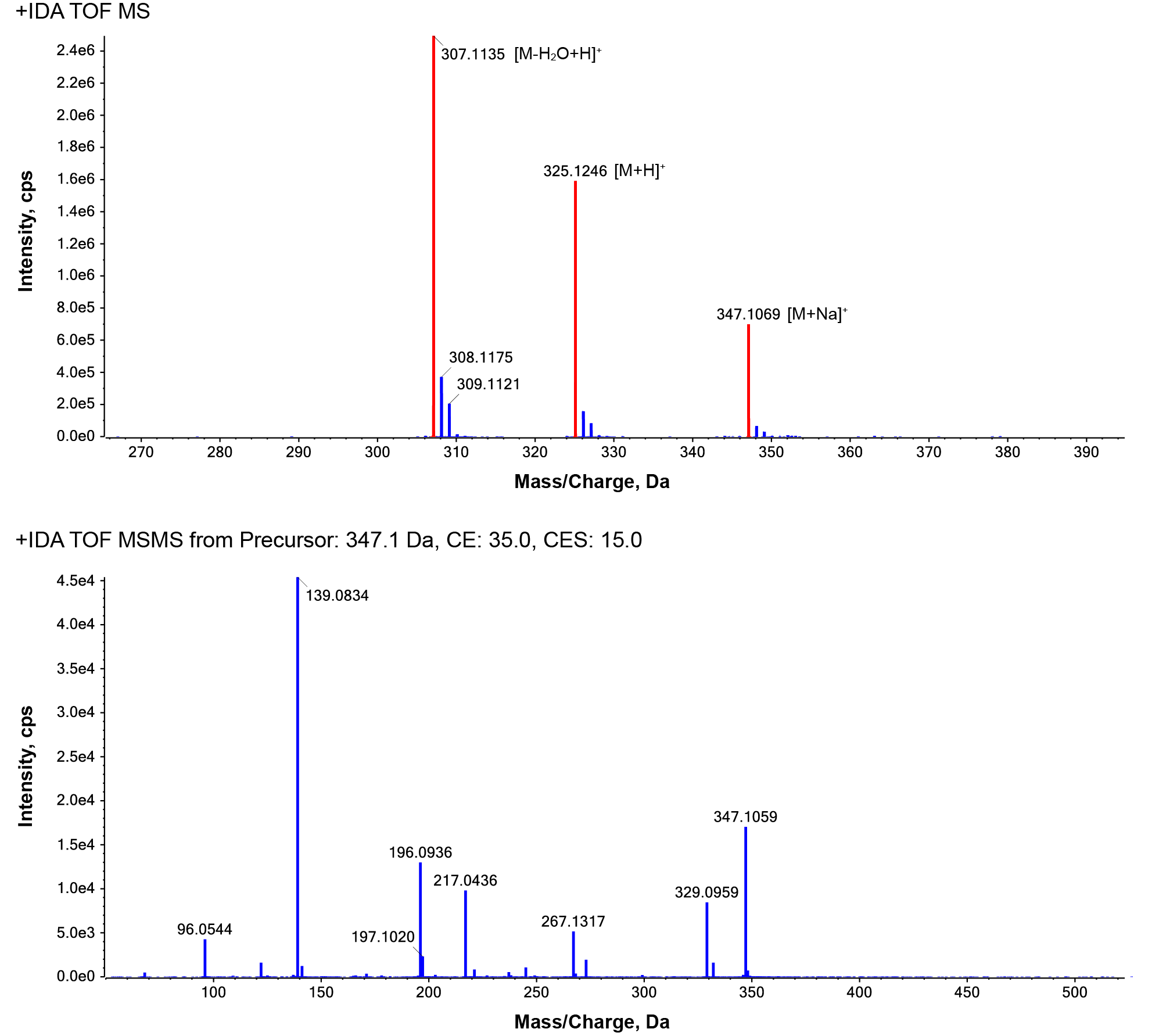


## **Figure S12.1** HR-ESI-MS of maripanthione A (**1**)


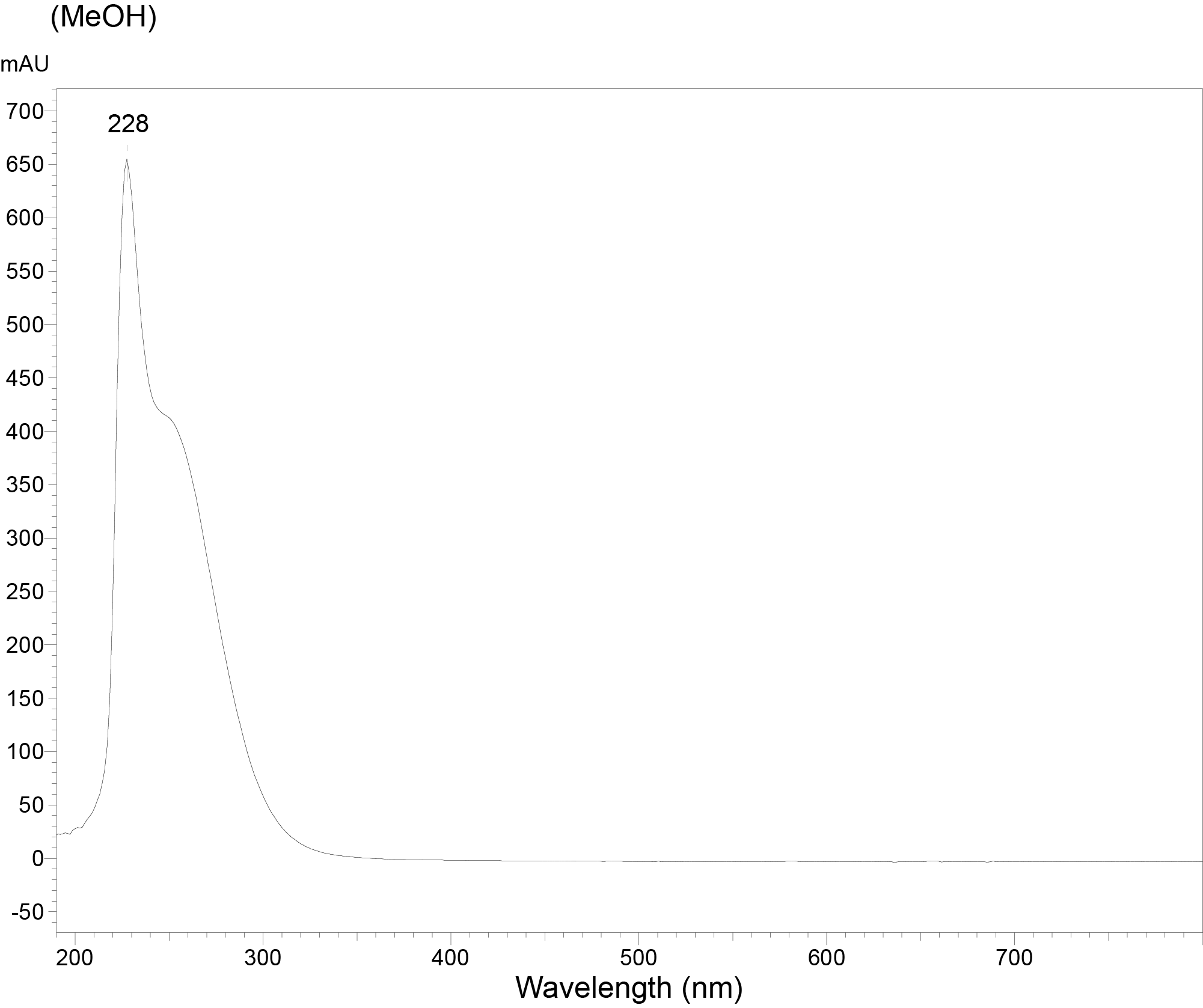


## **Figure S12.2** UV spectrum of **1**


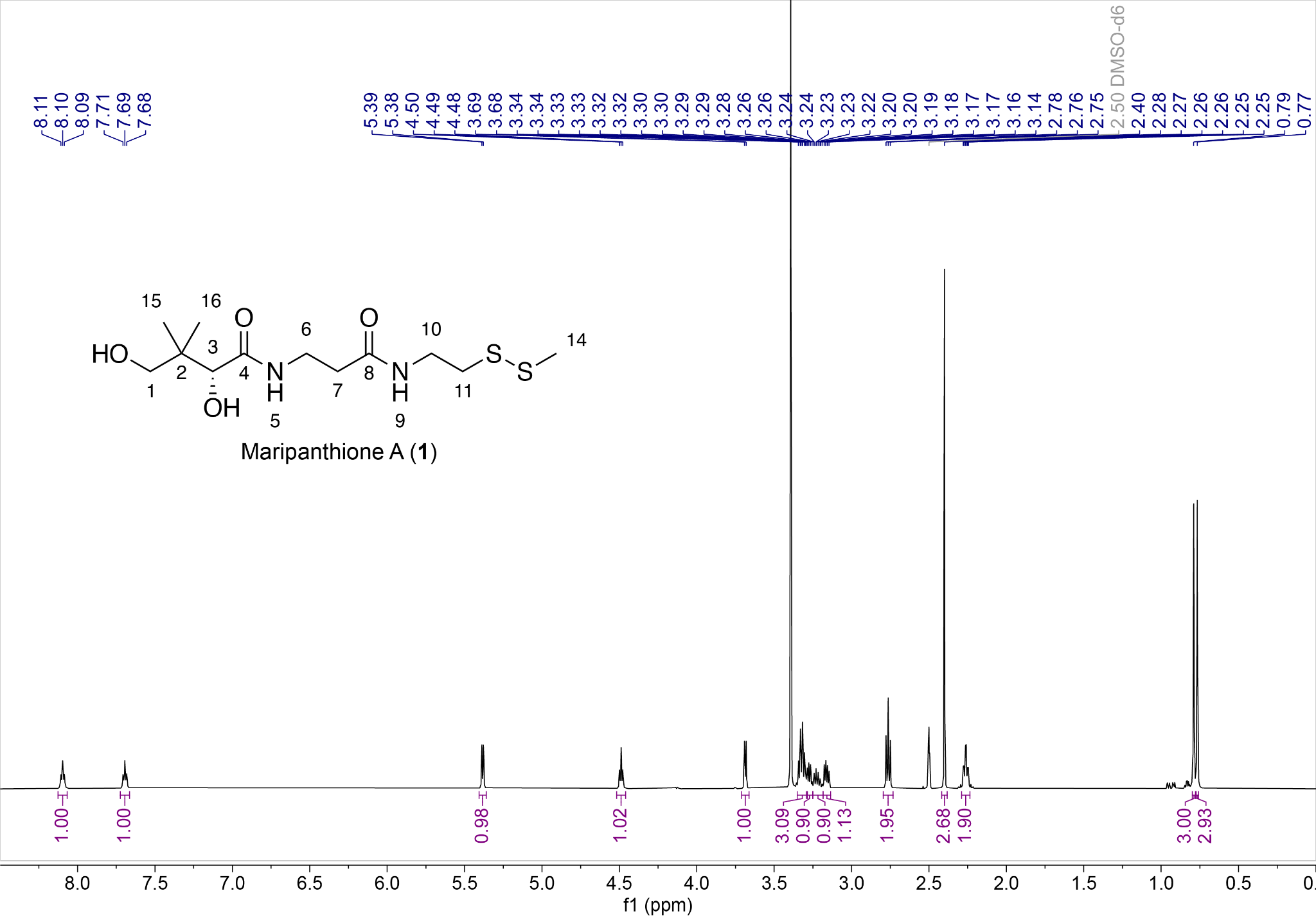


## **Figure S12.3** ^1^H NMR spectrum of **1** (500 MHz, DMSO-*d6*)


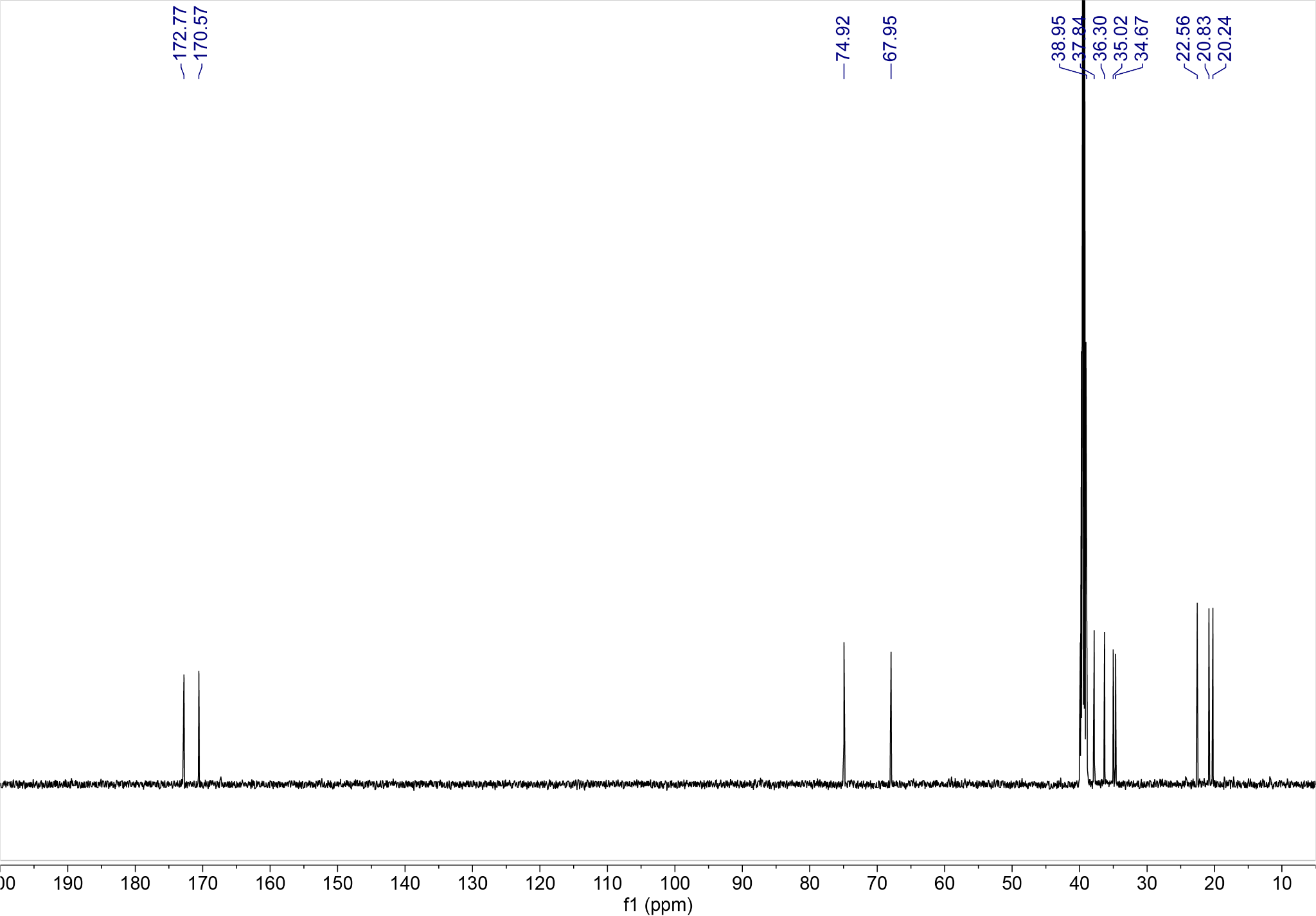


## **Figure S12.4** ^13^C NMR spectrum of **1** (125 MHz, DMSO-*d6*)


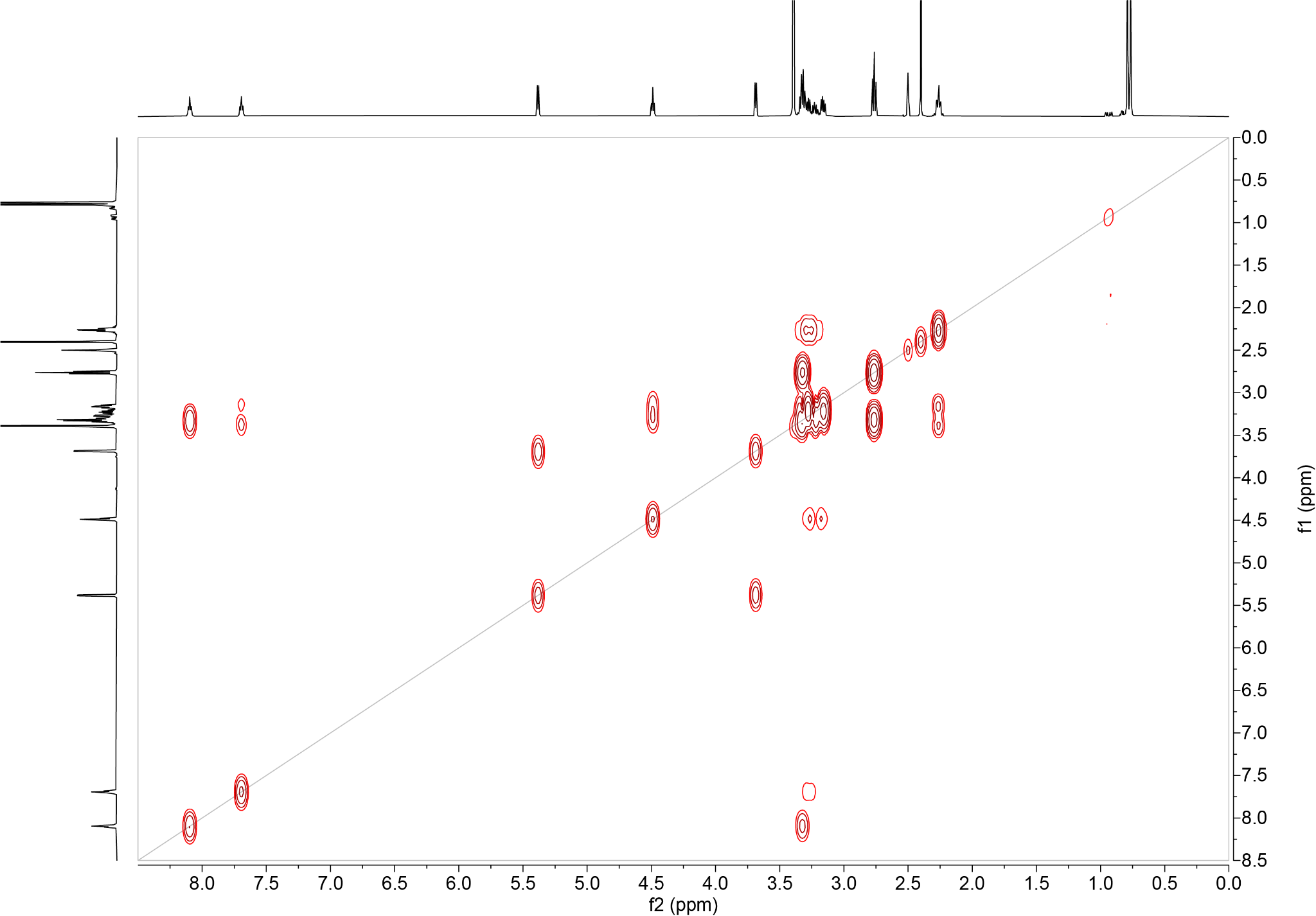


## **Figure S12.5** COSY spectrum of **1** (500 MHz, DMSO-*d6*)


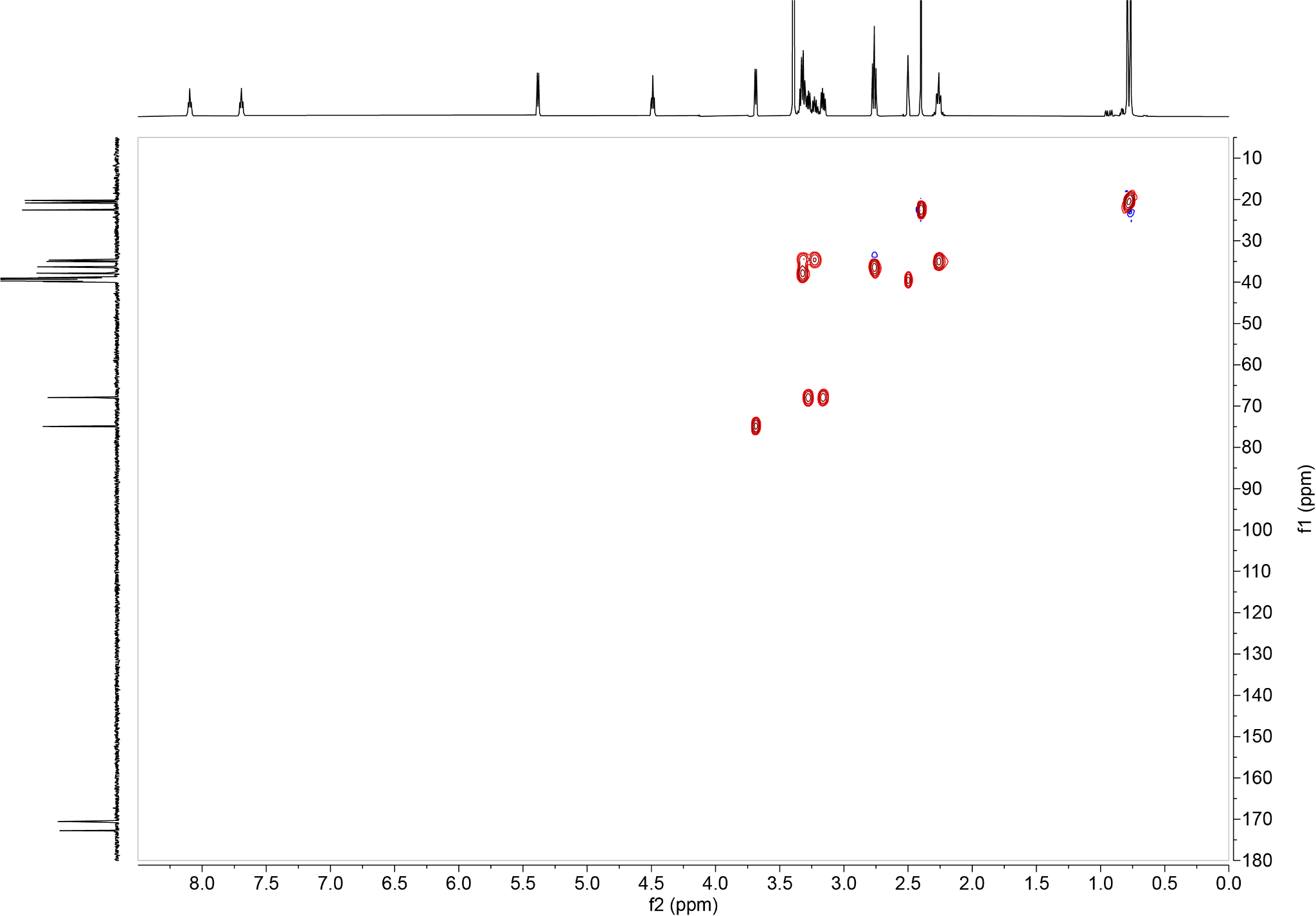


## **Figure S12.6** HSQC spectrum of **1** (500 MHz, DMSO-*d6*)


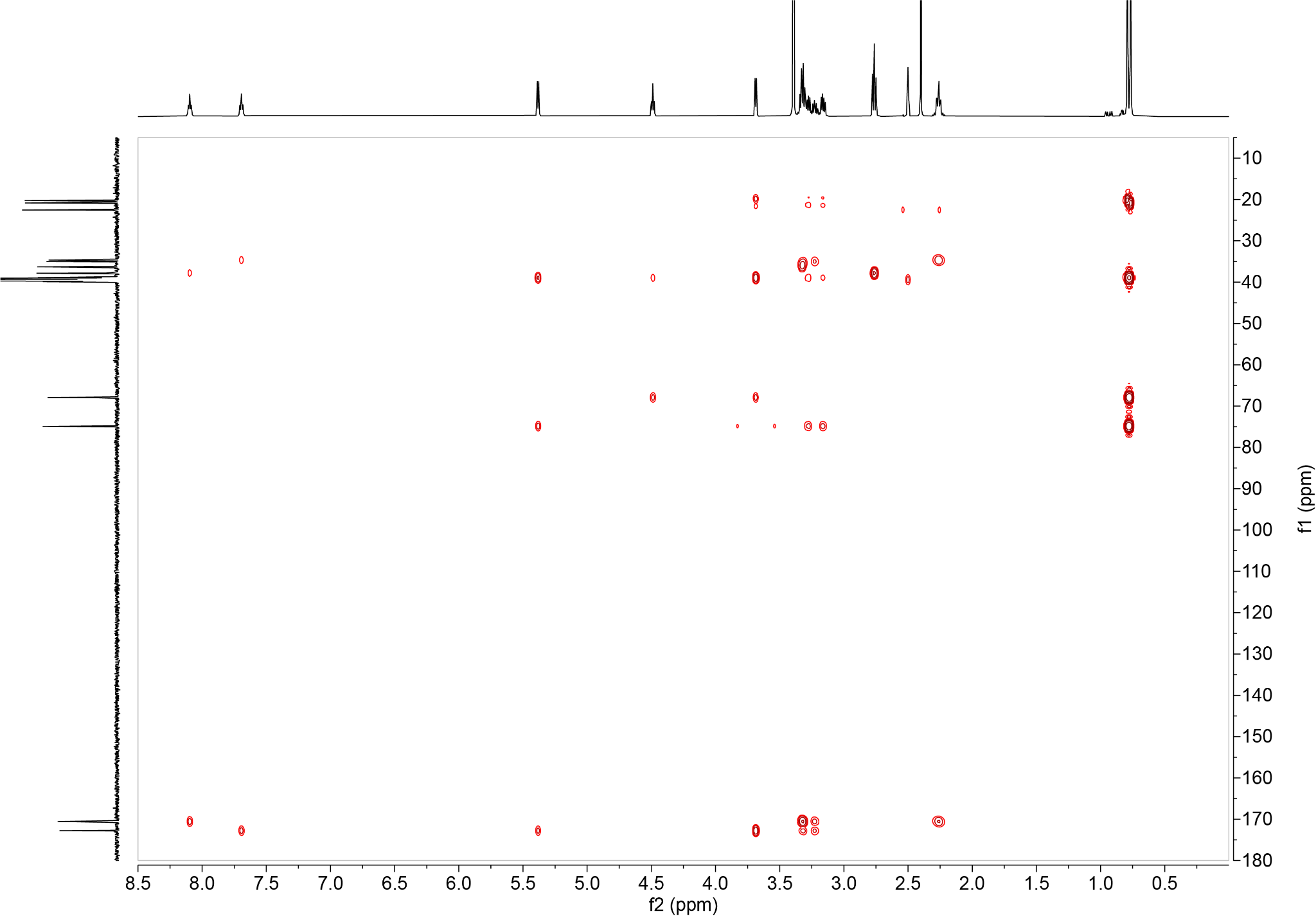


## **Figure S12.7** HMBC spectrum of **1** (500 MHz, DMSO-*d6*)

## **Figure S12.8** ATR-FTIR spectrum of **1**


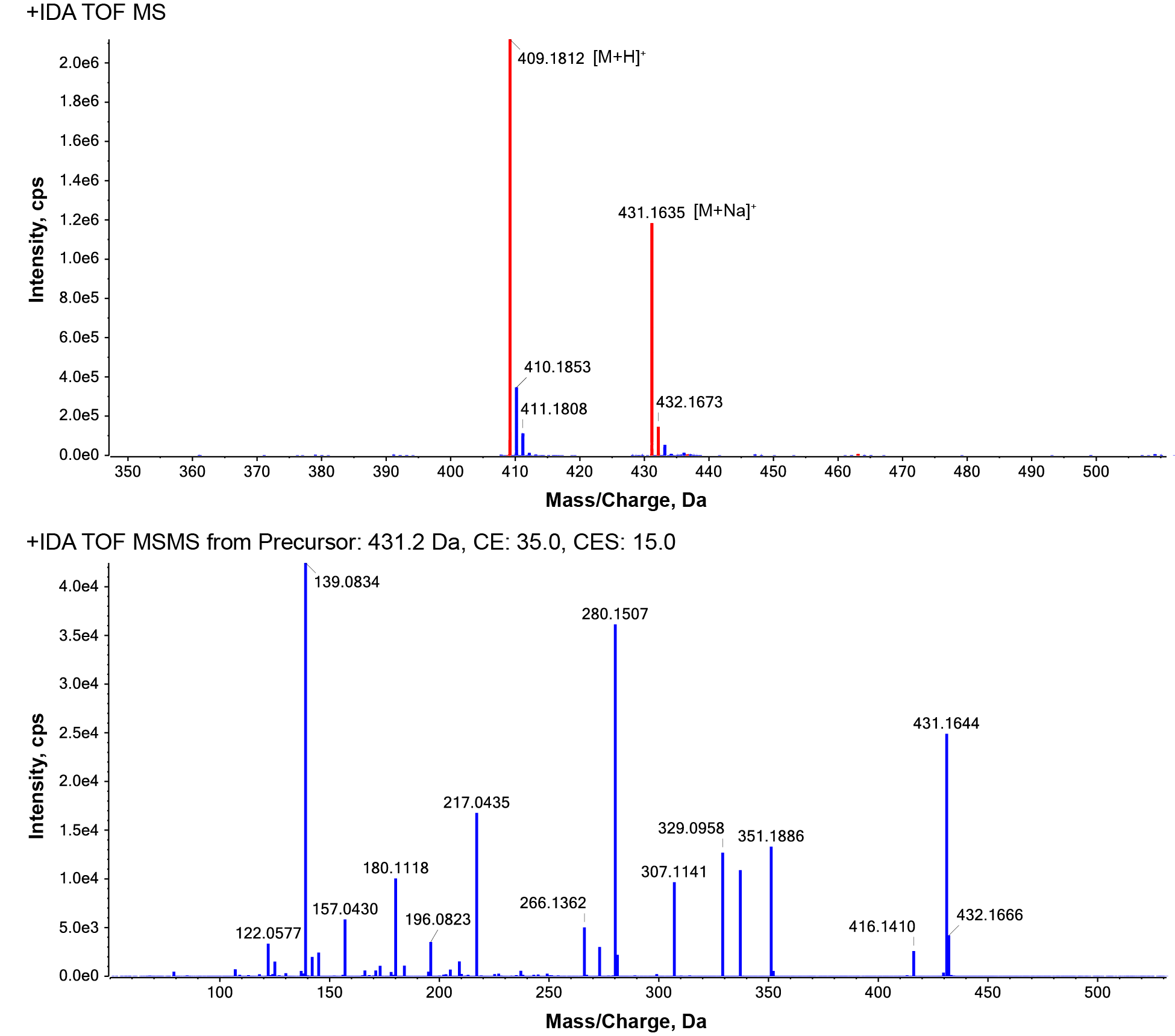


## **Figure S13.1** HR-ESI-MS of maripanthione B (**2**)


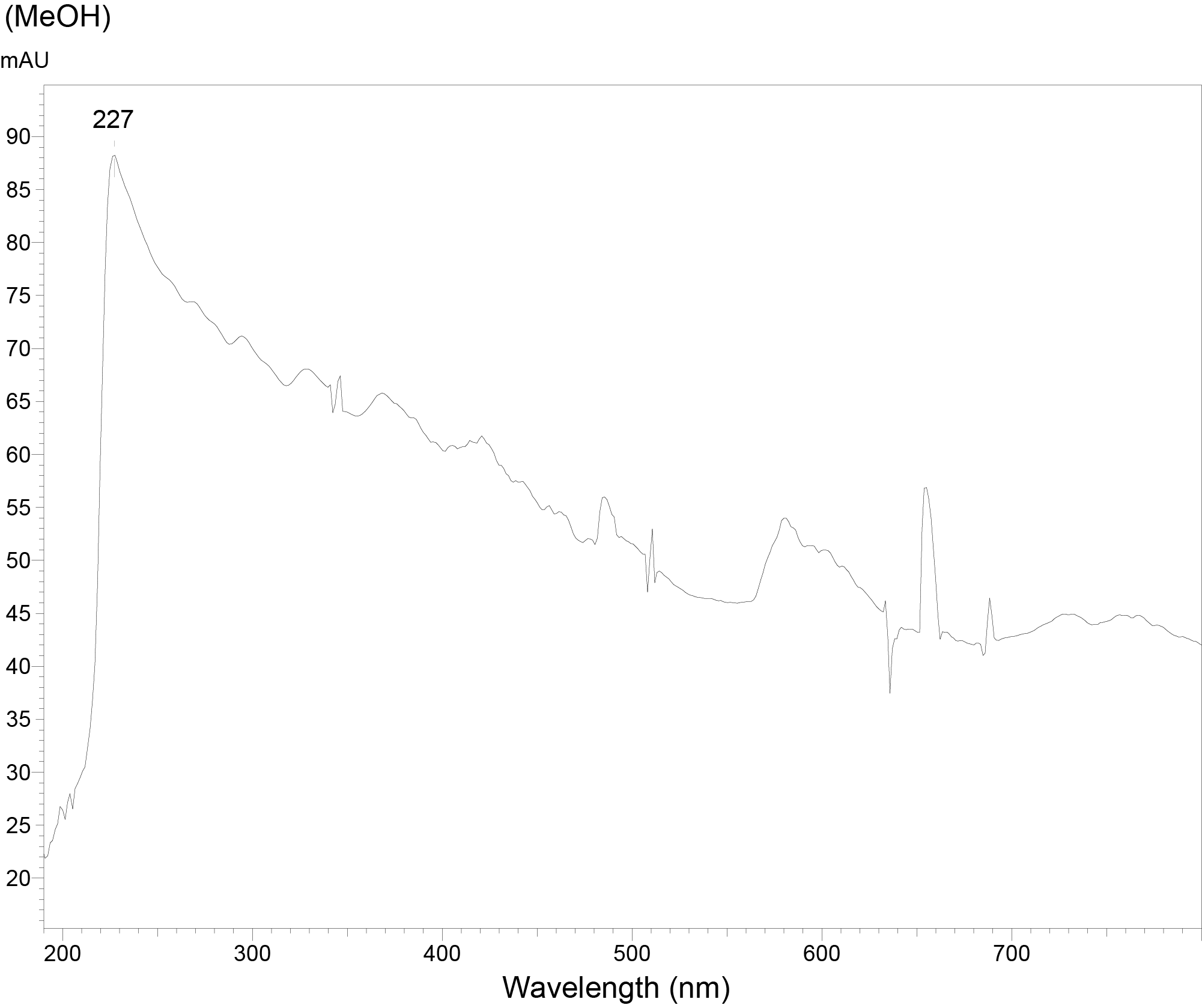


## **Figure S13.2** UV spectrum of **2**


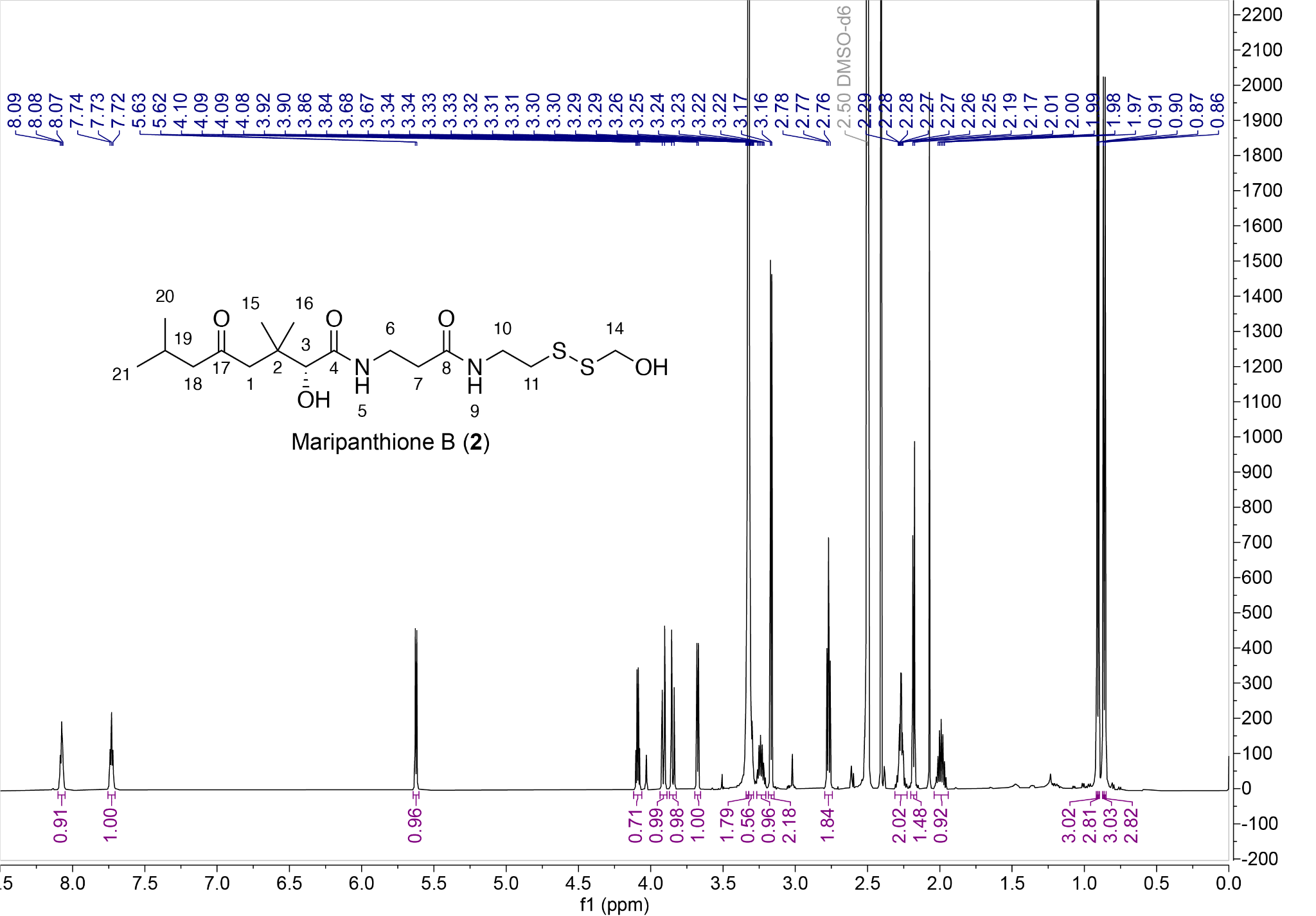


## **Figure S13.3** ^1^H NMR spectrum of **2** (600 MHz, DMSO-*d6*)


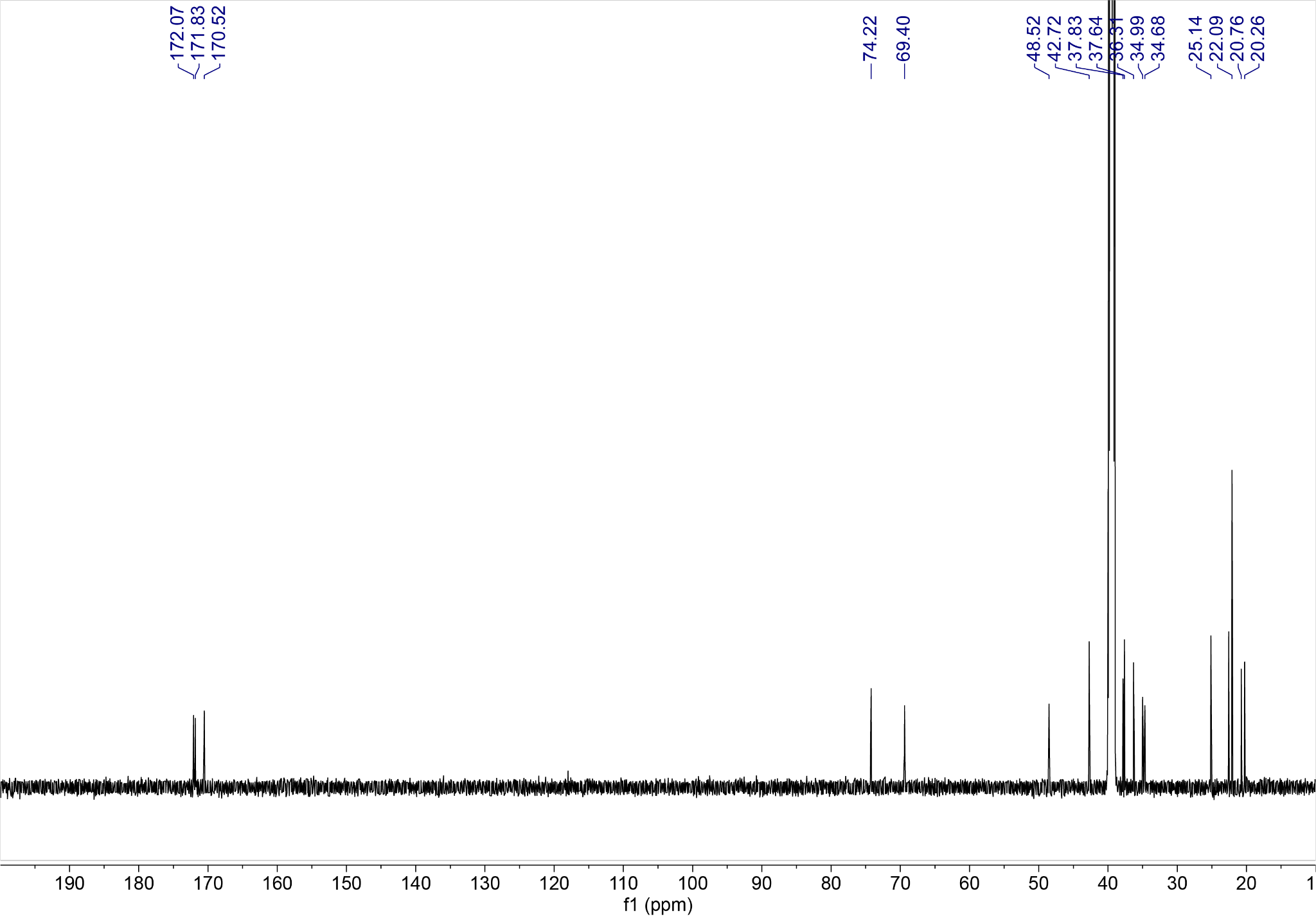


## **Figure S13.4** ^13^C NMR spectrum of **2** (150 MHz, DMSO-*d6*)


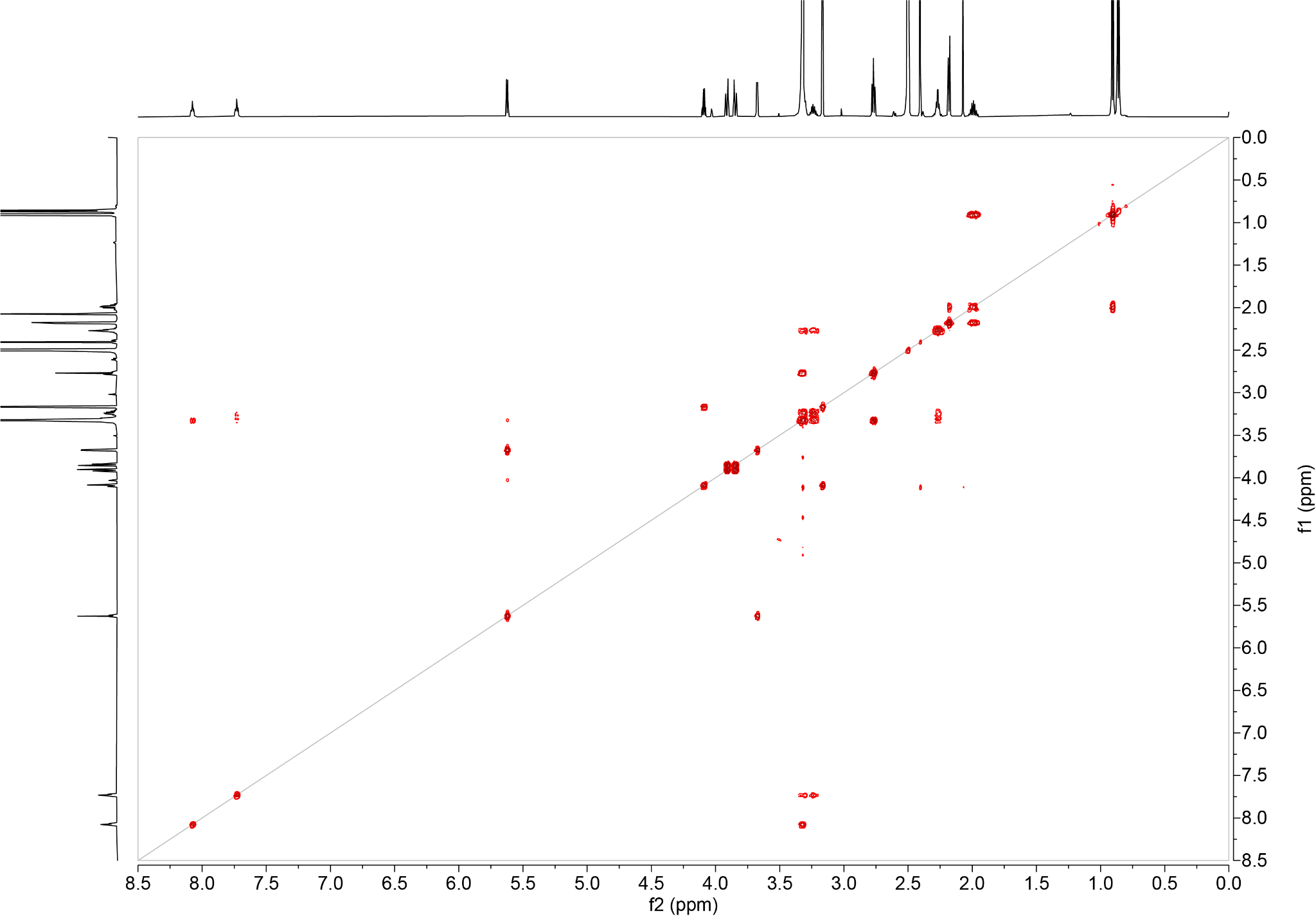


## **Figure S13.5** COSY spectrum of **2** (600 MHz, DMSO-*d6*)


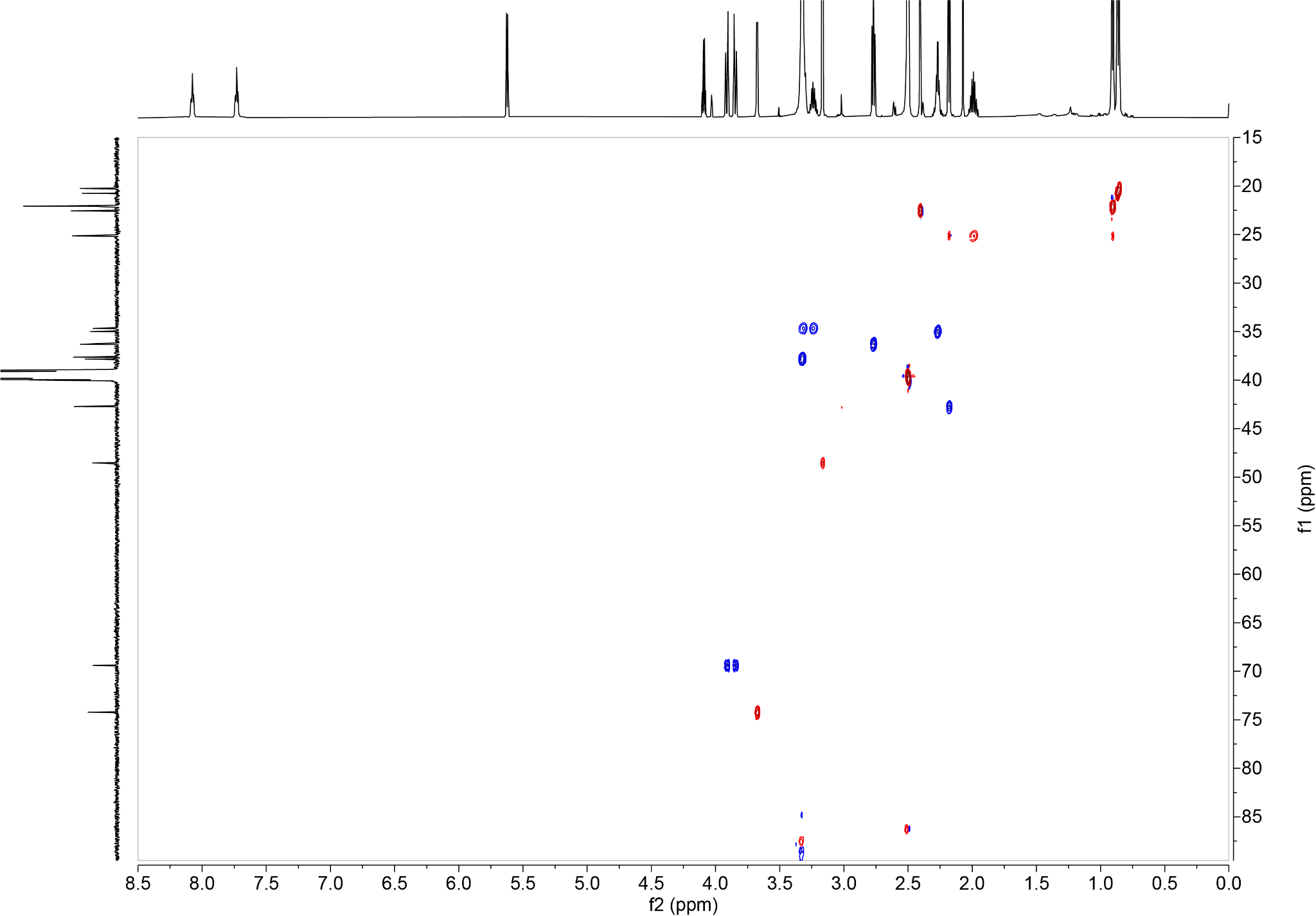


## **Figure S13.6** HSQC-EDITED spectrum of **2** (600 MHz, DMSO-*d6*)


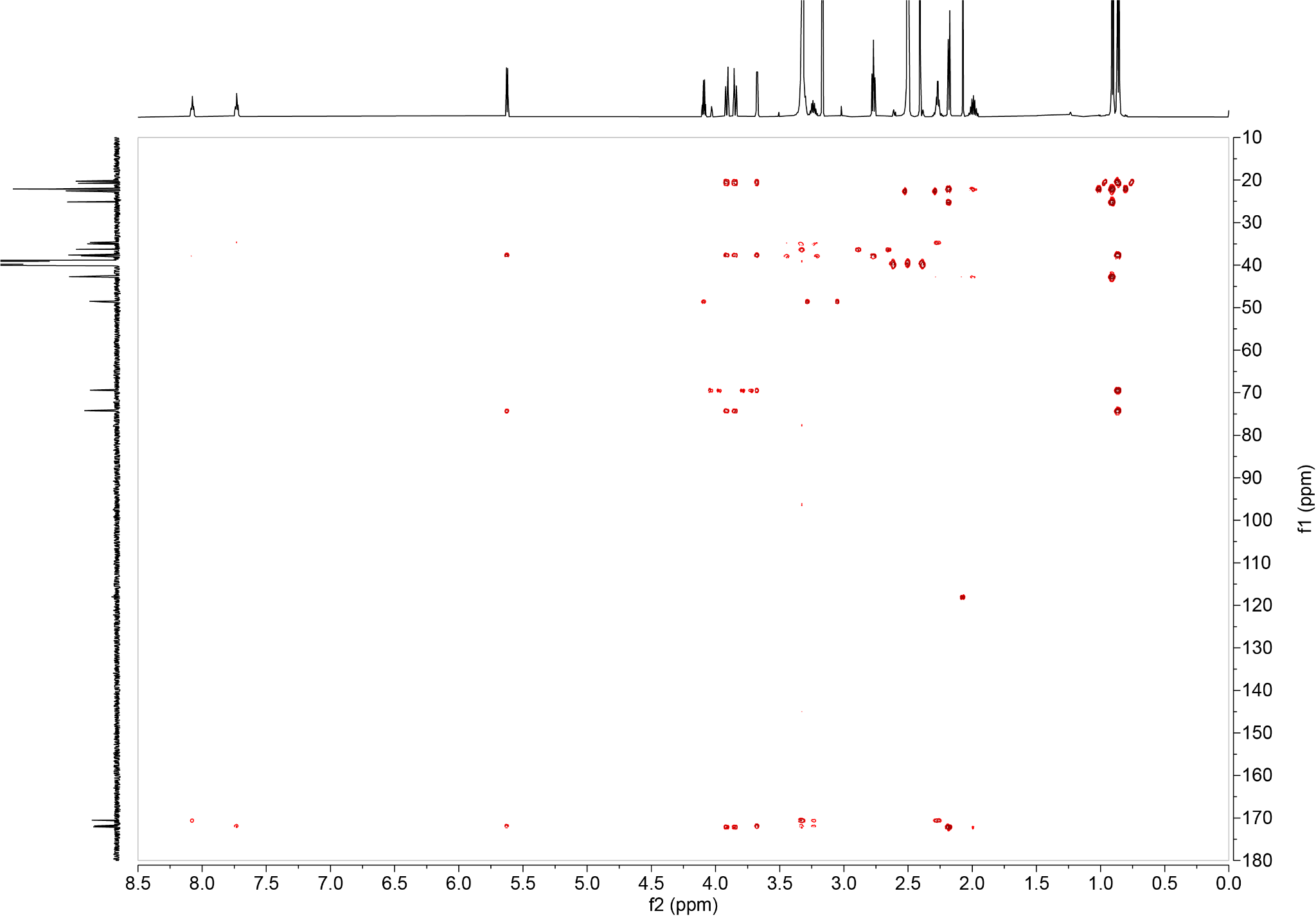


## **Figure S13.7** HMBC spectrum of **2** (600 MHz, DMSO-*d6*)

## **Figure S13.8** ATR-FTIR spectrum of **2**


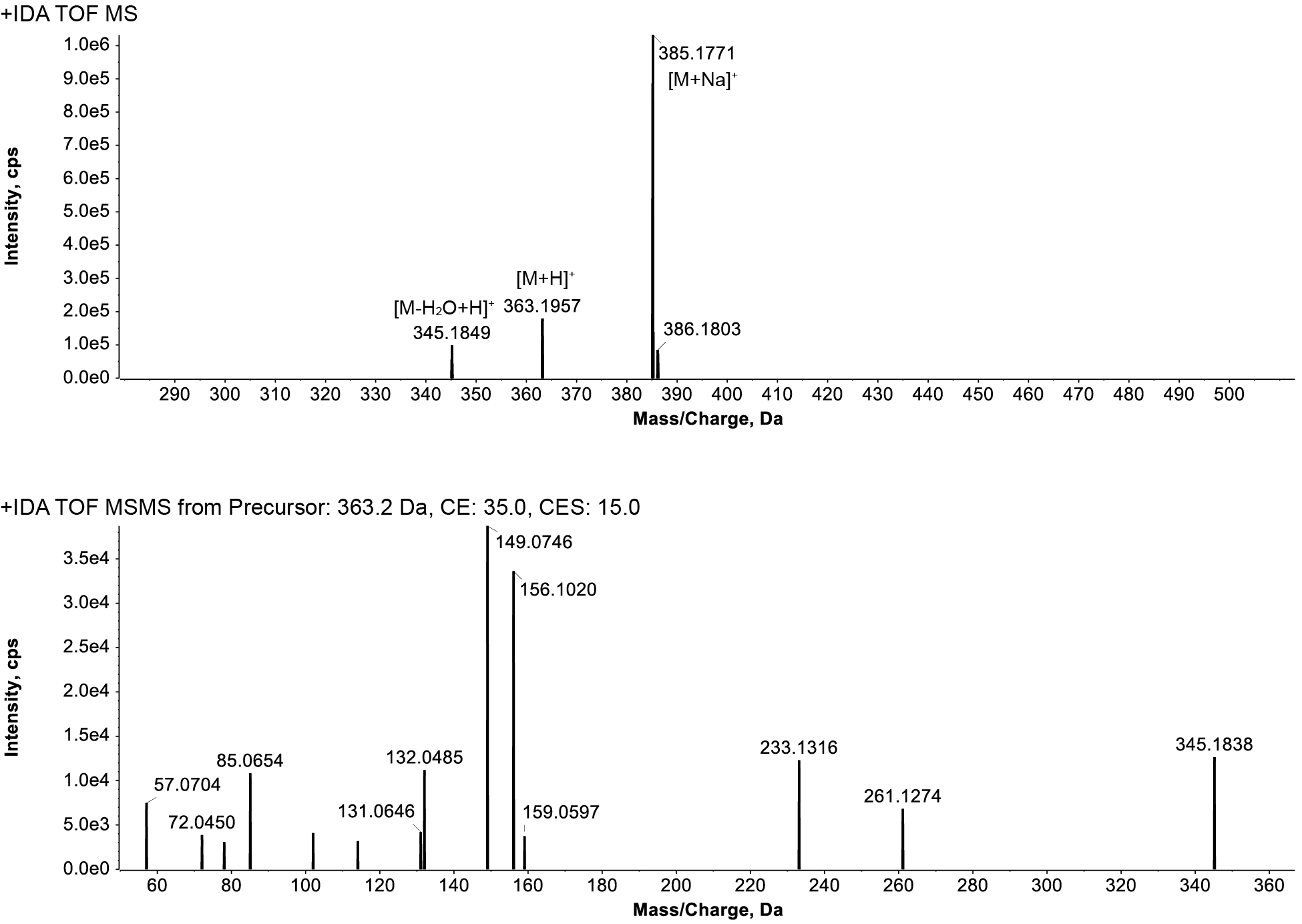


## **Figure S14.1** HR-ESI-MS of maripanthione C (**3**)


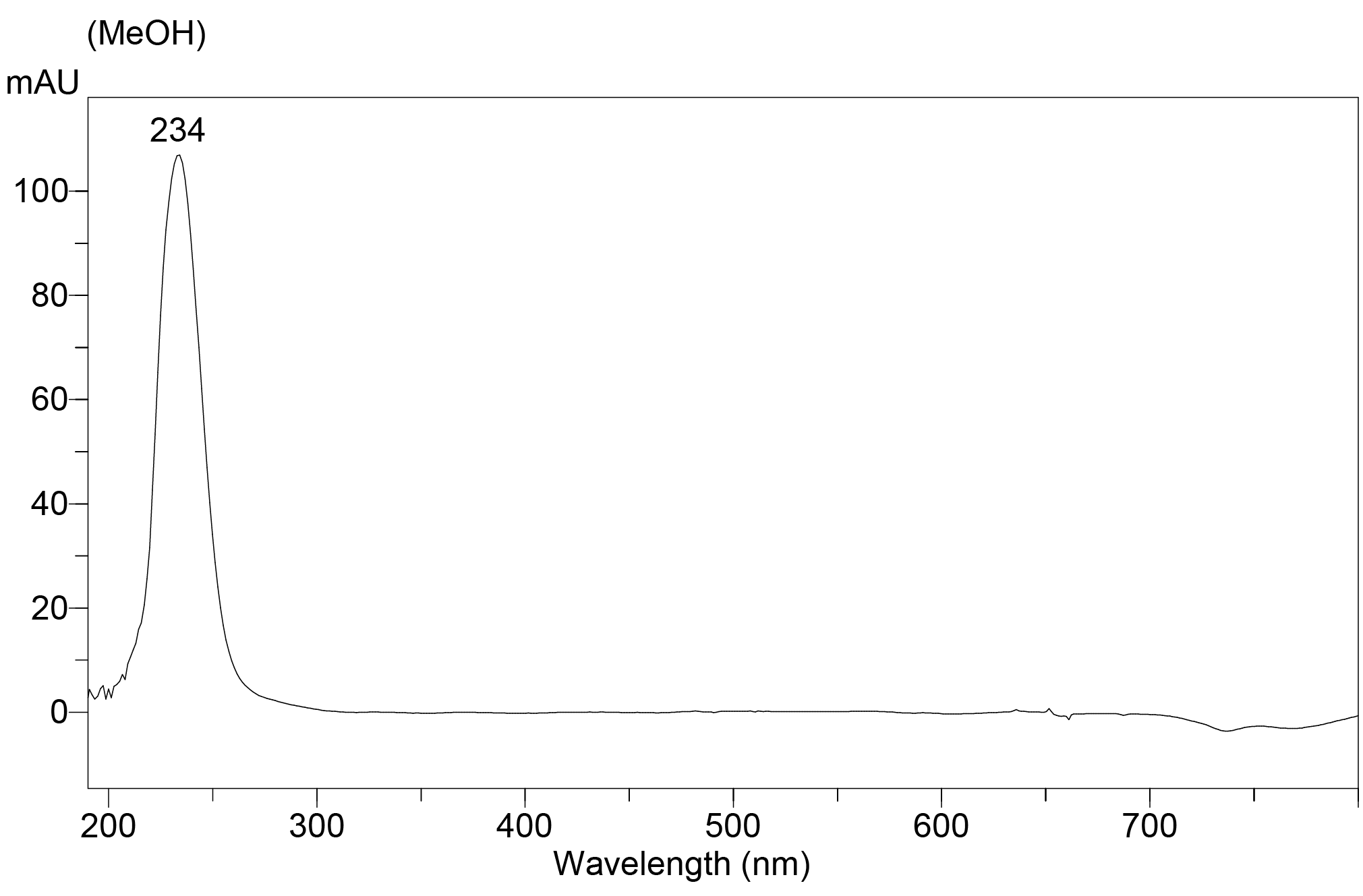


## **Figure S14.2** UV spectrum of **3**


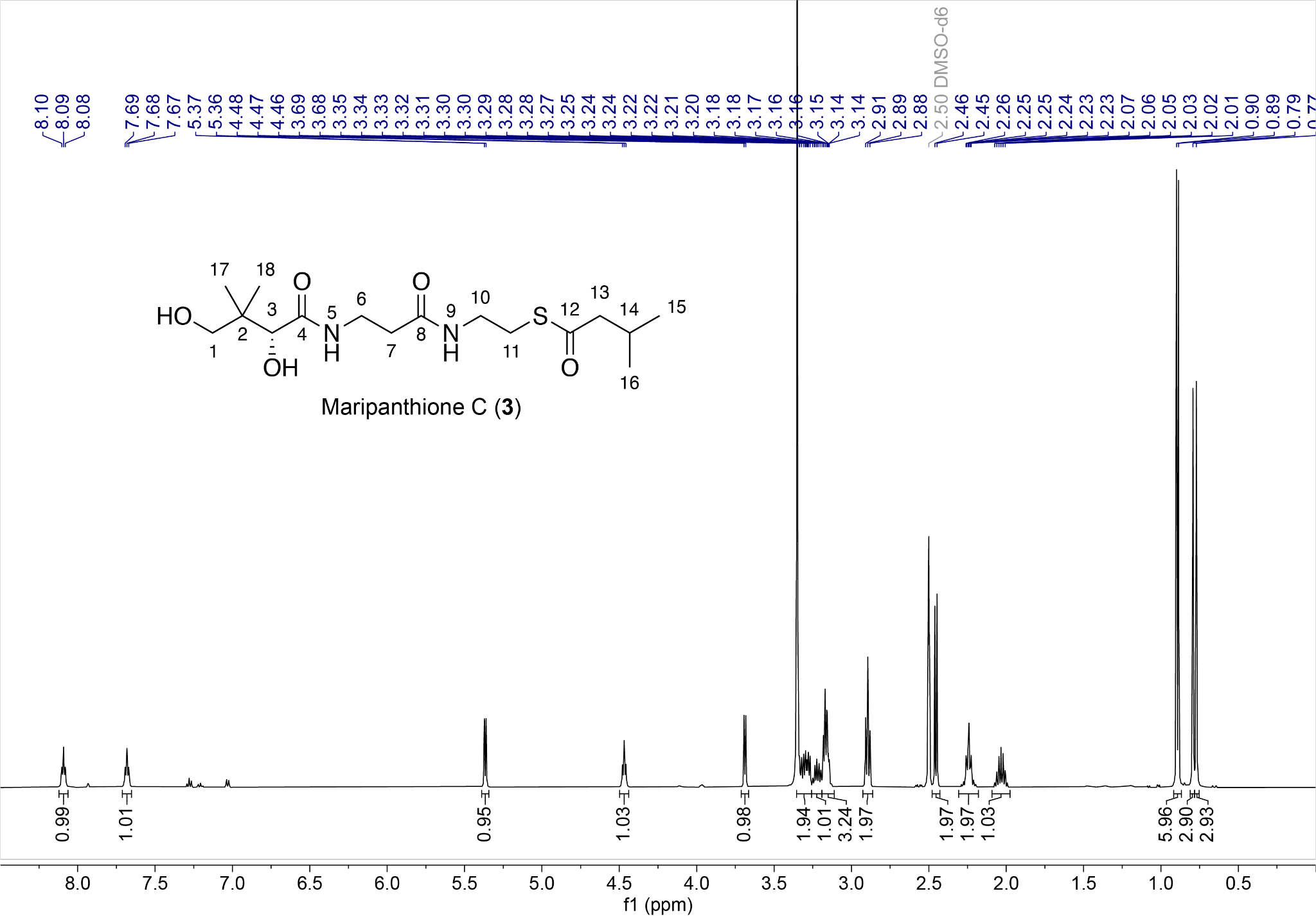


## **Figure S14.3** ^1^H NMR spectrum of **3** (500 MHz, DMSO-*d6*)


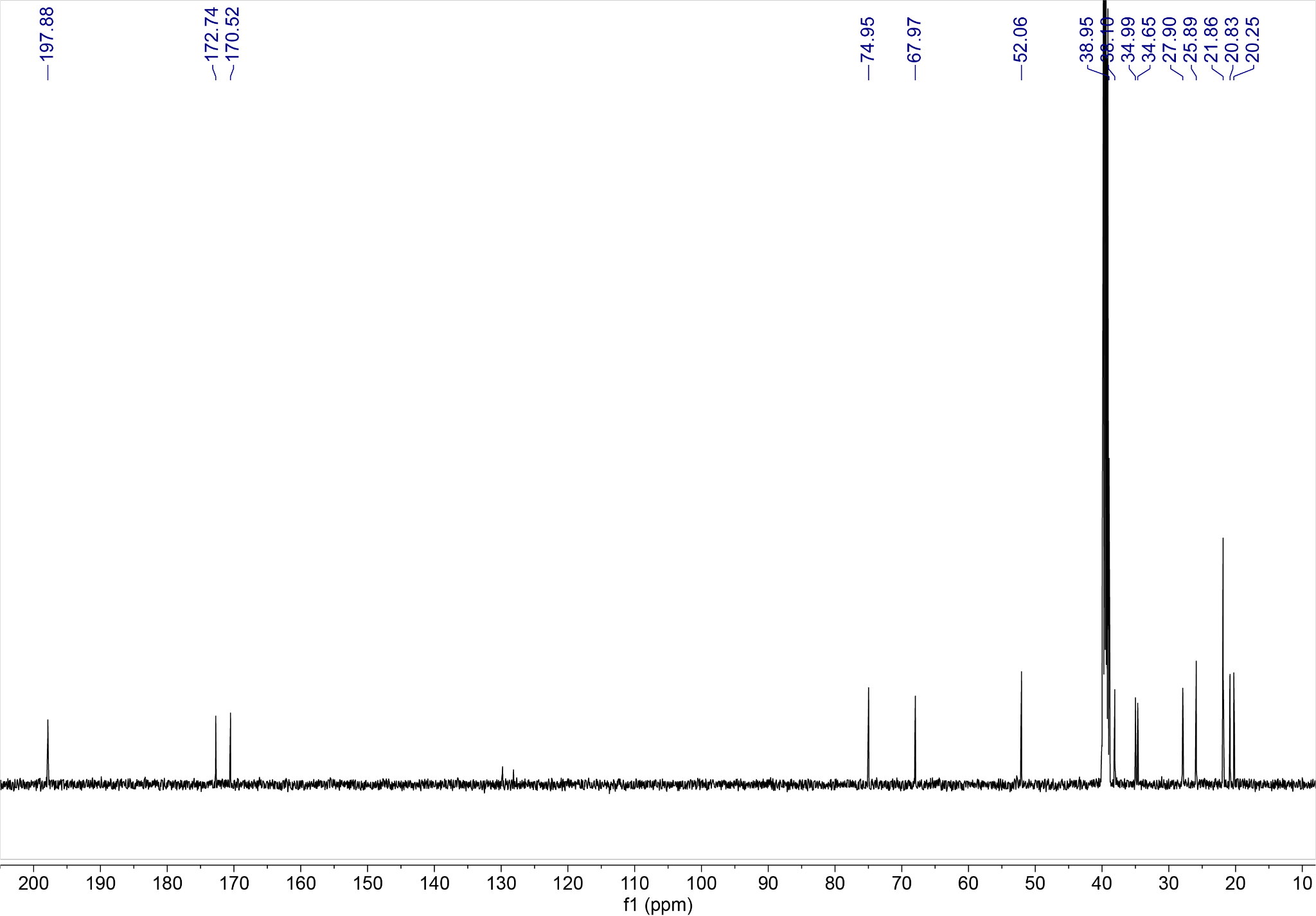


## **Figure S14.4** ^13^C NMR spectrum of **3** (125 MHz, DMSO-*d6*)


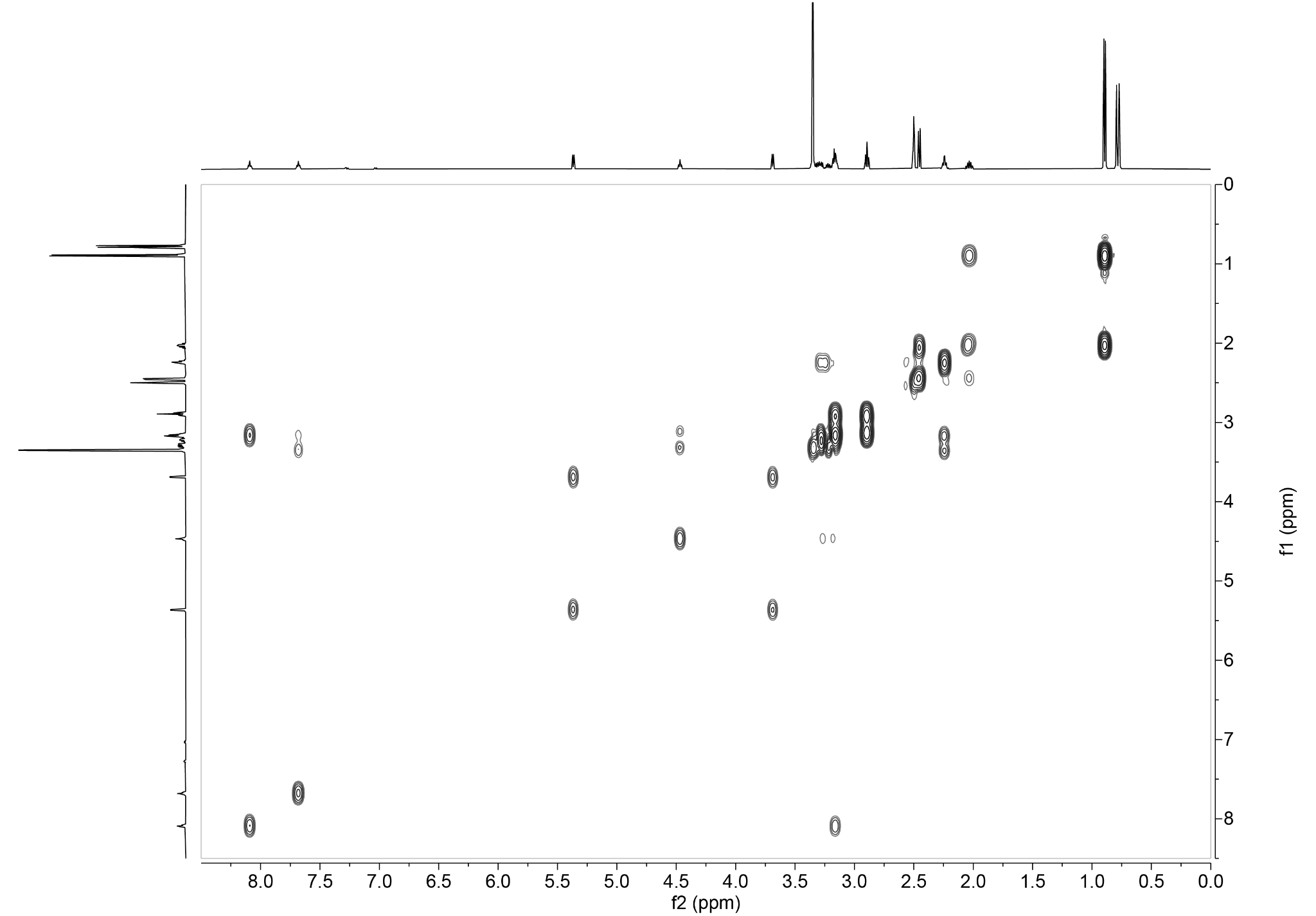


## **Figure S14.5** COSY spectrum of **3** (500 MHz, DMSO-*d6*)


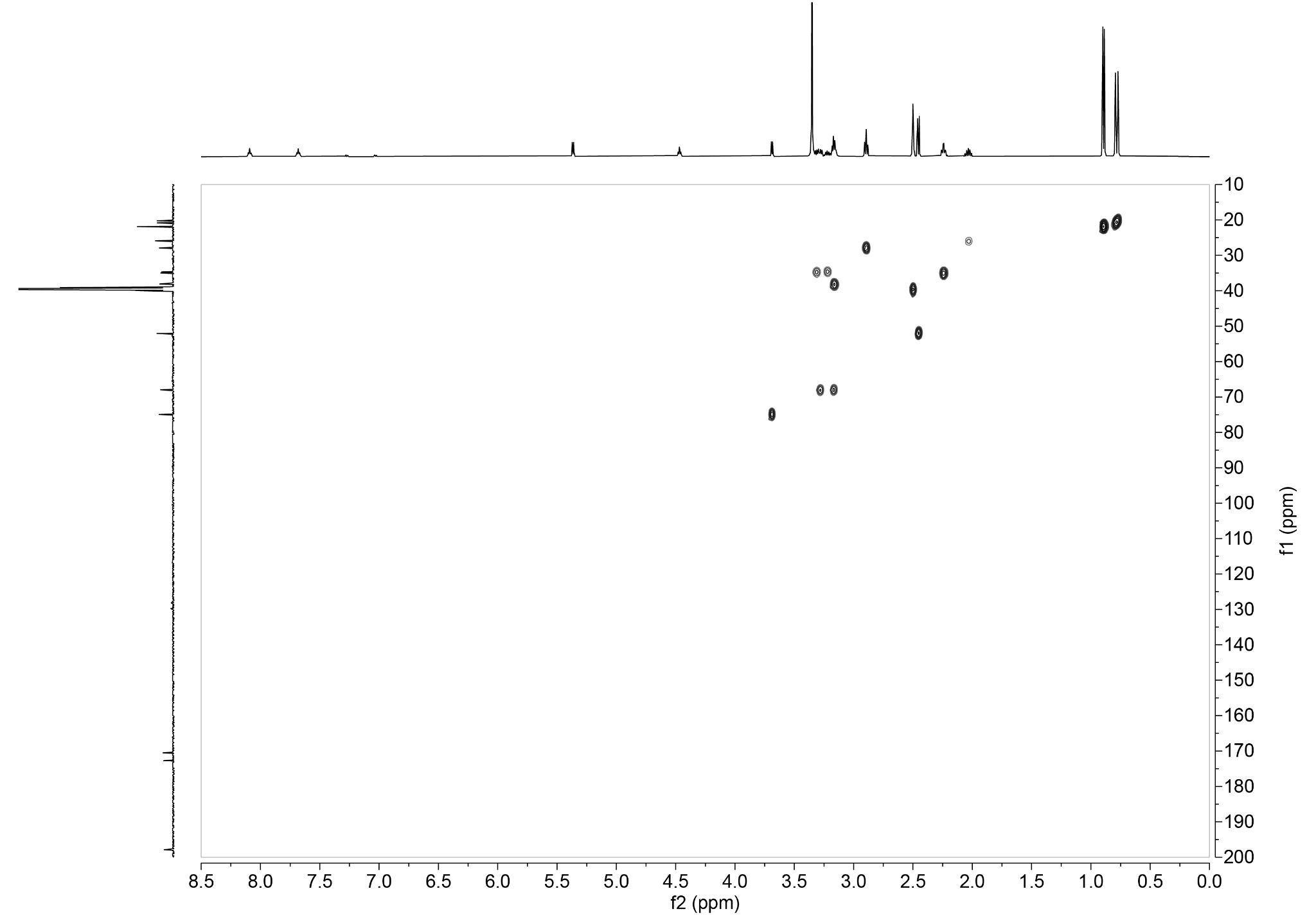


## **Figure S14.6** HSQC spectrum of **3** (500 MHz, DMSO-*d6*)


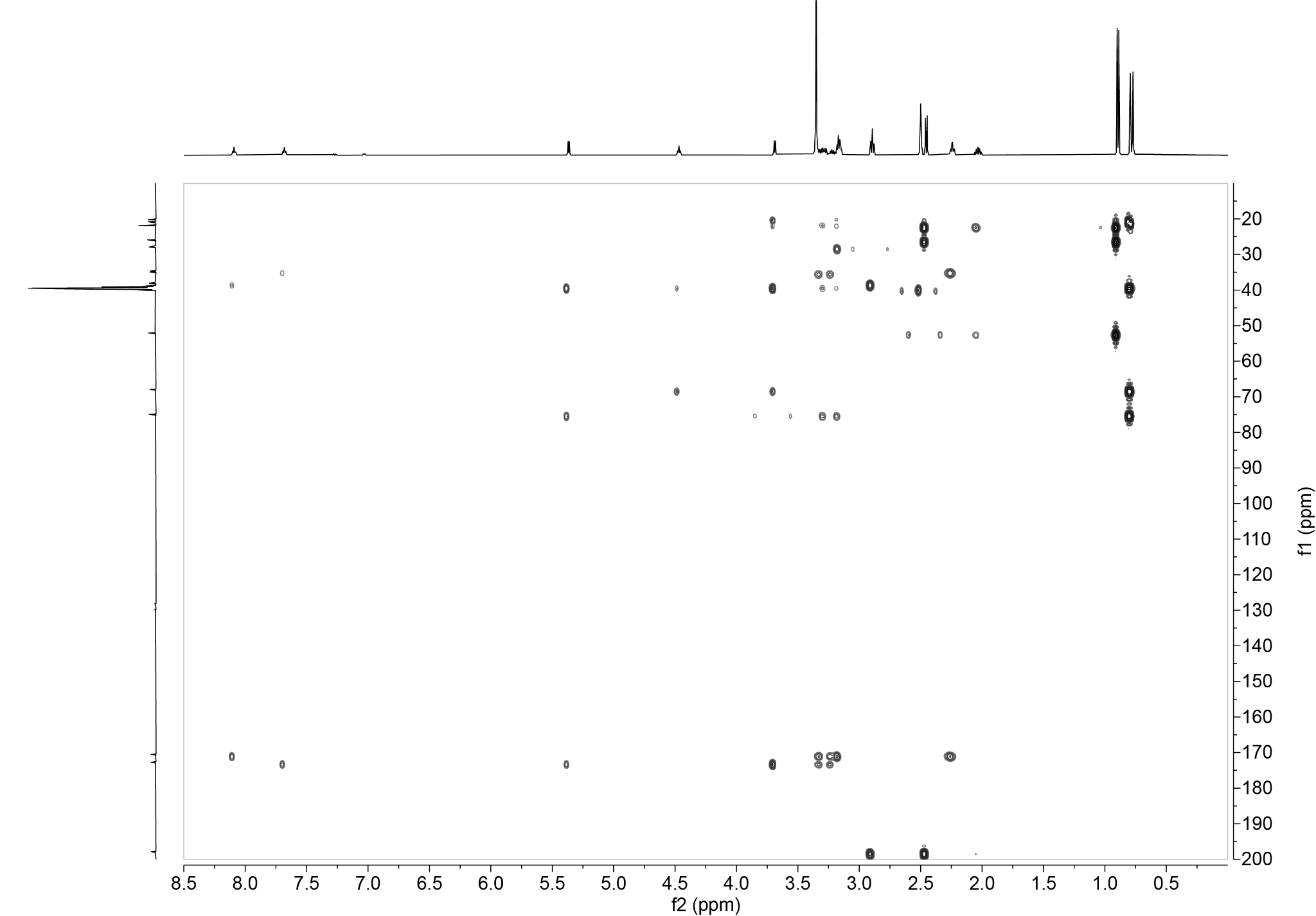


## **Figure S14.7** HMBC spectrum of **3** (500 MHz, DMSO-*d6*)


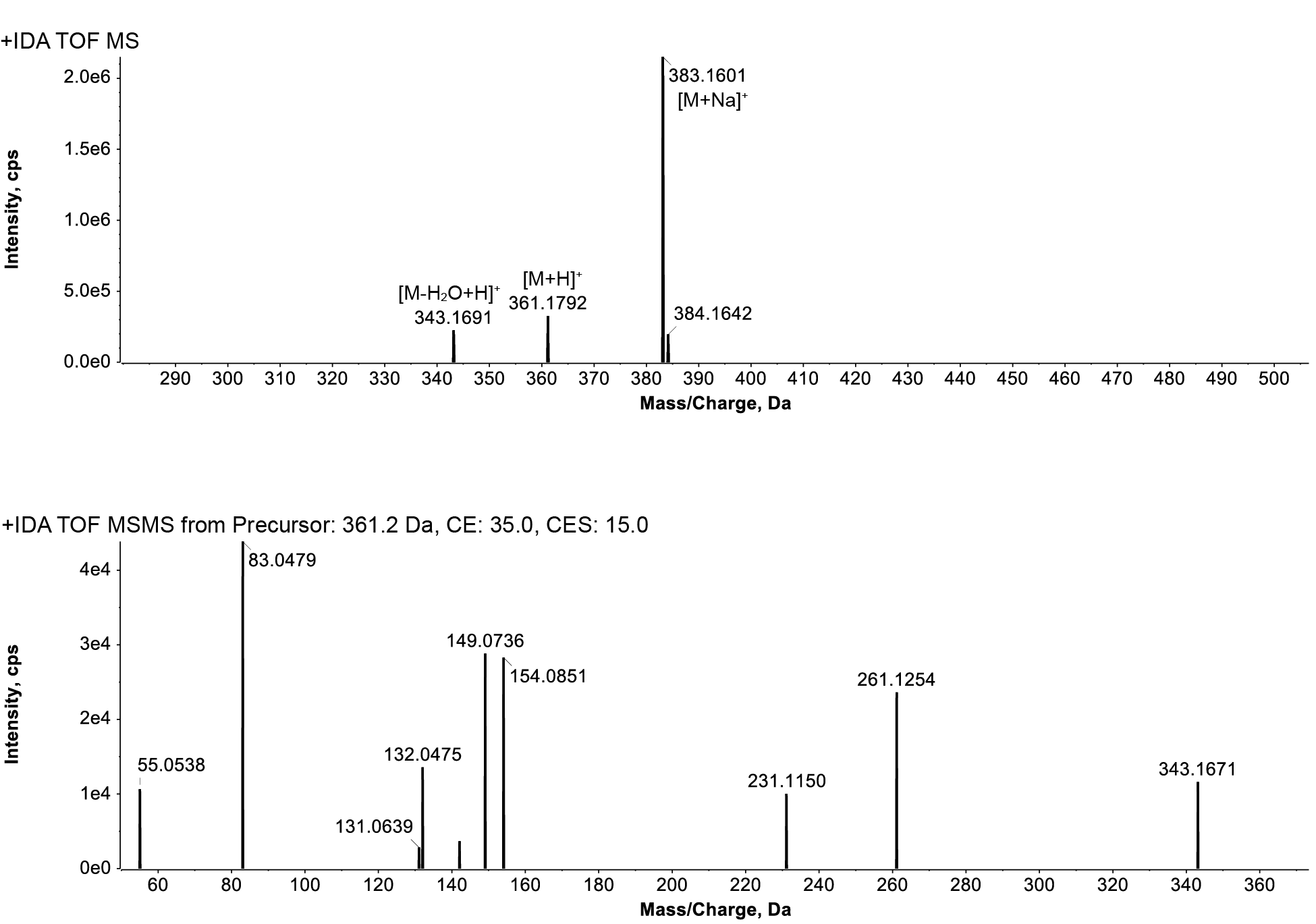


## **Figure S15.1** HR-ESI-MS of maripanthione D (**4**)

**
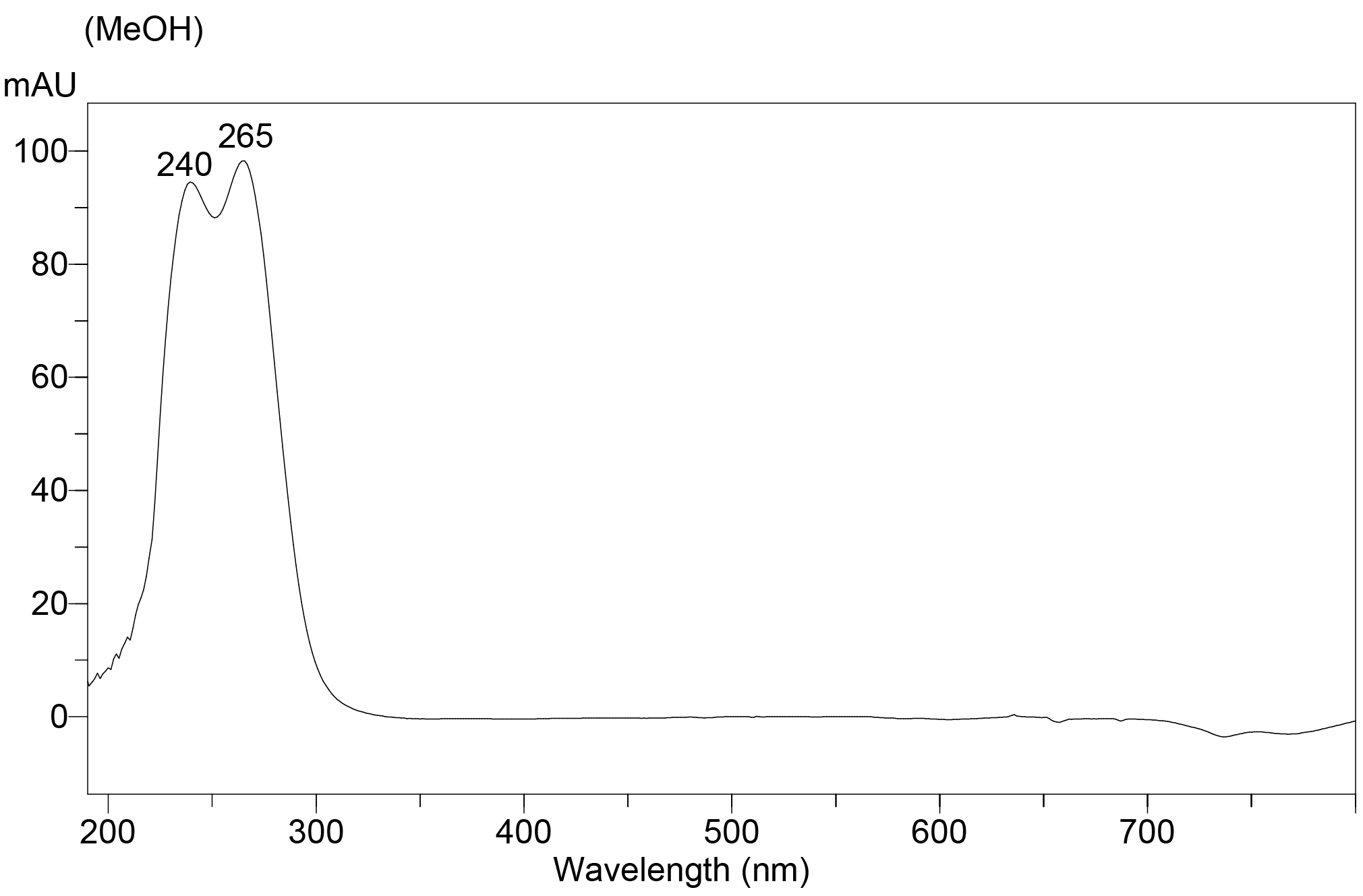
**

## **Figure S15.2** UV spectrum of **4**


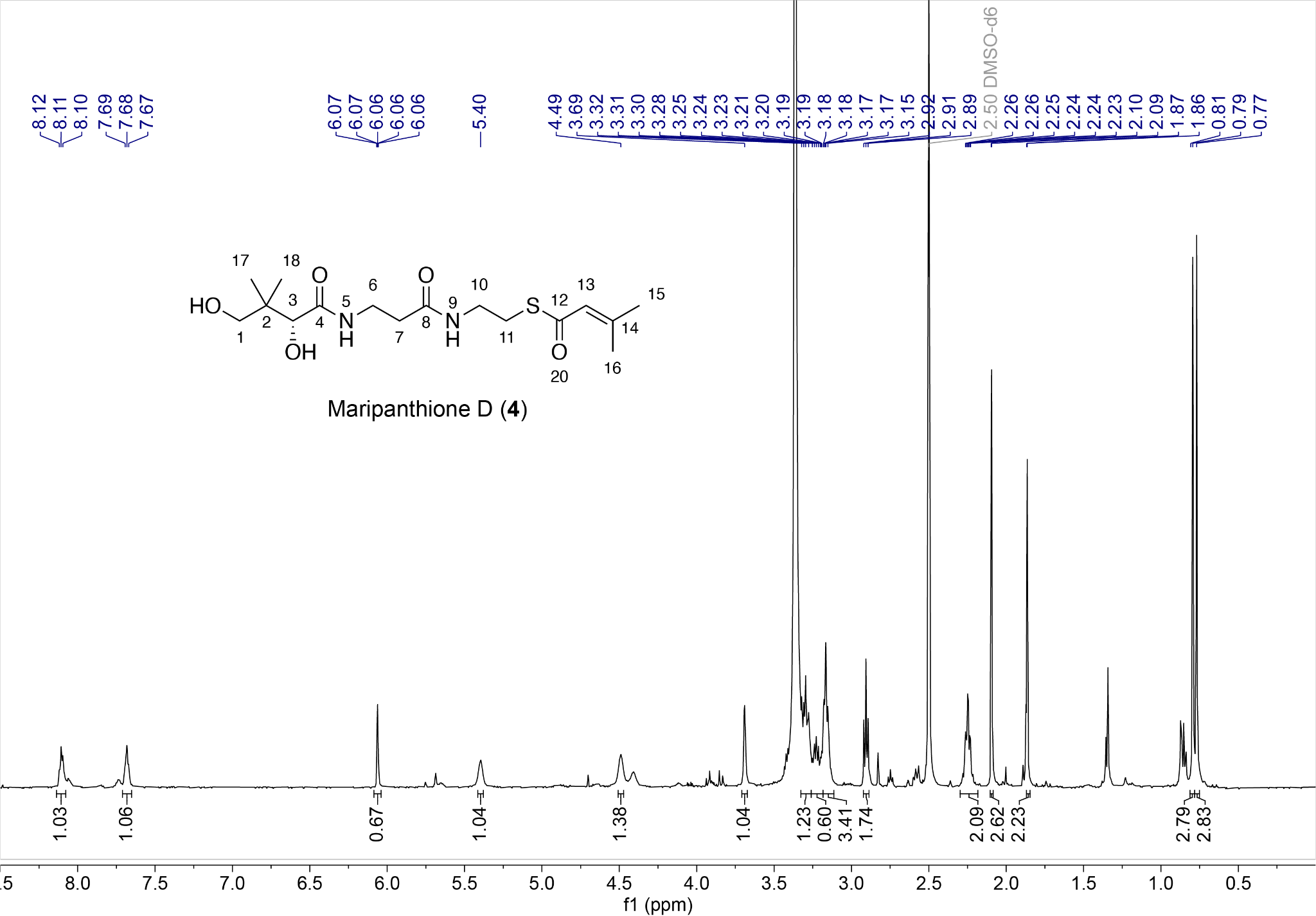


## **Figure S15.3** ^1^H NMR spectrum of **4** (500 MHz, DMSO-*d6*)


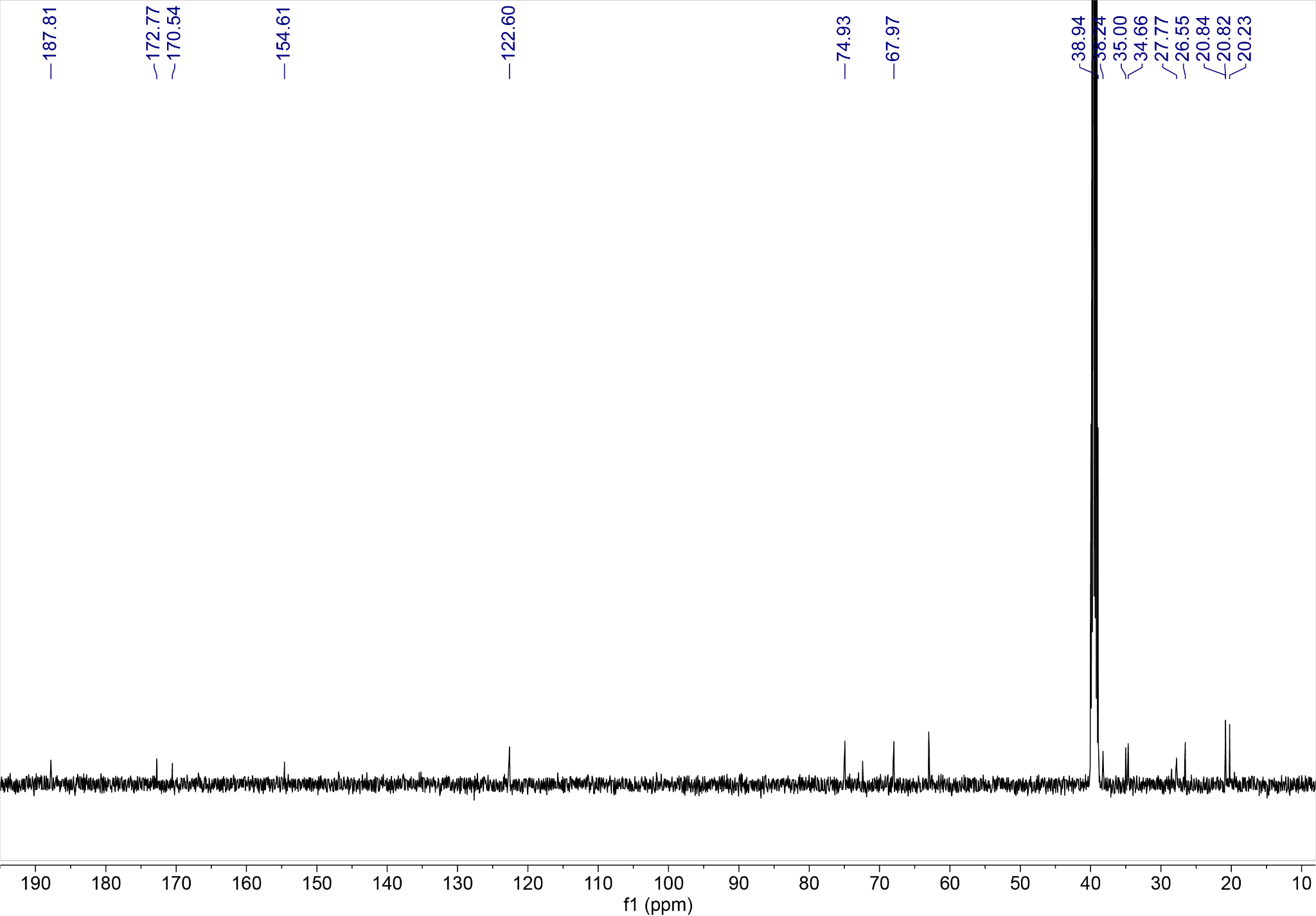


## **Figure S15.4** ^13^C NMR spectrum of **4** (125 MHz, DMSO-*d6*)


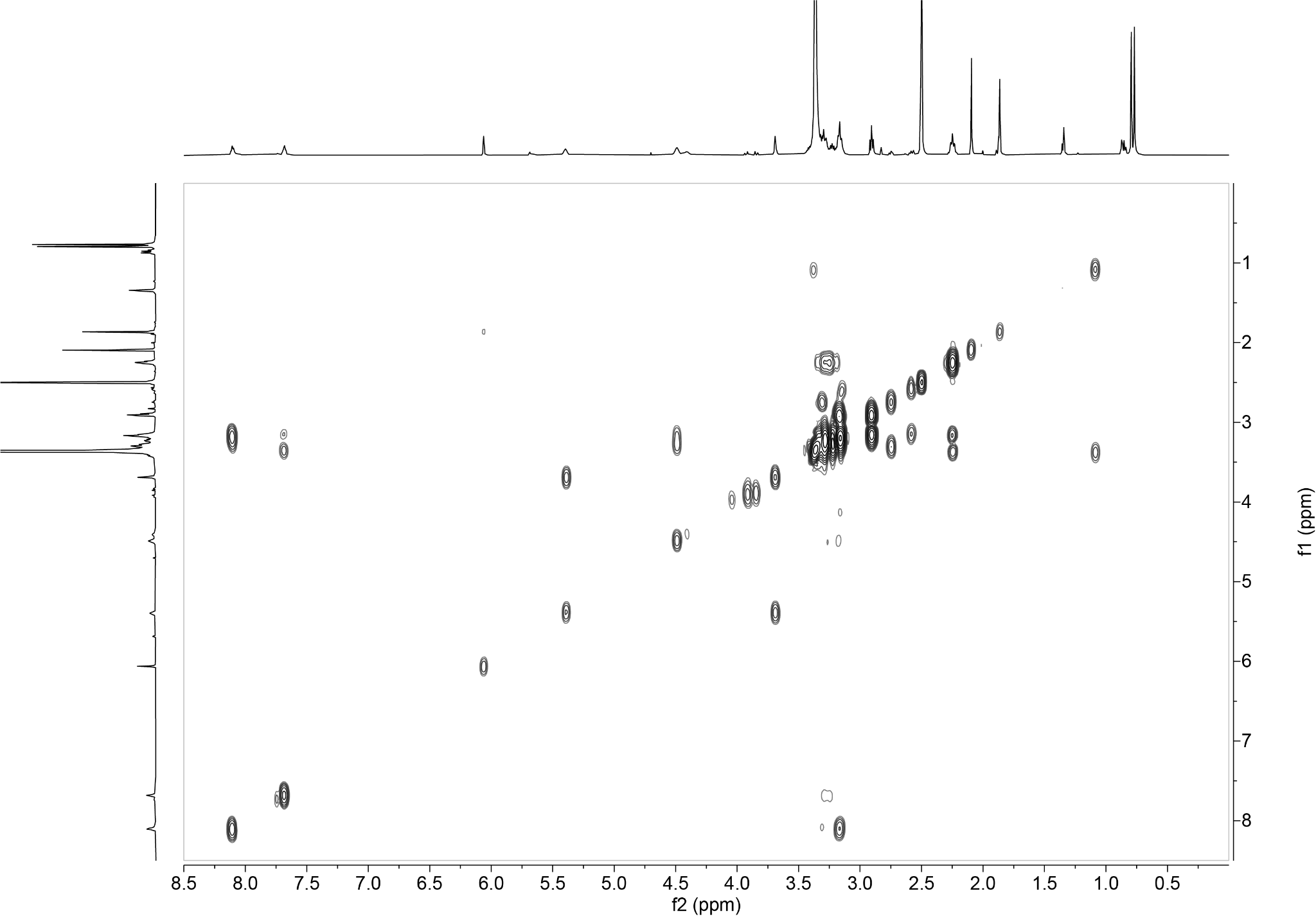


## **Figure S15.5** COSY spectrum of **4** (500 MHz, DMSO-*d6*)


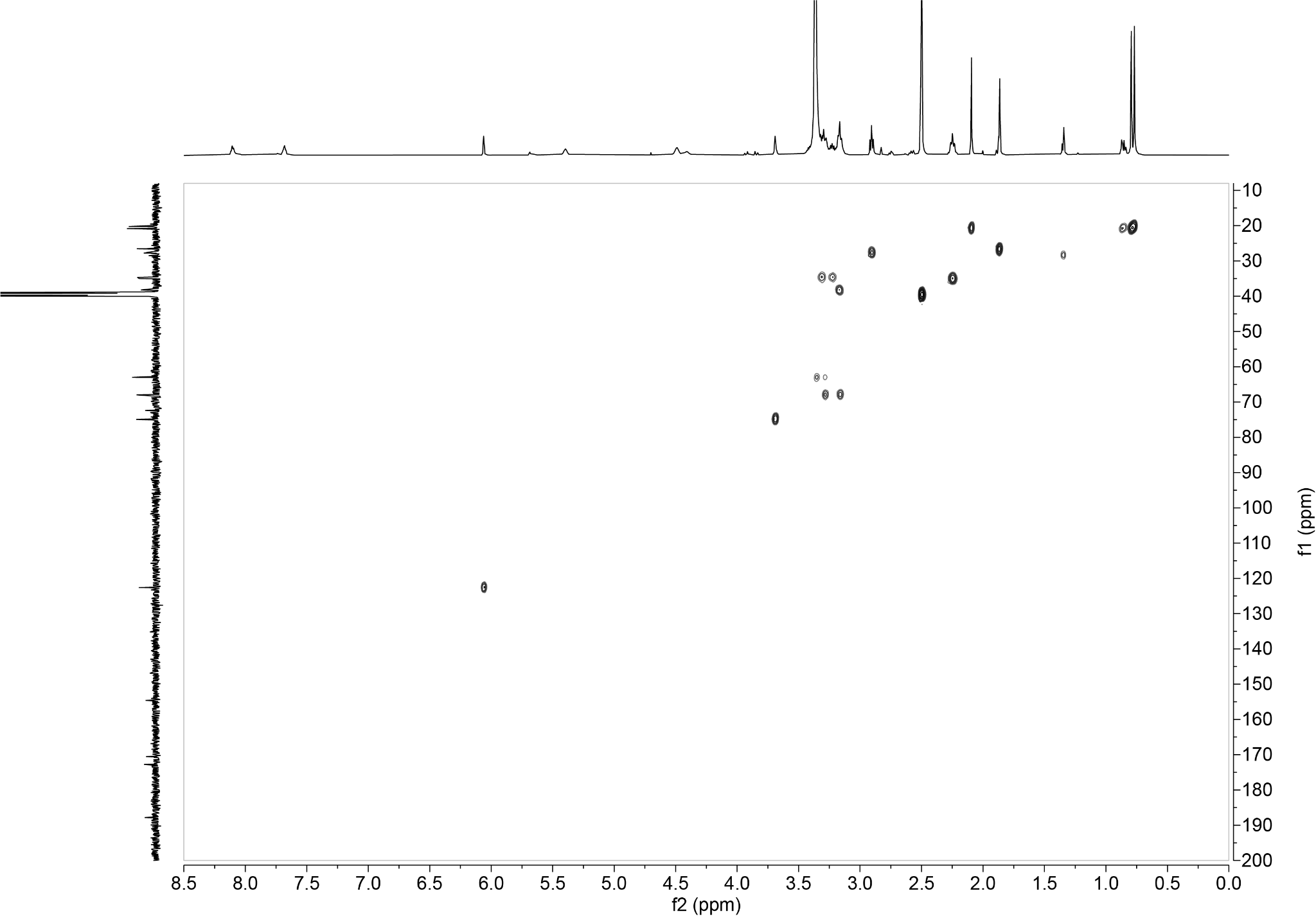


## **Figure S15.6** HSQC spectrum of **4** (500 MHz, DMSO-*d6*)


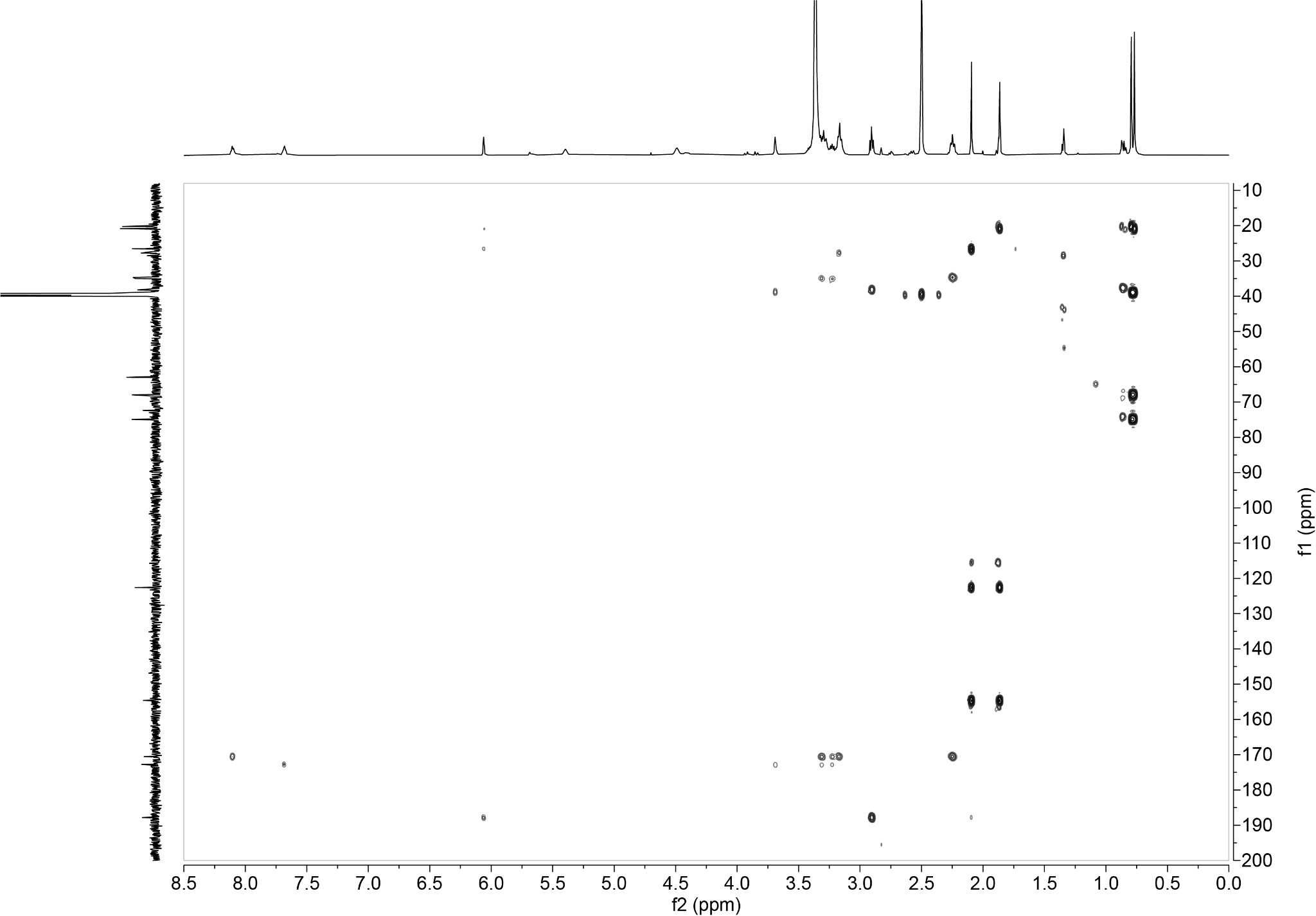


## **Figure S15.7** HMBC spectrum of **4** (500 MHz, DMSO-*d6*)


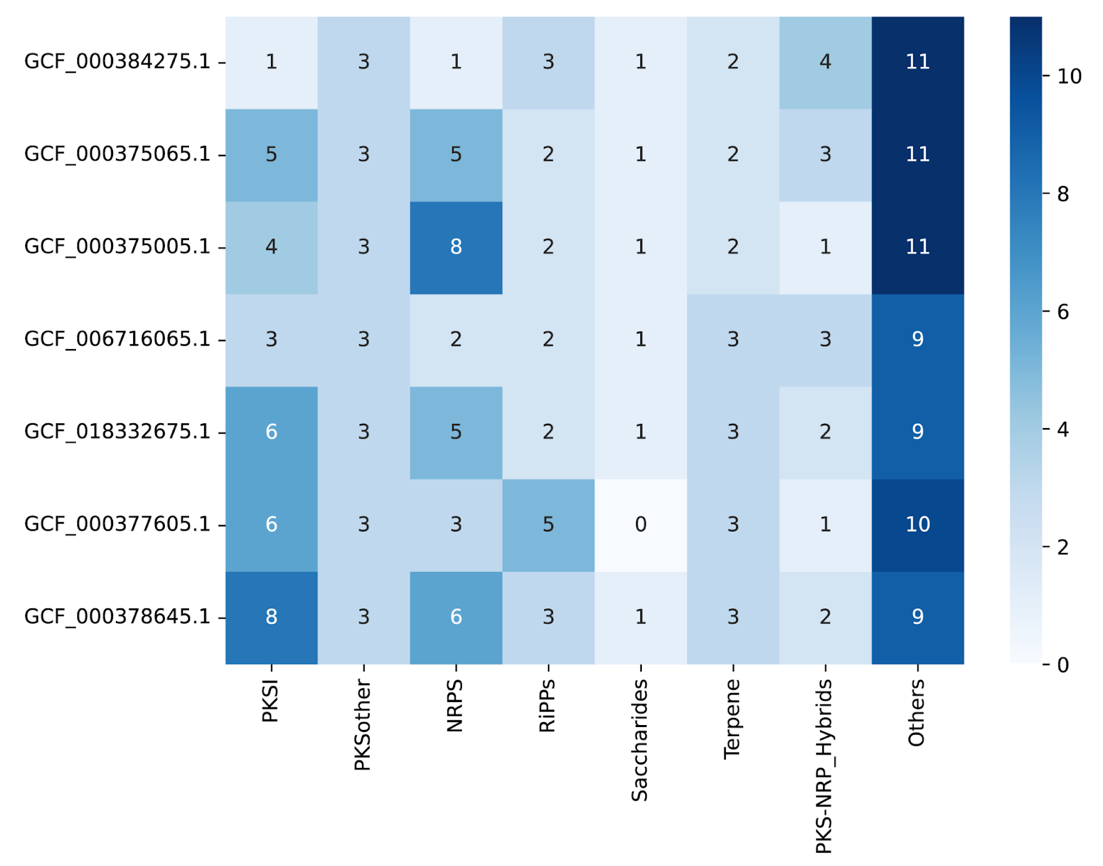


## **Figure S16** Heatmap of BGC profiles from seven *Salinispora* *arenicola* genomes sharing identical 16S rRNA sequences.

Supplementary Tables

## **Table S1** PSMPA vs. antiSMASH prediction comparison for *Marinobacterium* sp. YM272

| No. BGC | Sum | PKSI | PKSother | NRPS | RiPPs | Saccharides | Terpene | PKS-NRP Hybrids | Others |
| --- | --- | --- | --- | --- | --- | --- | --- | --- | --- |
| PSMPA | 12 | 0 | 1 | 0 | 6 | 0 | 0 | 0 | 5 |
| antiSMASH | 10 | 1 | 1 | 0 | 5 | 0 | 0 | 0 | 3 |

## **Table S2** ^1^H and ^13^C NMR Data for **1** and **2**


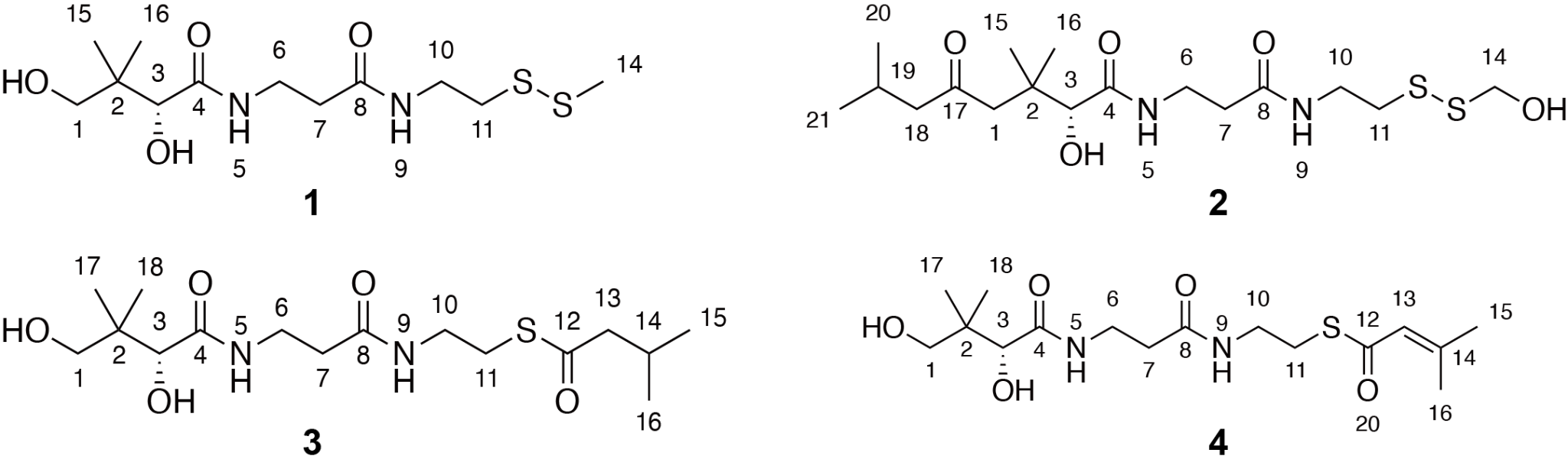


|  | **1** (DMSO-*d6*) | |  | **2** (DMSO-*d6*) | |
| --- | --- | --- | --- | --- | --- |
| No. | *δ*_C_, type | *δ*_H_, mult (J in Hz) |  | *δ*_C_, type | *δ*_H_, mult (J in Hz) |
| 1 | 68.0, CH_2_ | 3.16, o  3.28, o |  | 69.4, CH_2_ | 3.85, d (10.54)  3.91, d (10.54) |
| 2 | 39.0, C |  |  | 37.6, C |  |
| 3 | 74.9, CH | 3.69, d (5.41) |  | 74.2, CH | 3.68, d (5.72) |
| 4 | 172.8, C |  |  | 171.8, C |  |
| 6 | 34.7, CH_2_ | 3.23, o  3.32, o |  | 34.7, CH_2_ | 3.23, o  3.31, o |
| 7 | 35.0, CH_2_ | 2.26, td (7.05, 2.67) |  | 35.0, CH_2_ | 2.27, td (7.10, 2.79) |
| 8 | 170.6, C |  |  | 170.5, C |  |
| 10 | 37.8, CH_2_ | 3.32, o |  | 37.8, CH_2_ | 3.32, o |
| 11 | 36.3, CH_2_ | 2.76, t (6.87) |  | 36.3, CH_2_ | 2.77, t (6.87) |
| 14 | 22.6, CH_3_ | 2.40, s |  | 48.5, CH_2_ | 3.17, d (5.25) |
| 15 | 20.2, CH_3_ | 0.77, s |  | 20.8, CH_3_ | 0.87, s |
| 16 | 20.8, CH_3_ | 0.79, s |  | 20.3, CH_3_ | 0.86, s |
| 17 |  |  |  | 172.1, C |  |
| 18 |  |  |  | 42.7, CH_2_ | 2.18, d (6.77) |
| 19 |  |  |  | 25.1, CH | 1.99, dp (13.67, 6.85) |
| 20 |  |  |  | 22.1, CH_3_ | 0.91, s |
| 21 |  |  |  | 22.1, CH_3_ | 0.90, s |
| 1-OH/14-OH |  | 4.49, t (5.60) |  |  | 4.09, m |
| 3-OH |  | 5.38, d (5.48) |  |  | 5.62, d (5.71) |
| 5-NH |  | 7.69, t (5.96) |  |  | 7.73, t (5.91) |
| 9-NH |  | 8.01, t (5.64) |  |  | 8.08, t (5.68) |

## **Table S3** ^1^H and ^13^C NMR Data for **3** and **4**

**
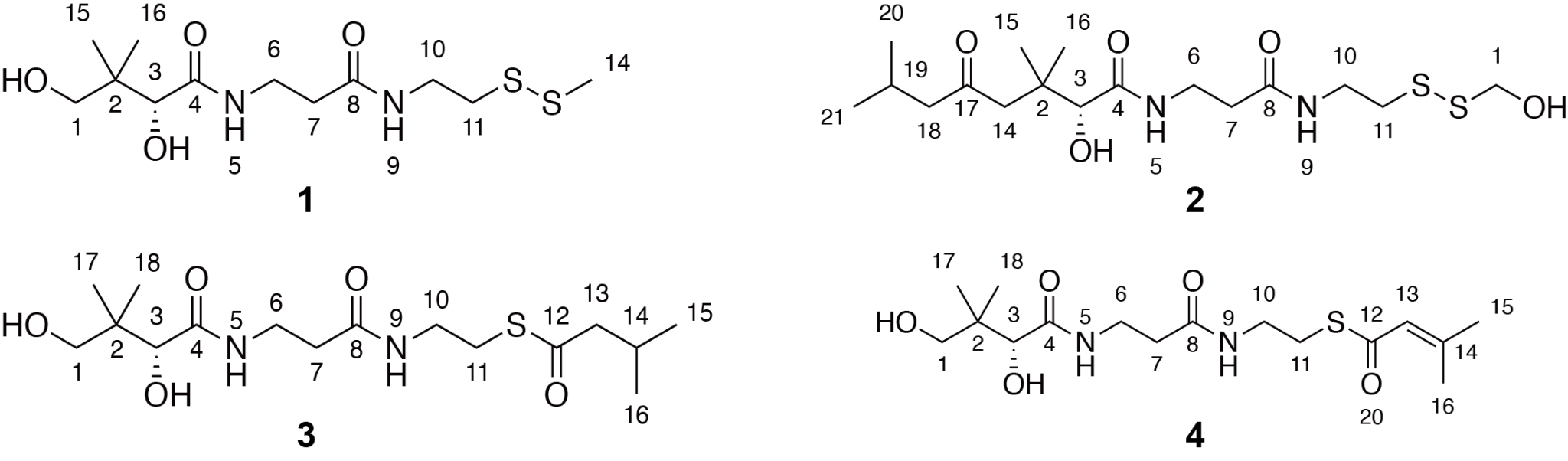
**

|  | **3** (DMSO-d6) | |  | **4** (DMSO-d6) | |
| --- | --- | --- | --- | --- | --- |
| no. | *δ*_C_, type | *δ*_H_, mult (J in Hz) |  | *δ*_C_, type | *δ*_H_, mult (J in Hz) |
| 1 | 68.0, CH_2_ | 3.17, o  3.30, o |  | 68.0, CH_2_ | 3.17, o  3.29, o |
| 2 | 39.0, C |  |  | 38.9, C |  |
| 3 | 75.0, CH | 3.69, d (5.47) |  | 74.9, CH | 3.69, d (4.60) |
| 4 | 172.8, C |  |  | 172.8, C |  |
| 6 | 34.7, CH_2_ | 3.22, o  3.30, o |  | 34.7, CH_2_ | 3.21, o  3.29, o |
| 7 | 35.1, CH_2_ | 2.24, td (7.06, 2.61) |  | 35.0, CH_2_ | 2.25, td (7.20, 3.14) |
| 8 | 170.6, C |  |  | 170.5, C |  |
| 10 | 38.2, CH_2_ | 3.17, o |  | 38.2, CH_2_ | 3.17, o |
| 11 | 28.0, CH_2_ | 2.89, t (6.90) |  | 27.8, CH_2_ | 2.91, t (6.85) |
| 12 | 198.0, C |  |  | 187.8, C |  |
| 13 | 52.1, CH_2_ | 2.45, d (7.10) |  | 122.6, CH | 6.06, p (1.25) |
| 14 | 26.0, CH | 2.03, m |  | 154.6, C |  |
| 15 | 21.9, CH_3_ | 0.89, d (6.68) |  | 20.8, CH_3_ | 2.10, d (1.30) |
| 16 | 21.9, CH_3_ | 0.89, d (6.68) |  | 26.5, CH_3_ | 1.87, d (1.34) |
| 17 | 20.9, CH_3_ | 0.79, s |  | 20.8, CH_3_ | 0.79, s |
| 18 | 20.3, CH_3_ | 0.77, s |  | 20.2, CH_3_ | 0.77, s |
| 1-OH |  | 4.47, t (5.59) |  |  | 4.49, t (5.41) |
| 3-OH |  | 5.37, d (5.52) |  |  | 5.40, d (5.51) |
| 5-NH |  | 7.68, t (5.92) |  |  | 7.69, br t (5.85) |
| 9-NH |  | 8.09, t (5.73) |  |  | 8.11, br t (5.65) |

## **Table S4** Annotation results in FBMN

| Compound ID (Node ID) | Compound name | MQscore | MZErrorPPM |
| --- | --- | --- | --- |
| S1 (47, 53, 63) | 1,3-diphenylguanidine | 0.76, 0.79, 0.84 | 4.68, 4.68, 0 |
| S2 (319) | oleamide | 0.75 | 3.89 |
| S3 (20) | cyclo(phe-4-hydroxy-pro) | 0.77 | 5.38 |
| S4 (9) | cyclo(L-leu-L-4-hydroxy-pro) | 0.89 | 4.43 |
| S5 (42) | cyclo(L-phe-D-pro) | 0.94 | 0.81 |
| S6 (27, 32) | cyclo(leu-pro) | 0.83, 0.93 | 3.32, 3.32 |
| S7 (3) | cyclo(tyr-pro) | 0.81 | 1.05 |
| S8 (5) | cyclo(L-val-L-pro) | 0.98 | 7.12 |
| S9 (244) | lauryldiethanolamine | 0.93 | 3.23 |
| S10 (289, 303) | 1-(9Z-octadecenoyl)-sn-glycero-3-phosphoethanolamine | 0.75, 0.87 | 1.22, 4.74 |
| S11 (376) | phytosphingosine | 0.89 | 3.74 |
| S12 (214) | linolenic acid | 0.81 | 5.03 |
| S13 (387) | dioctyl phthalate | 0.88 | 1.92 |
| S14 (61) | N-(3-methylbut-2-en-1-yl)-7H-purin-6-amine | 0.93 | 7.33 |

## **Table S5** MS/MS data of **1** (Precursor: [M+Na]^+^ = 347.1069)

| Mass/Charge | Intensity |
| --- | --- |
| 96.0544008 | 4254 |
| 139.083429 | 45386 |
| 196.09356 | 12982 |
| 197.102021 | 2310 |
| 217.043637 | 9798 |
| 267.131703 | 5148 |
| 329.09586 | 8452 |
| 347.105916 | 17031 |

## **Table S6** MS/MS data of **2** (Precursor: [M+Na]^+^ = 431.1635)

| Mass/Charge | Intensity |
| --- | --- |
| 122.057653 | 3341 |
| 139.083359 | 42450 |
| 145.043051 | 2432 |
| 157.042997 | 5830 |
| 180.111816 | 10041 |
| 196.082316 | 3506 |
| 217.043509 | 16767 |
| 266.1362 | 5005 |
| 273.034032 | 2997 |
| 280.150683 | 36137 |
| 281.159309 | 2200 |
| 307.114096 | 9652 |
| 329.095787 | 12679 |
| 337.172971 | 10889 |
| 351.188615 | 13311 |
| 416.140959 | 2579 |
| 431.164422 | 24897 |
| 432.166612 | 4195 |

## **Table S7** MS/MS data of **3** (Precursor: [M+H]^+^ = 363.1957)

| Mass/Charge | Intensity |
| --- | --- |
| 57.0704 | 7463 |
| 72.0450 | 3845 |
| 78.0377 | 3055 |
| 85.0654 | 10816 |
| 102.0377 | 4082 |
| 114.0916 | 3165 |
| 131.0646 | 4203 |
| 132.0485 | 11181 |
| 149.0746 | 38702 |
| 156.1020 | 33612 |
| 159.0597 | 3727 |
| 233.1316 | 12285 |
| 261.1274 | 6807 |
| 345.1838 | 12646 |

## **Table S8** MS/MS data of **4** (Precursor: [M+H]^+^ = 361.1792)

| Mass/Charge | Intensity |
| --- | --- |
| 55.0538 | 10645 |
| 83.0479 | 43842 |
| 131.0639 | 2836 |
| 132.0475 | 13574 |
| 142.0860 | 3671 |
| 149.0736 | 28817 |
| 154.0851 | 28286 |
| 231.1150 | 10032 |
| 261.1254 | 23618 |
| 343.1671 | 11609 |

## **Table S9.** Details of the bioactivity of maripanthione D (**4**) as reported in patent WO2020113209

| Example | Cell (& Cell Line) | Assay Condition | Compound Concentration used | Effect |
| --- | --- | --- | --- | --- |
| Effect of Compounds on mitochondrial respiration (Example 850) | PA (Propionic Acidemia), Tsi 6337 | Supplements in DMEM: 10 mM glucose, 2 mM L-glutamine, 1 mM pyruvate, 10% FBS. FCCP: 8 µM. Post-FCCP cycles: 20. | 10 µM | Maximum respiration increased by at least 10% |
| Effect of Compounds on mitochondrial respiration (Example 851) | PA, Tsi 6337 | Supplements in DMEM: 1 mM glucose, 2 mM L-glutamine, 1 mM pyruvate, 10% FBS. FCCP: 8 µM. Post-FCCP cycles: 20. | 10 µM | Maximum respiration increased by at least 30% |
| Effect of Compounds on mitochondrial respiration (Example 853) | MMA (Methylmalonic Acidemia), Tsi 5224 | Supplements in DMEM: 10 mM glucose, 2 mM L-glutamine, 1 mM pyruvate, 10% FBS. FCCP: 4 µM. Post-FCCP cycles: 20. | 10 µM | Spare respiratory capacity increased by at least 10% |
| Effect of Compounds on mitochondrial respiration (Example 854) | MMA, Tsi 5224 | Supplements in DMEM: 10 mM glucose, 2 mM L-glutamine, 1 mM pyruvate, 10% FBS. FCCP: 4 µM. Post-FCCP cycles: 20. | 10 µM | Spare respiratory capacity increased by at least 30% |
| Effect of Compounds on mitochondrial respiration (Example 855) | PA, Tsi 6337 | Supplements in DMEM: 1 mM glucose, 2 mM L-glutamine, 1 mM pyruvate, 10% FBS. FCCP: 8 µM. Post-FCCP cycles: 20. | 10 µM | Spare respiratory capacity increased by at least 50% |
| M1 Differentiation Assay (Example 861) | Primary CD14+ monocytes | Medium: RPMI complete media with 15% FBS, 1% Penicillin-Streptomycin and 10 ng/mL GM-CSF for differentiation. | 50 µM | Macrophage IL-6 secretion decreased by at least 30% |
| M1 Differentiation Assay (Example 862) | Primary CD14+ monocytes | Medium: RPMI complete media with 15% FBS, 1% Penicillin-Streptomycin and 10 ng/mL GM-CSF for differentiation. | 10 µM | IL-10 secretion decreased by at least 30% |
| M1 Differentiation Assay (Example 866) | Primary CD14+ monocytes | Medium: RPMI complete media with 15% FBS, 1% Penicillin-Streptomycin and 10 ng/mL GM-CSF for differentiation. | 10 µM | Macrophage TNFα secretion decreased by at least 30% |
| Measurement of ROS (Example 882) | KSS (Kearns-Sayre Syndrome), GM06225 | Supplements in DMEM: 1 mM glucose, 10% FBS. | 10 µM | Reducing ROS by at least 10% |
| Measurement of ROS (Example 883) | VLCFA (Impaired VLCFA oxidation), GM13262 | Supplements in DMEM: 1 mM glucose, 10% FBS. | 10 µM | Reducing ROS by at least 30% |

#

# References

[1] Douglas GM, Maffei VJ, Zaneveld JR, Yurgel SN, Brown JR, Taylor CM, et al. PICRUSt2 for prediction of metagenome functions. Nat Biotechnol 2020;38:685–8. https://doi.org/10.1038/s41587-020-0548-6.

[2] Blin K, Shaw S, Steinke K, Villebro R, Ziemert N, Lee SY, et al. antiSMASH 5.0: updates to the secondary metabolite genome mining pipeline. Nucleic Acids Research 2019;47:W81–7. https://doi.org/10.1093/nar/gkz310.

[3] Navarro-Muñoz JC, Selem-Mojica N, Mullowney MW, Kautsar SA, Tryon JH, Parkinson EI, et al. A computational framework to explore large-scale biosynthetic diversity. Nat Chem Biol 2020;16:60–8. https://doi.org/10.1038/s41589-019-0400-9.

[4] Zong G, Fu J, Zhang P, Zhang W, Xu Y, Cao G, et al. Use of elicitors to enhance or activate the antibiotic production in *streptomyces*. Critical Reviews in Biotechnology 2022;42:1260–83. https://doi.org/10.1080/07388551.2021.1987856.

[5] de Felício R, Ballone P, Bazzano CF, Alves LFG, Sigrist R, Infante GP, et al. Chemical Elicitors Induce Rare Bioactive Secondary Metabolites in Deep-Sea Bacteria under Laboratory Conditions. Metabolites n.d.;11:107. https://doi.org/10.3390/metabo11020107.

[6] Ma M, Lohman JR, Liu T, Shen B. C-S bond cleavage by a polyketide synthase domain. Proc Natl Acad Sci USA 2015;112:10359–64. https://doi.org/10.1073/pnas.1508437112.
